# Two MYB and Three bHLH Family Genes Participate in Anthocyanin Accumulation in the Flesh of Peach Fruit Treated with Glucose, Sucrose, Sorbitol, and Fructose In Vitro

**DOI:** 10.3390/plants11040507

**Published:** 2022-02-13

**Authors:** Jiao Wang, Ke Cao, Lirong Wang, Wenxuan Dong, Xiao Zhang, Weisheng Liu

**Affiliations:** 1 College of Horticulture, Shenyang Agricultural University, Shenyang 110866, China; wangjiaolingling@163.com (J.W.); dongwx63@syau.edu.cn (W.D.); zhangxiao8866@syau.edu.cn (X.Z.); 2 The Key Laboratory of Biology and Genetic Improvement of Horticultural Crops (Fruit TreeBreeding Technology), Ministry of Agriculture, Zhengzhou Fruit Research Institute, Chinese Academy of Agricultural Sciences, Zhengzhou 450009, China; caoke@caas.cn (K.C.); wanglirong@caas.cn (L.W.)

**Keywords:** anthocyanin, bHLH TF, dual luciferase assay, MYB TF, sugar treatment

## Abstract

Anthocyanins are important pigments in peach fruit and are beneficial to human health. Sugars are both energy-storing and signaling molecules and their roles in inducing anthocyanin biosynthesis have received a great deal of research attention. However, the mechanism by which sugars induce anthocyanin biosynthesis in peach fruit is unknown. In order to understand this induction mechanism, comprehensive transcriptome and metabolome were performed in fruit flesh treated with four different sugars for 12 and 24 h, respectively. Here, we found that cyanidin-3-*O*-(6-*O*-p-coumaroyl) glucosides accumulated in fruit flesh treated with glucose, sucrose, sorbitol, and fructose in vitro. Two key structural genes of the anthocyanin biosynthesis pathway, namely, *PpDFR* and *PpUFGT*, were upregulated in the flesh of sugar-treated peach fruit. By contrast, the two main transcription factors (TFs) *PpMYB10.1* and *PpBL* regulating anthocyanin biosynthetic genes in peach fruit were not upregulated accordingly. Interestingly, two MYB family genes (*PpMYB6* and *PpMYB44-like)* and three bHLH family genes (*PpbHLH35*, *PpbHLH51*, and *PpbHLH36-like)* were upregulated. A dual luciferase assay revealed that PpMYB6 strongly activated the *PpUFGT* promoter when it was co-infiltrated with PpbHLH35, PpbHLH51, and PpbHLH36-like. When *PpMYB44-like* was co-infiltrated with *PpbHLH35*, it also potently activated the PpUFGT promoter. The results of this study help clarify the molecular mechanisms by which glucose, sucrose, sorbitol, and fructose regulate anthocyanin accumulation in peach fruit.

## 1. Introduction

Anthocyanins are flavonoid end-products and are abundant in leaves, flowers, fruits, and seeds. Their colors range from red to blue-purple in these plant organs [1,2]. The anthocyanin core structures are aglycones and include pelargonidin, cyanidin, delphinidin, peonidin, and malvidin [3,4]. Anthocyanins cause red pigmentation which improves fruit appearance and attracts insects and animals that propagate seeds [5,6]. They also play vital roles in plant resistance to biotic stress (such as pest insect infestations and microbial pathogen infection) and abiotic stress (such as drought and salinity) [7].

Anthocyanins are synthesized in the cytoplasm and then vacuolated via glutathione S-transferase (GST) [8]. Anthocyanin biosynthesis is catalyzed by a multi-enzyme complex including phenylalanine ammonia-lyase (PAL), cinnamate-4-hydroxylase (C4H), 4-coumaroyl:CoA-ligase (4CL), chalcone synthase (CHS), chalcone flavanone isomerase (CHI), flavanone 3-hydroxylase (F3H), flavonoid 3′-hydroxylase (F3′H), dihydroflavonol 4-reductase (DFR), leucoanthocyanidin dioxygenase (LDOX), UDP-glucose: flavonoid 3-O-glucosyltransferase (UFGT), and glutathione *S*-transferase (GST) [9].

Anthocyanin biosynthesis is regulated at the transcriptional level by R2R3-myeloblastosis (MYB), basic helix–loop–helix (bHLH), and WD40 that form the MBW complex [8]. In apples, the MYB transcription factors MdMYB10 and MdMYB110a control anthocyanin accumulation by forming homodimers with MdbHLH [10]. In grape, several MYB transcription factors have been identified, such as VvMYBA1-1, VvMYBA1-2, VvMYBA1, and VvMYBA2. Of these, VvMYBA1 and VvMYBA2 regulate anthocyanin accumulation in the fruit skin [11,12]. In citrus, the MYB transcription factor CcRuby plays an important role in the red flesh trait [13]. PyMYB10, PyMYB114, and PybHLH3 in pear [14,15], FvMYB10 and FvbHLH33 in wild strawberry [16], and AtMYB75 (PAP1), AtMYB90 (PAP2), AtMYB113, and AtMYB114 in Arabidopsis [9,17] participate in anthocyanin accumulation.

Sugars are both energy-storing and signaling molecules and their roles in promoting anthocyanin biosynthesis have received extensive research attention. The shoots of *Clematis pitcheri* cultured in the presence of high sucrose concentrations and nitrogen accumulated high anthocyanin levels [18]. Exogenous sucrose induced anthocyanin accumulation more effectively than a 1:1 fructose: glucose mixture in the hypocotyls and roots of red radish seedlings [19]. Glucose more effectively induced anthocyanin accumulation than sucrose and without exogenous sugar treatment in blackberry fruit [20]. Strawberry fruit treated with sucrose displayed high levels of pelargonidin derivatives and upregulation of the genes participating in the phenylpropanoid and flavonoid pathways [21]. In Arabidopsis, sucrose induces anthocyanin accumulation in a concentration-dependent manner [22].

Anthocyanins accumulate in two ways during peach fruit development. First, peak anthocyanin content occurred at the late stages of fruit development in the blood-flesh peach cultivars ‘Beijingyixianhong’, ‘Wuyuexian’, and ‘Tianjin Shui Mi’. The associated anthocyanin biosynthesis-encoding genes *PpPAL*, *PpUFGT*, *PpCHI*, *PpF3H*, *PpDFR*, *PpANS,* and *PpCHS* reached their highest transcription levels during the early-to-middle stages of fruit development [2,23]. Second, the anthocyanin content reached its highest levels during the early stages of fruit development in the blood-flesh peach cultivars ‘Heiyoutao,’ ‘Sanguine Pilat,’ and ‘Sanguine Vineuse.’ The aforementioned anthocyanin biosynthesis-encoding genes had lower transcription levels in these cultivars than they did in ‘Beijingyixianhong’ and ‘Wuyuexian’ [23,24]. Both anthocyanin accumulation mechanisms are determined by two alleles. The former is determined by the NAC family gene *PpBL* mapped atop linkage group five. PpBL and MYB10.1 promote the transcription of the anthocyanin biosynthesis-regulating genes which leads to anthocyanin accumulation in blood-flesh fruit [25,26]. The latter is determined by a recessive locus (*bf*) mapped to linkage group four [23]. However, cyanidin-3-glucoside was the main anthocyanin component in both types of blood-flesh peach fruit.

Glucose, sucrose, sorbitol and fructose are the major sugar compositions in peach fruit [27]. Sugar induction of anthocyanin in fruit is very important, it will provide a scientific basis to promote the accumulation of anthocyanin in fruits, and also an important way to achieve the goal of regulating fruit color development. Studies on induction of peach fruit by different sugars have been reported. For example, sucrose more effectively stimulated anthocyanin accumulation than either glucose or fructose in media-cultured red leaf peach shoots [28]. In the ‘Tenshin Suimitsuto’ blood-flesh peach fruit cultivar, 100 mM sucrose induced anthocyanin biosynthesis [29].

In summary, the anthocyanins biosynthesis and regulation have been studied very thoroughly in peach and other species. Moreover, sugar as signaling molecules also have been received extensive attention in promoting anthocyanin biosynthesis in many horticultural crops. However, few studies have been focused on peach anthocyanins induction by exogenous sugars and the corresponding induction mechanism. In the present study, we mainly focused on how different sugars induce anthocyanins accumulation in peach fruit flesh. We subjected the blood-flesh peach fruit cultivar ‘Tianjin ShuiMi’ to various sugar treatments. We found two MYB family genes: *PpMYB6* (*Prupe.5G065500*), *PpMYB44-like* (*Prupe.8G134900*), and three bHLH family genes: *Pp bHLH35* (*Prupe.1G074400*), *Pp bHLH51* (*Prupe.2G252600*) and *Pp bHLH36-like* (*Prupe.3G131500*). (https://www.rosaceae.org/, (accessed on 12 July 2021)). Functional analysis indicates that these TFs have the potential in promoting anthocyanin biosynthesis in four different sugar treated fruits. This will help clarify the molecular mechanisms by which glucose, sucrose, sorbitol, and fructose regulate anthocyanin accumulation in peach fruit. *PpMYB6PpMYB44-likePpbHLH35PpbHLH51PpbHLH36-like*

## 2. Results

### 2.1. Levels of Metabolites Involved in Anthocyanin Biosynthesis

There were 24,228 metabolites in the flesh of peach fruit treated with sugars. Of these, 3923, 3091, 4038, and 3938 were upregulated, while 7537, 7699, 8067, and 7807 were downregulated in the flesh of peach fruit treated with glucose, sucrose, sorbitol, and fructose, respectively, for 12 h. Moreover, 5023, 5062, 4952, and 5490 were upregulated while 7602, 7444, 7734, and 7870 were downregulated in the flesh of peach fruit treated with glucose, sucrose, sorbitol, and fructose, respectively, for 24 h (Tables S1 and S2). The top 100 upregulated metabolites in the flesh of peach fruit treated with sugar for 12 h were associated with ‘Biosynthesis of secondary metabolites’, ‘Metabolic pathways’, and ‘Flavonoid biosynthesis’, while the top 100 downregulated metabolites in the flesh of peach fruit treated with sugar for 12 h were associated with ‘Biosynthesis of antibiotics’, ‘Metabolic pathways’, and ‘Zeatin biosynthesis’ (Figures S1, S2 and Tables S9–S16). Of all these metabolites detected from LCMS and high-resolution tandem mass spectrometer, the top 100 upregulated metabolites in the flesh of peach fruit treated with sugar for 24 h were associated with ‘Biosynthesis of secondary metabolites’, ‘Metabolic pathways’, ‘Biosynthesis of phenylpropanoids’, ‘Flavone and flavonol biosynthesis’, ‘Isoflavonoid biosynthesis’, and ‘Flavonoid biosynthesis’, while the top 100 downregulated metabolites in the flesh of peach fruit treated with sugar for 24 h were associated with ‘Biosynthesis of antibiotics’, ‘Biosynthesis of secondary metabolites’, and ‘Metabolic pathways’ (Figures S3, S4 and Tables S17–S24).

**Figure 1:**
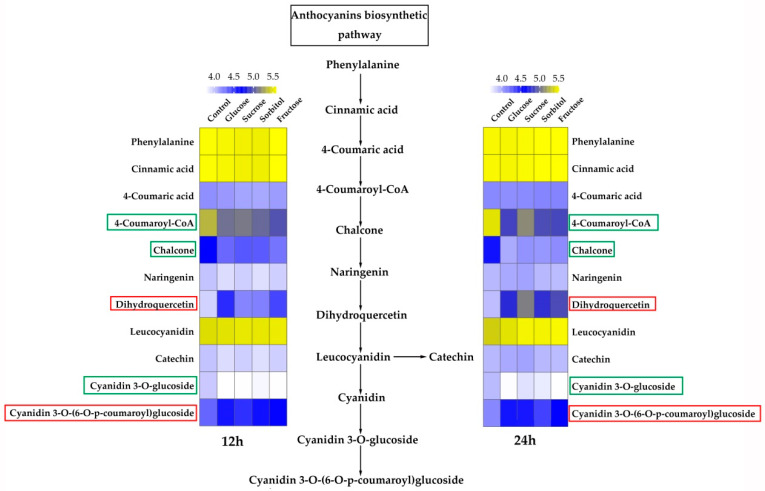
Metabolites involved in the anthocyanin biosynthesis pathway were determined in positive mode. Heat maps on the left and right indicate the metabolite content (M/Z) in the flesh of peach fruit treated with exogenous glucose, sucrose, sorbitol, and fructose for 12 h and 24 h, respectively. The numbers at the top of the picture indicate log10 (M/Z), which was listed in Tables S3 and S4. Metabolite names are shown on the side of the map. Metabolite content increases with blue and yellow color intensity in the square within the heat map. The red rectangle indicates that the metabolite content was higher in the flesh of the sugar-treated peach fruit than that of the untreated control. The green rectangle indicates that the metabolite content was lower in the flesh of the sugar-treated peach fruit than that of the untreated control.

As the sugar-treated fruit was picked from the ‘Tianjin Shui Mi’ blood-fleshed peach landrace, we focused mainly on flavonoids in the anthocyanin biosynthetic pathway. Figure 1, Tables S3 and S4 shows that the metabolites dihydroquercetin and cyanidin-3-*O*-(6-*O*-p-coumaroyl) glucoside were upregulated markedly in flesh samples of peach fruit treated with glucose, sucrose, sorbitol, and fructose for 12 h and 24 h. The p-coumaroyl CoA, chalcone, and cyanidin 3-*O*-glucoside levels were lower in the peach fruit treated with the sugars for 12 h and 24 h than they were in the control. All other metabolites had similar levels in both the sugar-treated and control peach fruit. These results indicated that glucose, sucrose, sorbitol, and fructose all had similar effects on metabolite accumulation in vitro. Moreover, after four sugars treatment for 12 and 24 h, anthocyanins were not accumulated as cyanidin 3-*O*-glucoside, the main components in blood-flesh peach fruits, but as cyanidin-3-*O*-(6-*O*-p-coumaroyl) glucoside.

### 2.2. Expression Levels of Genes Regulating the Anthocyanin Biosynthesis Pathway

**Figure 2:**
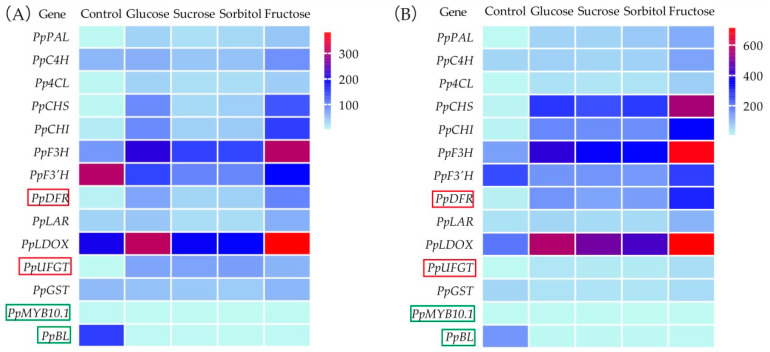
Heat map showing relative expression levels of anthocyanin biosynthesis and regulatory genes (FPKM) in flesh of sugar-treated and untreated peach fruit after 12 h (**A**) and 24 h (**B**). Gene names are shown on left side of map. Gene expression level increases with blue and red color intensity in the square within the heat map. PpDFR and PpUFGT are in red in the box. PpMYB10.1 and PpBL are in green in the box.

As anthocyanins accumulated in the flesh of the peach fruit treated with the sugars, we analyzed the expression of the genes involved in anthocyanin biosynthesis, based on the transcriptome data. Most of these genes were upregulated in the flesh of the peach fruit treated with sugars for 12 h and 24 h in vitro (Figure 2). The key structural genes *PpDFR* and *PpUFGT* were reported to be upregulated by the transcription factors PpMYB10.1 and PpBL, and their expression levels were also significantly higher in the flesh of the sugar-treated peach fruit compared with those of the control (Tables S5 and S6). By contrast, the key regulatory genes *PpMYB10.1* and *PpBL* were not upregulated in the flesh of the sugar-treated peach fruit compared with those of the control. In fact, *PpBL* was downregulated in the flesh of the sugar-treated peach fruit (Tables S5 and S6). Further quantitative PCR verification was performed, and the results were similar as that of the transcriptome data. Hence, we speculated that other transcription factors might upregulate anthocyanin biosynthesis-related genes in response to sugar induction.

### 2.3. Identification of Regulatory Genes Associated with High Expression Levels of Anthocyanin Biosynthesis-Related Genes in the Flesh of Sugar-Treated Peach Fruit

**Figure 3:**
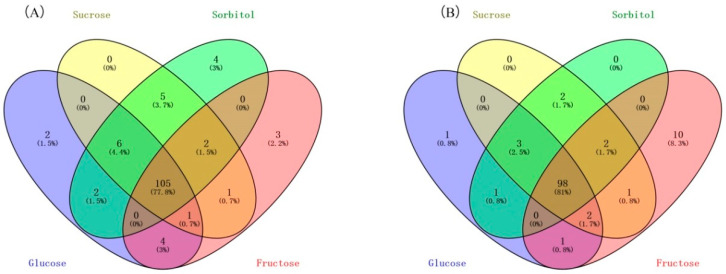
Venn diagrams showing numbers of upregulated TFs in flesh of glucose-, sucrose-, sorbitol-, and fructose-treated peach fruit after 12 h (**A**) and 24 h (**B**).

**Figure 4:**
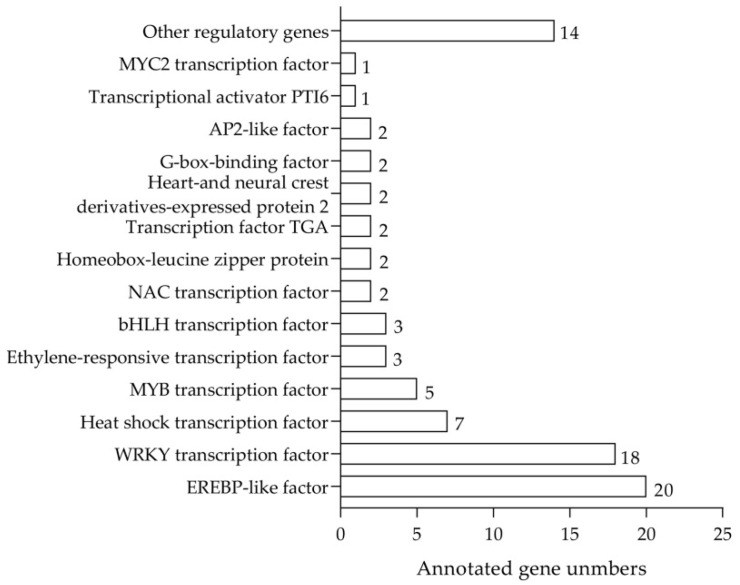
Eighty-four upregulated TFs common to flesh of peach fruit treated with glucose, sucrose, sorbitol, and fructose for 12 h and 24 h.

**Figure 5:**
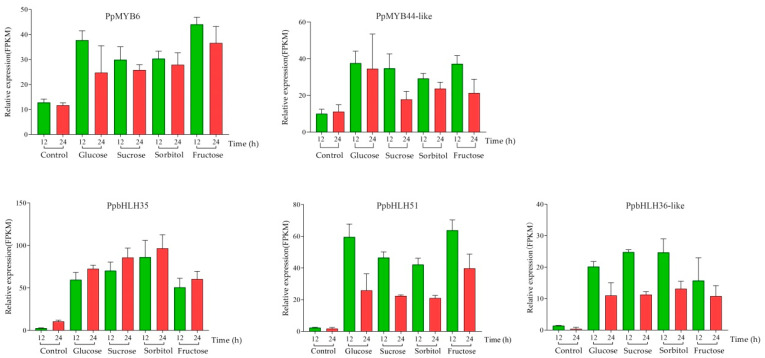
Relative expression levels of two MYB and three bHLH family genes selected among 84 upregulated TFs.

To identify the regulatory genes involved in anthocyanin biosynthesis in the flesh of sugar-treated peach fruit, we used the corresponding transcriptome data with fold change > 2.0 in the analysis. The results indicated that 120, 120, 124, and 116 of the upregulated TFs were identified in the flesh of the peach fruit treated with glucose, sucrose, sorbitol, and fructose, respectively, for 12 h. Then, 105 of these TFs were selected (Figure 3). Furthermore, 106, 108, 106, and 114 of the upregulated TFs were identified in the flesh of the peach fruit treated with glucose, sucrose, sorbitol, and fructose, respectively, for 24 h. Then, 98 of these TFs were selected, and 84 of the TFs common to both treatments had the potential in promoting anthocyanin biosynthesis, and these were thus selected for the subsequent analysis (Figure 4). Of these, the majority were WRKY- and EREBP-like gene family members and heat shock TFs ranked second. There were also eight MYB and bHLH gene family members. As MYB and bHLH family genes have been reported to regulate anthocyanin accumulation in the development of blood-flesh fruit [26], we selected two MYB genes designated *PpMYB6* and *PpMYB44-like* and three bHLH genes designated *PpbHLH35*, *PpbHLH51*, and *PpbHLH36-like* according to their relative high expression to determine whether they regulate anthocyanin accumulation (Figure 5).

### 2.4. Prediction of the Selected MYB and bHLH TFs Using a Tobacco Leaf Dual Luciferase Assay

**Figure 6:**
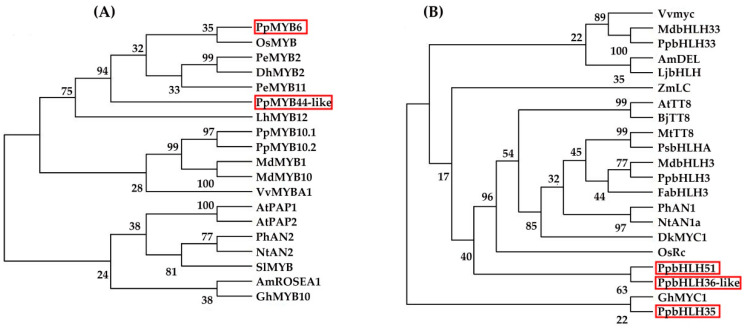
Phylogenetic analyses of amino acid sequences of MYB (**A**) and bHLH (**B**) TFs. Full MYB and bHLH TF amino acid sequence alignment and phylogenetic tree construction with MEGA-X software (Available online: https://www.megasoftware.net/dload_win_gui (accessed on 21 June 2021)). Numbers before binary structures indicate bootstrap test results for 1000-replicate analysis. PpMYB6, PpMYB44-like, PpbHLH35, PpbHLH51, and PpbHLH36-like are highlighted by solid red rectangles. National Center for Biotechnology Information (NCBI) accession numbers are as follows: *O*sMYB (CAA45509); PeMYB2 (AIS35919); DhMYB2 (AQS79852); PeMYB11 (AIS35928); LhMYB12 (BAJ05398); MdMYB1 (ABK58138); MdMYB10 (ABB84753); VvMYBA1 (BAD18977); AtPAP1 (NP-176057); AtPAP2 (NP-176813); PhAN2 (AAF66727); NtAN2 (AC*O*52472); SlMYB (AAQ55181); AmR*O*SEA1 (ABB83826); GhMYB10 (CAD87010); VvMYC (NP-001267954.1); MdbHLH33 (ABB84474.1); AmDEL (AAA32663.1); LjbHLH (BAJ10680.1); ZmLC (P13526.1); AtTT8 (*O*A*O*98324.1); BjTT8 (AIN41653.1); MtTT8 (AKN796061); PsbHLHA (E3SXU4.1); MdbHLH3 (ADL36597.1); FabHLH3 (AFL02463.1); PhAN1 (AAG259281); NtAN1a (AEE992571); DkMYC1 (AEC03343.1); *O*sRc (BAF42667); and GhMYC1 (CAA07615).

To validate PpMYB6, PpMYB44-like, PpbHLH35, PpbHLH51, and PpbHLH36-like regulation in anthocyanin biosynthesis, we analyzed their phylogenetic relationships with other MYB and bHLH TFs that are known to upregulate this process (Figure 6A). PpMYB6 was phylogenetically related to *O*sMYB while Pp.8G134900 was more closely associated with PeMYB11. The bHLH TFs PpbHLH35, PpbHLH51, and PpbHLH36-like were clustered together and were near GhMYC1 (Figure 6B). Therefore, PpMYB6, Pp.8G134900, PpbHLH35, PpbHLH51, and PpbHLH36-like probably activated anthocyanin biosynthesis.

**Figure 7:**
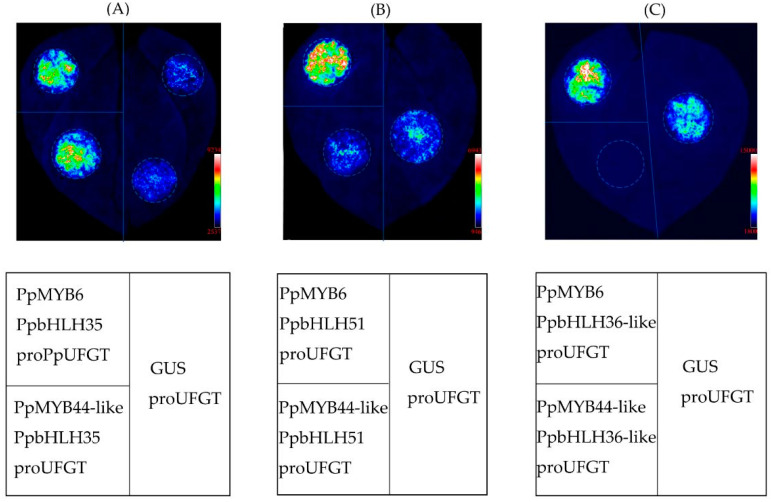
Regulatory relationships among selected MYB TFs and bHLH TFs and PpUFGT in tobacco leaf. Infiltration sites in upper left of leaf indicate combinations of ‘PpMYB6, PpbHLH35, and *proPpUFGT*’ (**A**), ‘PpMYB6, PpbHLH51, and *proPpUFGT*’ (**B**), and ‘PpMYB6, PpbHLH36-like, and *proPpUFGT*’ (**C**), respectively. Infiltration sites in lower left of leaf indicate combinations of ‘PpMYB44-like, PpbHLH35, and *proPpUFGT*’ (**A**), ‘PpMYB44-like, PpbHLH51, and *proPpUFGT*’ (**B**), and ‘PpMYB44-like, PpbHLH36-like, and *proPpUFGT*’ (**C**), respectively. A combination of ‘GUS and *proPpUFGT*’ was used as a negative control (right side of leaf). Color changed from blue to red indicates gradually increasing luciferase activity.

A dual luciferase assay was conducted on tobacco leaves that were transiently infiltrated with mixed GV3101 suspension. PpMYB6 activated the promoter *PpUFGT* when it was co-infiltrated with PpbHLH35, PpbHLH51, and PpbHLH36-like. When PpMYB44-like was co-infiltrated with PpbHLH35, it also activated *PpUFGT*. By contrast, PpMYB44-like did not activate *PpUFGT* even when it was co-infiltrated with PpbHLH51 and PpbHLH36-like (Figure 7). These results indicated that PpMYB6, PpMYB44-like, PpbHLH35, PpbHLH51, and PpbHLH36-like upregulation induced anthocyanin accumulation in the flesh of sugar-treated peach fruit.

## 3. Discussion

Sugars promote anthocyanin accumulation in many plants. Cyanidin-3-glucoside was accumulated in *Clematis pitcheri* shoots in response to high sucrose concentrations [18]. Pelargonidin 3-glucoside, pelargonidin 3-rutinoside, pelargonidin 3-malonylglucoside, and pelargonidin 3-methylmalonyglucoside increased in postharvest strawberry fruit treated with 50 mM sucrose [21]. Sucrose, glucose, fructose, and sorbitol induced similar degrees of cyanidin-3-*O*-rutinoside and cyanidin-3-*O*-glucoside accumulation in the fruit of the red-blushed apricot cultivar [30]. Sucrose most strongly induced pelargonidin-3-*O*-glucoside accumulation in the hypocotyls and roots of red radish seedlings. By contrast, 1:1 fructose:glucose only weakly activated anthocyanin accumulation in the same crop [19]. The cyanidin-3-glucoside content increased in the mesocarp disks of blood-fleshed peach fruit treated with 100 mM sucrose [29]. The foregoing reports demonstrated that anthocyanins accumulated as glycosides in fruit pulp. In this study, the glucose, sucrose, sorbitol, and fructose treatments all increased the relative anthocyanin content of blood-fleshed peach fruit. However, the anthocyanins accumulated in the form of cyanidin-3-*O*-(6-*O*-p-coumaroyl) glucoside rather than cyanidin 3-*O*-glucoside. After analyzing the transcriptome data, we found that there were three acyltransferase genes which might be associated with the acylation of ayanidin- 3-*O*-glucoside (Tables S7 and S8). The peach cultivar, exogenous sugar concentrations, treatment times, and cultivation temperatures were similar between the present study and that of Rumainum et al. However, while we harvested our fruits before ripening, Rumainum et al. collected theirs at maturation. This sampling time discrepancy might account for the observed differences in anthocyanin accumulation between these studies.

Anthocyanin biosynthesis can be regulated by MYB and bHLH transcription factors (TFs). In Arabidopsis, AtPAP1, AtPAP2, AtMYB113, AtGL3, AtEGL3, and AtTT8 positively influenced anthocyanin biosynthesis [9,17]. In apple fruit, MdMYB10 promoted anthocyanin biosynthesis by interacting with both MdbHLH3 and MdbHLH33 [10]. In strawberry fruit, when FvMYB was co-infiltrated with FvbHLH33, it activated the FvDFR and FvUFGT promoters [16]. In peach fruit, PpMYB10.1, PpMYB10.2, PpMYB10.3, and Pp bHLH3 induced anthocyanin biosynthesis by upregulating the structural genes *PpDFR* and *PpUFGT* [25,26]. In the present study, two MYB TFs and three bHLH3 TFs were identified based on metabolome and transcriptome data. All of them were phylogenetically near *O*sMYB, PeMYB11, and GhMYC1 which upregulate anthocyanin biosynthesis [31,32,33]. Thus, they are probably anthocyanin biosynthesis activators in sugar-treated peach fruit flesh.

Arabidopsis uses different signal transduction pathways for sucrose and neutral sugars such as glucose and fructose. Glucose signaling molecules are sensed by the hexokinase HXK1 in Arabidopsis. HXK1 regulates the expression of sugar-related genes such as *CAB1* (chlorophyll *a/b*-binding protein), *PC (*plastocyanin), and *rbcS* (ribulose-1,5-bisphosphate carboxylase small subunit) [34]. Sucrose represses *ATB2/AtbZIP11* (leucine zipper (bZIP)-type transcription factor) translation via an open reading frame (*O*RF) encoding 42 amino acids. By contrast, glucose and fructose are relatively less effective in this process [35]. Sucrose also upregulates certain genes controlling anthocyanin biosynthesis whereas glucose and fructose have weak or no impact on their expression levels [36]. However, we found that glucose, sucrose, fructose, and sorbitol all had similar effects on anthocyanin accumulation. Therefore, they might activate the anthocyanin biosynthesis-specific TFs, which further contribute to anthocyanin biosynthesis in peach fruit flesh.

As signaling molecules, sugars control regulatory genes associated with anthocyanin biosynthesis [37]. In Arabidopsis seedlings, the regulatory genes AtMYB75/AtPAP1 involved in anthocyanin biosynthesis are upregulated by sucrose induction [22]. This signaling system is induced independently of hexokinase (HXK1) systems. In apple fruit, the energy sensor MdSnRK1.1 interacts with MdJAZ18, which is a repressor in the jasmonate signaling pathway. MdJAZ18 is then phosphorylated and degraded, MdbHLH3 is released, and its sucrose-induced anthocyanin accumulation activity is recovered [38]. It has been reported that MdbHLH3 can interact with the MYB transcription factors MdMYB9 and MdMYB11 to facilitate anthocyanin biosynthesis in apple [39,40]. Hence, MdMYB9 and MdMYB11 might participate in sucrose-induced anthocyanin accumulation. In the present study, the MYB transcription factors PpMYB6 and PpMYB44-like and the bHLH transcription factors PpbHLH35, PpbHLH51, and PpbHLH36-like were identified and activated the expression of the key anthocyanin structural gene *PpUFGT*. Hence, the foregoing TFs have positive influences on anthocyanin accumulation. However, the key regulatory genes *PpMYB10.1* and *PpBL* [25,26] were not upregulated. By contrast, they were highly expressed at the late stage of blood-flesh peach fruit development. They participate in anthocyanin accumulation by upregulating the important structural genes *PpDFR* and *PpUFGT*. Thus, we propose that the regulatory mechanism of anthocyanin accumulation differs between fruits subjected to exogenous sugar treatment in vitro and those that naturally ripen on a tree.

**Figure 8:**
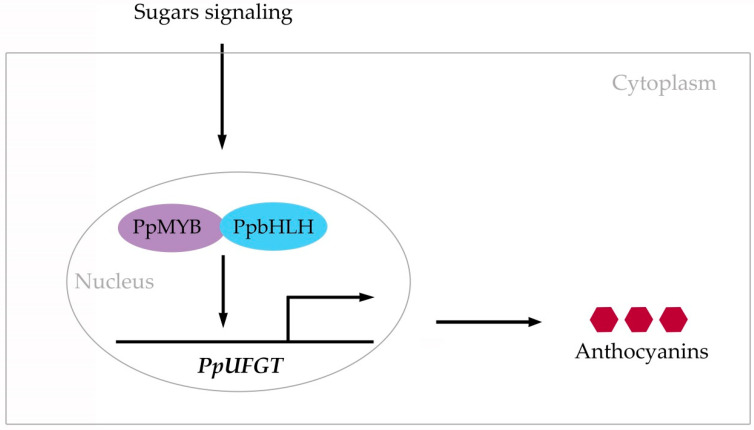
Model of two MYB and three bHLH3 TFs activating anthocyanin accumulation in flesh of peach fruit treated with four different sugars in vitro. Black arrow pointing to PpMYB and PpbHLH indicates that sugar signaling had a positive impact on their biosynthesis. Black arrow pointing to *PpUFGT* indicates that PpMYB and PpbHLH bind *PpUFGT* promoter. Folded line with arrow indicates that PpMYB and PpbHLH upregulate *PpUFGT*. Black arrow pointing to anthocyanins indicates that PpUFGT induces their biosynthesis. Sugar signaling was mediated by glucose, sucrose, sorbitol, and fructose. PpMYB includes PpMYB6 and PpMYB44-like. PpbHLH includes PpbHLH35, PpbHLH51, and PpbHLH36-like.

Based on the foregoing findings, we propose a model for anthocyanin accumulation in the flesh of peach fruit treated with glucose, sucrose, sorbitol, and fructose in vitro (Figure 8).

## 4. Materials and Methods

### 4.1. Plant Materials

The 14-year-old blood-fleshed peach cultivar ‘Tianjin Shui Mi’ was used in this study. It is an ancient Chinese landrace and contains abundant of anthocyanin compared with other cultivars. The anthocyanin content of this landrace is about 50 times that of other main cultivars. The trees were normally cultivated and managed. Ten fruits distributed along the outer crown of each tree were promptly picked at 80 d post-anthesis to measure their anthocyanin content in response to sugar treatment.

### 4.2. Metabolite Production of Fruit Flesh Induced by Sugars In Vitro

The fruit flesh was cut into small pieces (2 mm × 2 mm × 2 mm) and incubated at room temperature (25 °C) in 2-(4-morpholino)ethanesulfonic acid (MES) culture medium (pH 6.5) containing various sugars (glucose, sucrose, fructose, or sorbitol) (Solarbio Beijing China). The control was sugar-free MES medium. The MES medium consisted of 100 mM MES (pH 5.5), 5 mM CaCl2, 1 mM EDTA, 10 mM vitamin C (ascorbic acid), and 100 mM glucose, sucrose, fructose, or sorbitol. After 12 h and 24 h, peach fruit flesh was immersed in liquid nitrogen and stored at −80 °C.

### 4.3. Metabolomics Analysis

Three replicates of the flesh from sugar-treated and the control peach fruit were used in the metabolomics analysis. Twenty-five milligrams of peach fruit flesh were placed in a 1.5 mL centrifuge tube (Eppendorf GmbH, Hamburg, Germany) containing 800 μL of an aqueous methanol solution and pulverized in TissueLyser (QIAGEN Shanghai China) with a steel ball at 55 Hz for 4 min. The powder was then centrifuged at 30,000× *g* for 20 min and the supernatant was transferred to a new EP tube. The EP tube was placed in the LC-MS (liquid chromatograph-mass spectrometer) system (ACQUITY UPLC BEH C18; 100 mm × 2.1 mm; 1.7 mm; Waters Corp., Milford, MA, USA) for reversed-phase separation. The column oven temperature was kept constant at 50 °C, the flow rate was 0.4 mL/min, and the mobile phase consisted of solvent A (water + 0.1% (*v*/*v*) formic acid) and solvent B (acetonitrile + 0.1% (*v*/*v*) formic acid). The gradient elution conditions were 100% phase A, 0–2 min; 0–100% phase B, 2–11 min; 100% phase B, 11–13 min; and 0–100% phase A, 13–15 min. The sample injection volume was 10 μL [41].

The metabolites eluted from the column were detected by mass spectrometry (Xevo G2 XS QT*O*F; Waters Corp.). The Q-T*O*F (quadrupole time-of-flight) was used in both positive and negative ion modes. The cone voltages were set to 3 kV–40 V and 1 kV–40 V for the positive and negative ion modes, respectively. The T*O*F mass arrangement was 50–1200 Da and the scan time was 0.2 s. For MS/MS detection, all precursors were fragmented using 20–40 eV and the scan time was set to 0.2 s. The MS data were acquired in centroid MSE mode and identified according to the Kyoto Encyclopedia of Genes and Genomes (KEGG) database. Metabolites differentially expressed between the sugar treatments and the control were selected based on the parameters fold-change (sugar treatment/control) > 1.2 or < 0.8333 [42].

### 4.4. RNA Sequencing

Total RNA was extracted with a kit (Waryong, Beijing, China), treated with RNase-free DNAase (Takara, Dalian, China), and reverse-transcribed with a Supremo III RT kit (BioTeKe, Beijing, China). Total RNA concentration and purity were assessed with an Agilent Bioanalyzer 2100 Bioanalyzer (Agilent Technologies, Santa Clara, CA, USA) and NanoDrop 2000 (Thermo, Waltham, MA, USA), respectively.

Magnetic beads with *O*ligo (dT)s were used to enrich mRNA from 5 mg total RNA. The mRNA was randomly fragmented with fragmentation buffer and first-strand cDNA was synthesized with random hexamers. Double-stranded cDNA was then synthesized with dNTPs, RNase H, and DNA polymerase I. The double-stranded cDNA was enriched by adding poly-(A)s and PCR amplification. The enriched cDNA was linked to a vector, which was used to construct sequencing library, and analyzed in an Agilent Bioanalyzer 2100 system (Agilent Technologies). The cDNA library sequencing was performed in a HiSeq 2500 system (Illumina, San Diego, CA, USA). All peach fruit flesh samples were sequenced in three biological replicates. The low-quality reads were removed and the high-quality data were aligned to the peach reference genome (Lovell 2.0) with TopHat2 using its default parameters [43]. The gene expression levels were calculated as fragments per kilobase per million reads (FPKM). Gene ontology (G*O*) annotations were analyzed according to Blast2G*O* [44] and WEG*O* [45]. Differentially expressed upregulated genes were selected according to the criteria of sugar treatment FPKM > 1 and fold change (sugar treatment/control) > 2.0. The upregulated transcription factors (TFs) were selected for the samples of flesh of peach fruit exposed to the sugars for 12 h. The TFs common to all four data types were screened. For the samples of peach fruit flesh exposed to the sugars for 24 h, the upregulated TFs were selected in the same manner as those identified for the samples of peach fruit flesh exposed to the sugars for 12 h. The upregulated TFs common to both foregoing treatments were selected for the subsequent analysis.

### 4.5. Dual-Luciferase Tobacco Leaf Assay

The promoter sequence of *Pp.UFGT* from ‘Tianjin Shui Mi’ was synthesized at Beijing Liuhe Bgi Co. Ltd. (Beijing, China) and infused into pGreenII 0800LUC vector [26,46]. The sequence of promoter of *PpUFGT* was taken from GDR database (Available online: https://www.rosaceae.org (accessed on 12 July 2021)). The coding sequences (CDS) of *PpMYB6*, *PpMYB44-like*, *PpbHLH35*, *PpbHLH51*, and *PpbHLH36-like* selected from the transcriptome analysis were also synthesized at Beijing Liuhe Bgi Co. Ltd. and inserted into the pBI121 vector under the control of the 35 s promoter. The *GUS* gene was infused into the pBI121 vector as a negative control [26]. All recombinant vectors were transformed into A. tumefaciens GV3101 and incubated at 28 °C for 2 d. Individual transformants were resuspended in 1.0 mL Luria-Bertani (LB) medium (Solarbio Beijing China) containing 50 mg/mL kanamycin for 10 h. Ten microliters were placed in 15 mL LB medium containing 50 mg/mL kanamycin and shaken at 28 °C for 8–12 h. After centrifuging and removing the medium, we adjusted the agrobacteria *O*D to 0.4–0.6 with infiltration buffer comprising 0.5 M MES, 1.0 mM MgCl_2_, and 1.0 μM acetosyringone. The suspension was injected into three young leaves per tobacco (*Nicotiana benthamiana*) plant. The leaves injected with agrobacteria were excised after 2.5 d, immersed in *D*-luciferin sodium salt solution for 10 min, and placed in a multifunctional imaging analysis system (Tanon, Shanghai, China) to measure luminosity.

## 5. Conclusions

‘PpMYB6 and PpbHLH35′, ‘PpMYB6 and PpbHLH51′, ‘PpMYB6 and PpbHLH36-like’, and ‘PpMYB44-like and PpbHLH35′ might participate in anthocyanin accumulation via up-regulating key structural gene (*PpUFGT*) in the flesh of peach fruit treated with exogenous glucose, sucrose, sorbitol, and fructose in vitro.

## Data Availability

Data will be available on reasonable request.

## Supplementary Materials

The following are available online at https://www.mdpi.com/article/10.3390/plants11040507/s1. The metabolome and transcriptome analysis original data were deposited in the Figshare database (Available online: https://doi.org/10.6084/m9.figshare.15071700.v1 (accessed on 15 September 2021)). Figure S1: Pathway enrichment analysis of top 100 upregulated metabolites in flesh of peach fruit treated with glucose, sucrose, sorbitol, and fructose for 12 h, Figure S2: Pathway enrichment analysis of top 100 downregulated metabolites in flesh of peach fruit treated with glucose, sucrose, sorbitol, and fructose for 12 h, Figure S3: Pathway enrichment analysis of top 100 upregulated metabolites in flesh of peach fruit treated with glucose, sucrose, sorbitol, and fructose for 24 h, Figure S4: Pathway enrichment analysis of top 100 downregulated metabolites in flesh of peach fruit treated with glucose, sucrose, sorbitol, and fructose for 24 h, Table S1: Metabolites generated through LC-MC system in flesh of peach fruit treated with glucose, sucrose, sorbitol, and fructose for 12 h, Table S2: Metabolites generated through LC-MC system in flesh of peach fruit treated with glucose, sucrose, sorbitol, and fructose for 24 h, Table S3: Flavonoids in the anthocyanin pathway, which were identified in flesh of peach fruit treated with glucose, sucrose, sorbitol, and fructose for 12 h, Table S4: Flavonoids in the anthocyanin pathway, which were identified in flesh of peach fruit treated with glucose, sucrose, sorbitol, and fructose for 24 h. Table S5: Relative expression (FPKM) of major anthocyanin biosynthesis and regulatory genes in flesh of sugar-treated and untreated peach fruit after 12 h, Table S6: Relative expression (FPKM) of major anthocyanin biosynthesis and regulatory genes in flesh of sugar-treated and untreated peach fruit after 24 h, Table S7: Relative expression (FPKM) of acyltransferase genes which might be associated with the acylation of cyanidin 3-O-glucoside in sugar treated flesh for 12 h, Table S8: Relative expression (FPKM) of acyltransferase genes which might be associated with the acylation of cyanidin 3-O-glucoside in sugar treated flesh for 24 h, Tables S9–S24: Top 100 up or downregulated metabolites in flesh of peach fruit treated with glucose, sucrose, sorbitol, and fructose for 12 and 24 h, respectively.
