# Supplementary material for: Two MYB and Three bHLH Family Genes Participate in Anthocyanin Accumulation in the Flesh of Peach Fruit Treated with Glucose, Sucrose, Sorbitol, and Fructose In Vitro
Source: Plants (Basel). 2022 Feb 13;11(4):507. doi: 10.3390/plants11040507 (PMC8879194; doi:10.3390/plants11040507)
Supplement: Supplementary file 1 [file plants-11-00507-s001.zip › plants-1507324-SI.pdf]

Supplementary data

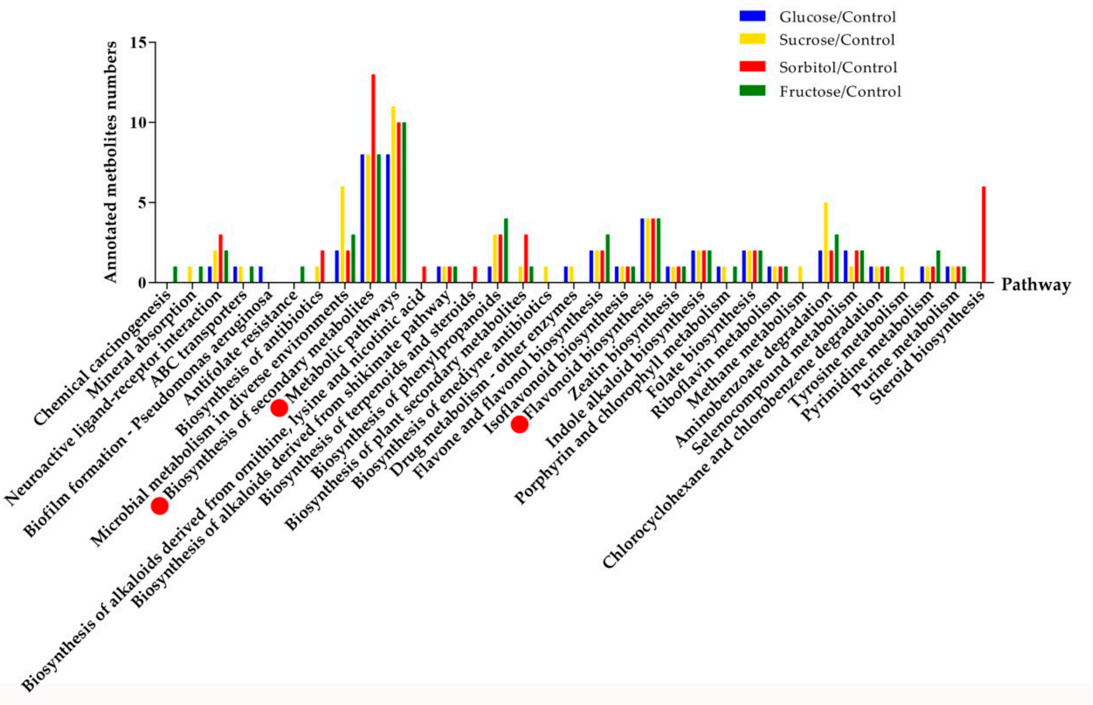

Figure S1. Pathway enrichment analysis of top 100 upregulated metabolites in flesh of peach fruit treated with glucose, sucrose, sorbitol, and fructose for 12 h.

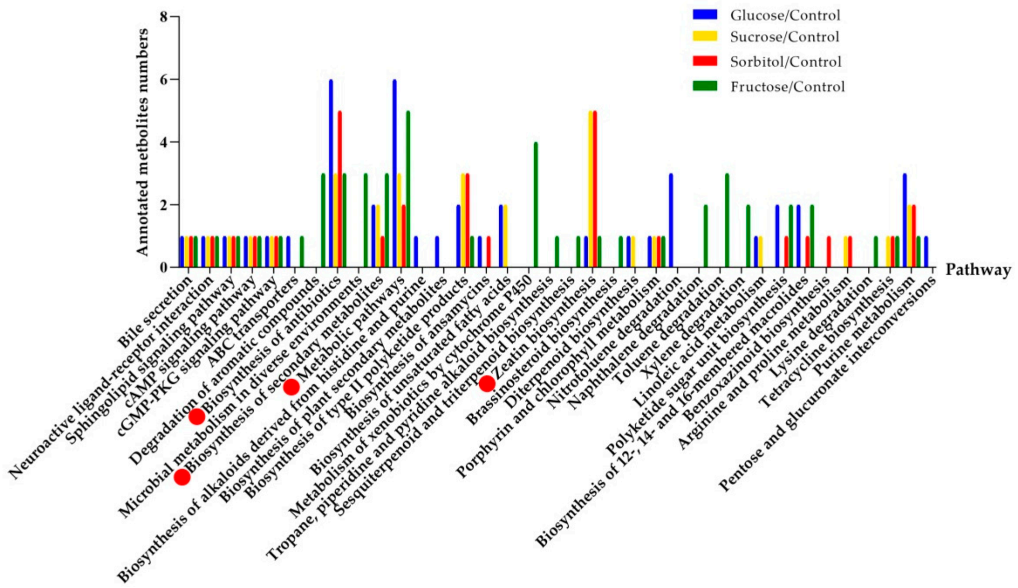

Figure S2. Pathway enrichment analysis of top 100 downregulated metabolites in flesh of peach fruit treated with glucose, sucrose, sorbitol, and fructose for 12 h.

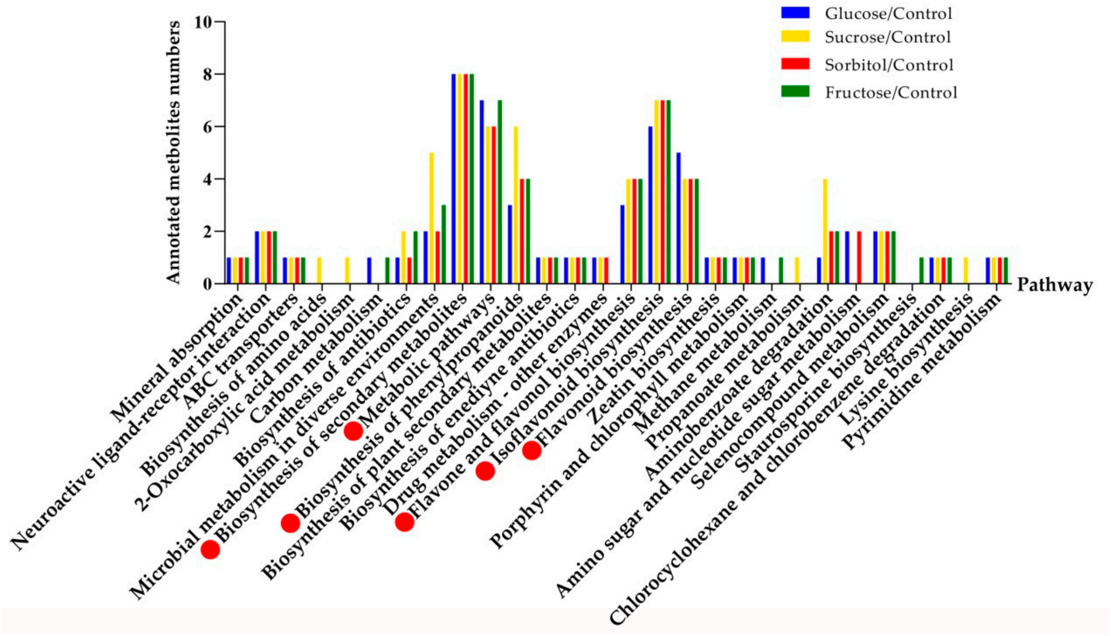

Figure S3. Pathway enrichment analysis of top 100 upregulated metabolites in flesh of peach fruit treated with glucose, sucrose, sorbitol, and fructose for 24 h.

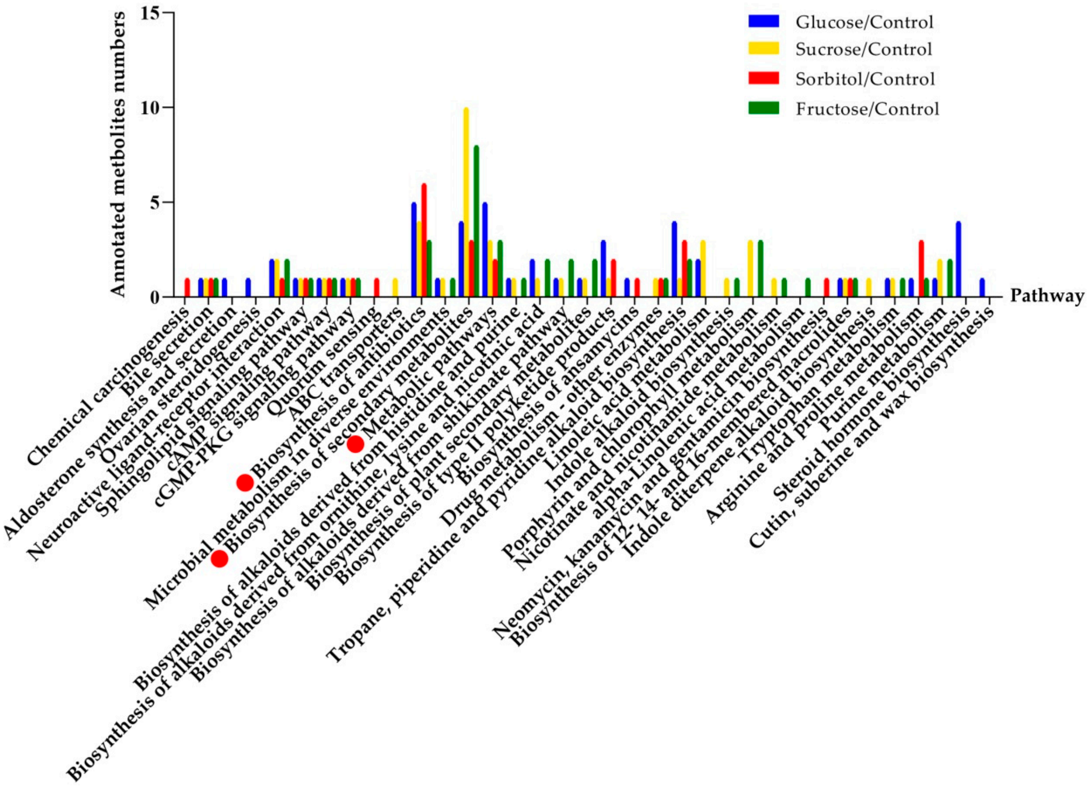

Figure S4. Pathway enrichment analysis of top 100 downregulated metabolites in flesh of peach fruit treated with glucose, sucrose, sorbitol, and fructose for 24 h.

Table S1. Metabolites generated through LC-MC system in flesh of peach fruit treated with glucose, sucrose, sorbitol, and fructose for 12 h.

Sugar treatment	NO. of up-regulated metabolites	NO. of down-regulated metabolites	Total metabolites
Glucose	3923	7537	24228
Sucrose	3091	7699	24228
Sorbitol	4038	8067	24228
Fructose	3938	7807	24228

Table S2. Metabolites generated through LC-MC system in flesh of peach fruit treated with glucose, sucrose, sorbitol, and fructose for 24 h.

Sugar treatment	NO. of up-regulated metabolites	NO. of down-regulated metabolites	Total metabolites
Glucose	5023	7602	24228
Sucrose	5062	7444	24228
Sorbitol	4952	7734	24228
Fructose	5490	7870	24228

Table S3. Flavonoids in the anthocyanin pathway, which were identified in flesh of peach fruit treated with glucose, sucrose, sorbitol, and fructose for 12 h.

**Metabolites name**	**Control**		**Glucose**		**Sucrose**		**Sorbitol**		**Fructose**	
	**M/Z**	**Log10(M/Z)**	**M/Z**	**Log10(M/Z)**	**M/Z**	**Log10(M/Z)**	**M/Z**	**Log10(M/Z)**	**M/Z**	**Log10(M/Z)**
Phenylalanine	386226.22	5.59	368522.83	5.57	350739.45	5.54	344637.95	5.53	393582.88	5.59
Cinnamic acid	386226.22	5.59	368522.83	5.57	350739.45	5.54	344637.95	5.53	393582.88	5.59
p-coumaric acid	15127.69	4.18	14033.61	4.15	12804.68	4.11	12387.02	4.09	13556.95	4.13
p-Coumaroyl-CoA	213082.96	5.33	121055.66	5.08	129531.74	5.11	114399.53	5.06	95672.16	4.98
Chalcone	49573.47	4.70	20616.98	4.31	23523.52	4.37	23094.19	4.36	18729.57	4.27
Naringenin	9813.51	3.99	8017.86	3.90	8981.31	3.95	7873.49	3.90	8896.63	3.95
Dihydroquercetin	8724.95	3.94	33113.04	4.52	16365.36	4.21	17046.10	4.23	27083.94	4.43
Leucocyanidin	295031.04	5.47	309873.88	5.49	323879.02	5.51	318564.19	5.50	334369.45	5.52
Catechin	9813.51	3.99	8017.86	3.90	8981.31	3.95	7873.49	3.90	8896.63	3.95
Cyanidin 3-O-glucoside	9387.09	3.97	5984.24	3.78	5990.22	3.77	6339.39	3.80	5991.54	3.78
Cyanidin 3-O-(6-O-p-coumaroyl)glucoside	21156.36	4.33	38828.24	4.59	31759.99	4.50	40219.23	4.60	45262.39	4.66

Note: M/Z indicates metabolites content. The same as below.

Table S4. Flavonoids in the anthocyanin pathway, which were identified in flesh of peach fruit treated with glucose, sucrose, sorbitol, and fructose for 24 h

**Metabolites name**	**Control**		**Glucose**		**Sucrose**		**Sorbitol**		**Fructose**	
	**M/Z**	**Log10(M/Z)**	**M/Z**	**Log10(M/Z)**	**M/Z**	**Log10(M/Z)**	**M/Z**	**Log10(M/Z)**	**M/Z**	**Log10(M/Z)**
Phenylalanine	314828.00	5.49	308671.54	5.49	320431.33	5.50	333346.86	5.52	335008.33	5.53
Cinnamic acid	314828.00	5.49	308671.54	5.49	320431.33	5.50	333346.86	5.52	335008.33	5.53
p-coumaric acid	12613.06	4.10	12026.26	4.08	12416.39	4.09	12943.34	4.11	13039.00	4.12
p-Coumaroyl-CoA	263150.61	5.42	69945.45	4.84	123895.40	5.09	75642.53	4.88	71672.25	4.86
Chalcone	32458.65	4.51	9567.85	3.97	11588.22	4.06	11131.24	4.05	12237.22	4.09
Naringenin	8421.02	3.93	9704.57	3.98	10184.38	4.00	8454.94	3.93	8254.06	3.92
Dihydroquercetin	8100.37	3.91	57464.86	4.75	112697.89	5.05	60378.40	4.78	74462.59	4.87
Leucocyanidin	220098.87	5.34	261014.67	5.42	303594.46	5.48	301168.21	5.47	317407.54	5.50
Catechin	8421.02	3.93	9704.57	3.98	10184.38	4.00	8454.94	3.93	8254.06	3.92
Cyanidin 3-O-glucoside	8299.80	3.92	4686.30	3.67	6061.02	3.78	5557.84	3.74	4639.18	3.66
Cyanidin 3-O-(6-O-p-coumaroyl)glucoside	12715.20	4.10	31454.31	4.50	31050.83	4.49	22174.19	4.35	38254.87	4.58

Table S5. Relative expression (FPKM) of major anthocyanin biosynthesis and regulatory genes in flesh of sugar-treated and untreated peach fruit after 12 h

Gene	Control	glucose	sucrose	sorbitol	fructose
PpDFR	7.43±0.34**d**	65.80±9.64**b**	29.03±3.54**c**	33.01±10.50**c**	91.52±27.63**a**
PpUFGT	0.61±0.03**b**	67.10±11.19**a**	68.08±7.13**a**	70.87±10.18**a**	54.79±4.22**a**
PpMYB10.1	1.59±0.17**a**	0.62±0.31**b**	0.72±0.19**b**	0.36±0.01**b**	0.42±0.09**b**
PpBL	142.8±4.12**a**	1.46±0.29**b**	1.25±0.30**b**	1.10±0.41**b**	2.01±0.59**b**

Table S6. Relative expression (FPKM) of major anthocyanin biosynthesis and regulatory genes in flesh of sugar-treated and untreated peach fruit after 24 h

Gene	Control	glucose	sucrose	sorbitol	fructose
PpDFR	15.45±1.81**c**	147.13±28.89**b**	125.54±15.57**b**	132.92±11.67**b**	301.00±58.58**a**
PpUFGT	1.24±1.38**c**	30.37±0.29**ab**	24.48±1.71**b**	25.08±5.60**b**	41.91±8.95**a**
PpMYB10.1	1.30±0.19**a**	0.11±0.05**b**	0.07±0.02**b**	0.045±0.015**b**	0.16±0.05**b**
PpBL	144.79±1.90**a**	2.34±0.60**b**	2.38±0.10**b**	2.49±0.54**b**	3.28±0.15**b**

Table S7. Relative expression (FPKM) of acyltransferase genes which might be associated with the acylation of cyanidin 3-O-glucoside in sugar treated flesh for 12 h.

Gene id	Control	glucose	sucrose	sorbitol	fructose
18787370	6.16	16.78	14.23	14.16	18.98
18767482	5.15	17.36	16.50	15.62	15.42
109946873	1.96	11.37	14.93	14.93	8.71

Table S8. Relative expression (FPKM) of acyltransferase genes which might be associated with the acylation of cyanidin 3-O-glucoside in sugar treated flesh for 24 h

Gene id	Control	glucose	sucrose	sorbitol	fructose
18767482	3.54	9.86	9.25	8.08	12.82
109946873	1.43	4.23	3.87	4.11	4.57

Table S9. The top 100 upregulated metabolites in flesh of peach fruit treated with glucose for 12 h.

Compound ID	fold-change	sample	Pathway
3.87_719.0752m/z	366.7202907	H121:H120	NULL
4.45_841.4009m/z	271.999233	H121:H120	NA
3.87_385.9937n	133.5601414	H121:H120	map00450 Selenocompound metabolism
8.69_701.2027m/z	114.1470934	H121:H120	NA
3.41_523.1083m/z	107.3829462	H121:H120	NA
0.69_390.1136n	71.26017949	H121:H120	NULL
1.17_331.1182m/z	55.53273234	H121:H120	NULL
1.17_331.1182m/z	55.53273234	H121:H120	NULL
1.17_331.1182m/z	55.53273234	H121:H120	NULL
1.17_331.1182m/z	55.53273234	H121:H120	NULL
1.17_331.1182m/z	55.53273234	H121:H120	NULL
1.17_331.1182m/z	55.53273234	H121:H120	map00901 Indole alkaloid biosynthesis; map01110 Biosynthesis of secondary metabolites
1.17_331.1182m/z	55.53273234	H121:H120	map00901 Indole alkaloid biosynthesis; map01110 Biosynthesis of secondary metabolites
1.17_331.1182m/z	55.53273234	H121:H120	NULL
1.17_331.1182m/z	55.53273234	H121:H120	NULL
1.17_331.1182m/z	55.53273234	H121:H120	NULL
1.17_331.1182m/z	55.53273234	H121:H120	NULL
1.17_331.1182m/z	55.53273234	H121:H120	map01063 Biosynthesis of alkaloids derived from shikimate pathway
1.17_331.1182m/z	55.53273234	H121:H120	NULL
1.17_331.1182m/z	55.53273234	H121:H120	NULL
1.17_331.1182m/z	55.53273234	H121:H120	NULL
2.40_231.0839m/z	50.66330818	H121:H120	NA
3.87_170.0218n	45.23387506	H121:H120	map00627 Aminobenzoate degradation; map01061 Biosynthesis of phenylpropanoids; map01120

			Microbial metabolism in diverse environments
3.42_387.0007m/z	44.84252309	H121:H120	map00240 Pyrimidine metabolism; map00908 Zeatin biosynthesis; map01100 Metabolic pathways; map04080 Neuroactive ligand-receptor interaction
3.42_387.0007m/z	44.84252309	H121:H120	map00450 Selenocompound metabolism
4.49_541.0254m/z	42.84414111	H121:H120	map00230 Purine metabolism; map00740 Riboflavin metabolism; map00790 Folate biosynthesis; map01100 Metabolic pathways; map01110 Biosynthesis of secondary metabolites; map02025 Biofilm formation - Pseudomonas aeruginosa; map03013 RNA transport; map04014 Ras signaling pathway; map04015 Rap1 signaling pathway; map04122 Sulfur relay system; map04144 Endocytosis; map05111 Biofilm formation - Vibrio cholerae
4.49_541.0254m/z	42.84414111	H121:H120	NULL
4.49_541.0254m/z	42.84414111	H121:H120	NULL
4.49_541.0254m/z	42.84414111	H121:H120	map00790 Folate biosynthesis; map01100 Metabolic pathways
3.41_522.1011n	41.72500266	H121:H120	NA
0.72_328.1178m/z	36.9899215	H121:H120	NULL
0.72_328.1178m/z	36.9899215	H121:H120	NULL
0.72_328.1178m/z	36.9899215	H121:H120	NULL
0.72_328.1178m/z	36.9899215	H121:H120	NULL
2.37_287.0547m/z	36.47583212	H121:H120	map00941 Flavonoid biosynthesis; map00944 Flavone and flavonol biosynthesis; map01100 Metabolic pathways; map01110 Biosynthesis of secondary metabolites
2.37_287.0547m/z	36.47583212	H121:H120	map00941 Flavonoid biosynthesis; map01061 Biosynthesis of phenylpropanoids; map01100 Metabolic pathways; map01110 Biosynthesis of secondary metabolites
2.37_287.0547m/z	36.47583212	H121:H120	map00941 Flavonoid biosynthesis; map00944 Flavone and flavonol biosynthesis; map01061 Biosynthesis of phenylpropanoids; map01100 Metabolic pathways; map01110 Biosynthesis of secondary metabolites
2.37_287.0547m/z	36.47583212	H121:H120	map00941 Flavonoid biosynthesis; map01110 Biosynthesis of secondary metabolites

2.37_287.0547m/z	36.47583212	H121:H120	NULL
2.37_287.0547m/z	36.47583212	H121:H120	NULL
2.37_287.0547m/z	36.47583212	H121:H120	NULL
2.37_287.0547m/z	36.47583212	H121:H120	NULL
2.37_287.0547m/z	36.47583212	H121:H120	NULL
2.37_287.0547m/z	36.47583212	H121:H120	NULL
2.37_287.0547m/z	36.47583212	H121:H120	NULL
2.37_287.0547m/z	36.47583212	H121:H120	map00943 Isoflavonoid biosynthesis
2.37_287.0547m/z	36.47583212	H121:H120	NULL
2.37_287.0547m/z	36.47583212	H121:H120	NULL
2.37_287.0547m/z	36.47583212	H121:H120	NULL
0.77_105.9542m/z	34.26734699	H121:H120	NA
2.42_309.0266m/z	33.34307483	H121:H120	map00983 Drug metabolism - other enzymes
2.42_309.0266m/z	33.34307483	H121:H120	NULL
2.42_309.0266m/z	33.34307483	H121:H120	NULL
2.42_309.0266m/z	33.34307483	H121:H120	NULL
2.42_309.0266m/z	33.34307483	H121:H120	NULL
4.28_660.1703m/z	31.41085521	H121:H120	NA
3.44_385.9934m/z	30.61556197	H121:H120	NA
1.12_325.1426m/z	29.47665946	H121:H120	NULL
1.12_325.1426m/z	29.47665946	H121:H120	NULL
1.12_325.1426m/z	29.47665946	H121:H120	NULL
1.12_325.1426m/z	29.47665946	H121:H120	NULL
1.12_325.1426m/z	29.47665946	H121:H120	NULL
1.12_325.1426m/z	29.47665946	H121:H120	NULL

1.12_325.1426m/z	29.47665946	H121:H120	NULL
1.12_325.1426m/z	29.47665946	H121:H120	NULL
1.12_325.1426m/z	29.47665946	H121:H120	NULL
1.12_325.1426m/z	29.47665946	H121:H120	NULL
1.12_325.1426m/z	29.47665946	H121:H120	NULL
1.12_325.1426m/z	29.47665946	H121:H120	NULL
1.12_325.1426m/z	29.47665946	H121:H120	NULL
1.12_325.1426m/z	29.47665946	H121:H120	NULL
1.12_325.1426m/z	29.47665946	H121:H120	NULL
1.12_325.1426m/z	29.47665946	H121:H120	NULL
0.69_310.1202n	29.38491858	H121:H120	map04080 Neuroactive ligand-receptor interaction
0.69_310.1202n	29.38491858	H121:H120	NULL
0.69_310.1202n	29.38491858	H121:H120	NULL
0.77_388.0060m/z	28.98603116	H121:H120	NA
4.30_617.1134m/z	28.00188557	H121:H120	NA
0.75_746.1541m/z	27.63793448	H121:H120	NA
3.42_107.0128m/z	26.34768749	H121:H120	map00361 Chlorocyclohexane and chlorobenzene degradation; map00627 Aminobenzoate degradation; map01100 Metabolic pathways; map01120 Microbial metabolism in diverse environments
3.42_107.0128m/z	26.34768749	H121:H120	NULL
0.69_196.0645m/z	25.66520299	H121:H120	NULL
7.49_611.4240m/z	24.80335641	H121:H120	NULL
7.49_611.4240m/z	24.80335641	H121:H120	NULL
7.49_611.4240m/z	24.80335641	H121:H120	NULL
7.49_611.4240m/z	24.80335641	H121:H120	NULL

0.74_639.1689m/z	24.21045049	H121:H120	map00860 Porphyrin and chlorophyll metabolism; map01060 Biosynthesis of plant secondary metabolites; map01100 Metabolic pathways; map01110 Biosynthesis of secondary metabolites; map02010 ABC transporters; map04978 Mineral absorption
0.74_639.1689m/z	24.21045049	H121:H120	NULL
3.87_664.1464n	23.37647218	H121:H120	NA
7.39_474.3916m/z	22.94391001	H121:H120	NULL
7.39_474.3916m/z	22.94391001	H121:H120	NULL
7.39_474.3916m/z	22.94391001	H121:H120	NULL
7.39_474.3916m/z	22.94391001	H121:H120	NULL
7.39_474.3916m/z	22.94391001	H121:H120	NULL
7.39_474.3916m/z	22.94391001	H121:H120	NULL
3.96_660.1705m/z	22.5490517	H121:H120	NA
3.42_170.0217n	22.10979385	H121:H120	map00627 Aminobenzoate degradation; map01061 Biosynthesis of phenylpropanoids; map01120 Microbial metabolism in diverse environments
0.69_325.1480m/z	19.21103393	H121:H120	NA
5.03_279.0499m/z	18.13658857	H121:H120	NULL
5.03_279.0499m/z	18.13658857	H121:H120	NULL

Table S10. The top 100 upregulated metabolites in flesh of peach fruit treated with sucrose for 12 h.

Compound ID	fold-change	sample	Pathway
3.87_719.0752m/z	319.1707276	H122:H120	NULL
3.87_385.9937n	124.038658	H122:H120	map00450 Selenocompound metabolism
1.17_331.1182m/z	69.28819468	H122:H120	NULL
1.17_331.1182m/z	69.28819468	H122:H120	NULL
1.17_331.1182m/z	69.28819468	H122:H120	NULL
1.17_331.1182m/z	69.28819468	H122:H120	NULL
1.17_331.1182m/z	69.28819468	H122:H120	NULL
1.17_331.1182m/z	69.28819468	H122:H120	map00901 Indole alkaloid biosynthesis; map01110 Biosynthesis of secondary metabolites
1.17_331.1182m/z	69.28819468	H122:H120	map00901 Indole alkaloid biosynthesis; map01110 Biosynthesis of secondary metabolites
1.17_331.1182m/z	69.28819468	H122:H120	NULL
1.17_331.1182m/z	69.28819468	H122:H120	NULL
1.17_331.1182m/z	69.28819468	H122:H120	NULL
1.17_331.1182m/z	69.28819468	H122:H120	NULL
1.17_331.1182m/z	69.28819468	H122:H120	map01063 Biosynthesis of alkaloids derived from shikimate pathway
1.17_331.1182m/z	69.28819468	H122:H120	NULL
1.17_331.1182m/z	69.28819468	H122:H120	NULL
1.17_331.1182m/z	69.28819468	H122:H120	NULL
0.69_390.1136n	55.84670969	H122:H120	NULL
3.41_523.1083m/z	50.64425095	H122:H120	NA

4.49_541.0254m/z	47.08154833	H122:H120	map00230 Purine metabolism; map00740 Riboflavin metabolism; map00790 Folate biosynthesis; map01100 Metabolic pathways; map01110 Biosynthesis of secondary metabolites; map02025 Biofilm formation - Pseudomonas aeruginosa; map03013 RNA transport; map04014 Ras signaling pathway; map04015 Rap1 signaling pathway; map04122 Sulfur relay system; map04144 Endocytosis; map05111 Biofilm formation - Vibrio cholerae
4.49_541.0254m/z	47.08154833	H122:H120	NULL
4.49_541.0254m/z	47.08154833	H122:H120	NULL
4.49_541.0254m/z	47.08154833	H122:H120	map00790 Folate biosynthesis; map01100 Metabolic pathways
3.87_170.0218n	42.72294645	H122:H120	map00627 Aminobenzoate degradation; map01061 Biosynthesis of phenylpropanoids; map01120 Microbial metabolism in diverse environments
3.42_387.0007m/z	42.20514729	H122:H120	map00240 Pyrimidine metabolism; map00908 Zeatin biosynthesis; map01100 Metabolic pathways; map04080 Neuroactive ligand-receptor interaction
3.42_387.0007m/z	42.20514729	H122:H120	map00450 Selenocompound metabolism
4.45_841.4009m/z	42.11265714	H122:H120	NA
4.28_660.1703m/z	38.91229043	H122:H120	NA
2.40_231.0839m/z	38.45816564	H122:H120	NA
0.72_328.1178m/z	34.97982548	H122:H120	NULL
0.72_328.1178m/z	34.97982548	H122:H120	NULL
0.72_328.1178m/z	34.97982548	H122:H120	NULL
0.72_328.1178m/z	34.97982548	H122:H120	NULL
3.73_499.0655m/z	32.5926745	H122:H120	NULL
3.44_385.9934m/z	30.33442491	H122:H120	NA
4.32_973.4445m/z	30.31490509	H122:H120	NA
4.30_617.1134m/z	29.5779374	H122:H120	NA
2.42_309.0266m/z	29.41926713	H122:H120	map00983 Drug metabolism - other enzymes

2.42_309.0266m/z	29.41926713	H122:H120	NULL
2.42_309.0266m/z	29.41926713	H122:H120	NULL
2.42_309.0266m/z	29.41926713	H122:H120	NULL
2.42_309.0266m/z	29.41926713	H122:H120	NULL
3.96_660.1705m/z	25.423448	H122:H120	NA
5.43_493.0437m/z	25.3157321	H122:H120	NA
0.69_310.1202n	24.21659313	H122:H120	map04080 Neuroactive ligand-receptor interaction
0.69_310.1202n	24.21659313	H122:H120	NULL
0.69_310.1202n	24.21659313	H122:H120	NULL
3.42_107.0128m/z	23.83587226	H122:H120	map00361 Chlorocyclohexane and chlorobenzene degradation; map00627 Aminobenzoate degradation; map01100 Metabolic pathways; map01120 Microbial metabolism in diverse environments
3.42_107.0128m/z	23.83587226	H122:H120	NULL
3.87_664.1464n	21.01773587	H122:H120	NA
3.42_170.0217n	20.91023094	H122:H120	map00627 Aminobenzoate degradation; map01061 Biosynthesis of phenylpropanoids; map01120 Microbial metabolism in diverse environments
0.69_196.0645m/z	20.6724579	H122:H120	NULL
9.60_590.0483n	20.46398591	H122:H120	NA
4.49_495.0199m/z	20.17903108	H122:H120	NULL
1.12_325.1426m/z	19.26324253	H122:H120	NULL
1.12_325.1426m/z	19.26324253	H122:H120	NULL
1.12_325.1426m/z	19.26324253	H122:H120	NULL
1.12_325.1426m/z	19.26324253	H122:H120	NULL
1.12_325.1426m/z	19.26324253	H122:H120	NULL
1.12_325.1426m/z	19.26324253	H122:H120	NULL

1.12_325.1426m/z	19.26324253	H122:H120	NULL
1.12_325.1426m/z	19.26324253	H122:H120	NULL
1.12_325.1426m/z	19.26324253	H122:H120	NULL
1.12_325.1426m/z	19.26324253	H122:H120	NULL
1.12_325.1426m/z	19.26324253	H122:H120	NULL
1.12_325.1426m/z	19.26324253	H122:H120	NULL
1.12_325.1426m/z	19.26324253	H122:H120	NULL
1.12_325.1426m/z	19.26324253	H122:H120	NULL
1.12_325.1426m/z	19.26324253	H122:H120	NULL
1.12_325.1426m/z	19.26324253	H122:H120	NULL
0.69_325.1480m/z	19.02986101	H122:H120	NA
5.03_279.0499m/z	18.88279425	H122:H120	NULL
5.03_279.0499m/z	18.88279425	H122:H120	NULL
5.03_279.0499m/z	18.88279425	H122:H120	NULL
2.37_287.0547m/z	18.37340423	H122:H120	map00941 Flavonoid biosynthesis; map00944 Flavone and flavonol biosynthesis; map01100 Metabolic pathways; map01110 Biosynthesis of secondary metabolites
2.37_287.0547m/z	18.37340423	H122:H120	map00941 Flavonoid biosynthesis; map01061 Biosynthesis of phenylpropanoids; map01100 Metabolic pathways; map01110 Biosynthesis of secondary metabolites
2.37_287.0547m/z	18.37340423	H122:H120	map00941 Flavonoid biosynthesis; map00944 Flavone and flavonol biosynthesis; map01061 Biosynthesis of phenylpropanoids; map01100 Metabolic pathways; map01110 Biosynthesis of secondary metabolites
2.37_287.0547m/z	18.37340423	H122:H120	map00941 Flavonoid biosynthesis; map01110 Biosynthesis of secondary metabolites
2.37_287.0547m/z	18.37340423	H122:H120	NULL
2.37_287.0547m/z	18.37340423	H122:H120	NULL
2.37_287.0547m/z	18.37340423	H122:H120	NULL

2.37_287.0547m/z	18.37340423	H122:H120	NULL
2.37_287.0547m/z	18.37340423	H122:H120	NULL
2.37_287.0547m/z	18.37340423	H122:H120	NULL
2.37_287.0547m/z	18.37340423	H122:H120	NULL
2.37_287.0547m/z	18.37340423	H122:H120	map00943 Isoflavonoid biosynthesis
2.37_287.0547m/z	18.37340423	H122:H120	NULL
2.37_287.0547m/z	18.37340423	H122:H120	NULL
2.37_287.0547m/z	18.37340423	H122:H120	NULL
3.41_522.1011n	17.40722556	H122:H120	NA
3.90_567.0892m/z	16.99301287	H122:H120	map01059 Biosynthesis of enediyne antibiotics; map01130 Biosynthesis of antibiotics
0.74_639.1689m/z	16.69019943	H122:H120	map00860 Porphyrin and chlorophyll metabolism; map01060 Biosynthesis of plant secondary metabolites; map01100 Metabolic pathways; map01110 Biosynthesis of secondary metabolites; map02010 ABC transporters; map04978 Mineral absorption
0.74_639.1689m/z	16.69019943	H122:H120	NULL
3.87_185.0448m/z	15.43816466	H122:H120	map00350 Tyrosine metabolism; map01100 Metabolic pathways
3.87_185.0448m/z	15.43816466	H122:H120	map00627 Aminobenzoate degradation; map01120 Microbial metabolism in diverse environments
3.87_185.0448m/z	15.43816466	H122:H120	map00680 Methane metabolism; map01120 Microbial metabolism in diverse environments; map01210 2-Oxocarboxylic acid metabolism
8.62_511.3396m/z	14.96865724	H122:H120	NULL
8.62_511.3396m/z	14.96865724	H122:H120	NULL
8.62_511.3396m/z	14.96865724	H122:H120	NULL
8.62_511.3396m/z	14.96865724	H122:H120	NULL

Table S11. The top 100 upregulated metabolites in flesh of peach fruit treated with sorbitol for 12 h.

Compound ID	fold-change	sample	Pathway
3.87_719.0752m/z	308.1884798	H123:H120	NULL
3.87_385.9937n	132.281633	H123:H120	map00450 Selenocompound metabolism
4.45_841.4009m/z	88.99981137	H123:H120	NA
0.69_390.1136n	77.34061974	H123:H120	NULL
1.17_331.1182m/z	68.66507275	H123:H120	NULL
1.17_331.1182m/z	68.66507275	H123:H120	NULL
1.17_331.1182m/z	68.66507275	H123:H120	NULL
1.17_331.1182m/z	68.66507275	H123:H120	NULL
1.17_331.1182m/z	68.66507275	H123:H120	NULL
1.17_331.1182m/z	68.66507275	H123:H120	map00901 Indole alkaloid biosynthesis; map01110 Biosynthesis of secondary metabolites
1.17_331.1182m/z	68.66507275	H123:H120	map00901 Indole alkaloid biosynthesis; map01110 Biosynthesis of secondary metabolites
1.17_331.1182m/z	68.66507275	H123:H120	NULL
1.17_331.1182m/z	68.66507275	H123:H120	NULL
1.17_331.1182m/z	68.66507275	H123:H120	NULL
1.17_331.1182m/z	68.66507275	H123:H120	NULL
1.17_331.1182m/z	68.66507275	H123:H120	map01063 Biosynthesis of alkaloids derived from shikimate pathway
1.17_331.1182m/z	68.66507275	H123:H120	NULL
1.17_331.1182m/z	68.66507275	H123:H120	NULL
1.17_331.1182m/z	68.66507275	H123:H120	NULL
8.35_435.3573m/z	57.77955297	H123:H120	map00100 Steroid biosynthesis; map01100 Metabolic pathways; map01130 Biosynthesis of antibiotics
8.35_435.3573m/z	57.77955297	H123:H120	map00100 Steroid biosynthesis; map01060 Biosynthesis of plant secondary metabolites; map01062 Biosynthesis of terpenoids and steroids; map01110 Biosynthesis of secondary metabolites

8.35_435.3573m/z	57.77955297	H123:H120	map01060 Biosynthesis of plant secondary metabolites
8.35_435.3573m/z	57.77955297	H123:H120	map00100 Steroid biosynthesis; map01110 Biosynthesis of secondary metabolites
8.35_435.3573m/z	57.77955297	H123:H120	NULL
8.35_435.3573m/z	57.77955297	H123:H120	NULL
8.35_435.3573m/z	57.77955297	H123:H120	map00100 Steroid biosynthesis; map01100 Metabolic pathways; map01110 Biosynthesis of secondary metabolites
8.35_435.3573m/z	57.77955297	H123:H120	map04080 Neuroactive ligand-receptor interaction
8.35_435.3573m/z	57.77955297	H123:H120	map00100 Steroid biosynthesis; map01100 Metabolic pathways; map01110 Biosynthesis of secondary metabolites
8.35_435.3573m/z	57.77955297	H123:H120	map00100 Steroid biosynthesis; map01110 Biosynthesis of secondary metabolites
8.35_435.3573m/z	57.77955297	H123:H120	NULL
3.41_523.1083m/z	54.83775638	H123:H120	NA
4.49_541.0254m/z	54.39531406	H123:H120	map00230 Purine metabolism; map00740 Riboflavin metabolism; map00790 Folate biosynthesis; map01100 Metabolic pathways; map01110 Biosynthesis of secondary metabolites; map02025 Biofilm formation - Pseudomonas aeruginosa; map03013 RNA transport; map04014 Ras signaling pathway; map04015 Rap1 signaling pathway; map04122 Sulfur relay system; map04144 Endocytosis; map05111 Biofilm formation - Vibrio cholerae
4.49_541.0254m/z	54.39531406	H123:H120	NULL
4.49_541.0254m/z	54.39531406	H123:H120	NULL
4.49_541.0254m/z	54.39531406	H123:H120	map00790 Folate biosynthesis; map01100 Metabolic pathways
2.86_156.1385m/z	45.79525591	H123:H120	map00960 Tropane, piperidine and pyridine alkaloid biosynthesis; map01064 Biosynthesis of alkaloids derived from ornithine, lysine and nicotinic acid; map01110 Biosynthesis of secondary metabolites
2.86_156.1385m/z	45.79525591	H123:H120	NULL
2.86_156.1385m/z	45.79525591	H123:H120	NULL

2.86_156.1385m/z	45.79525591	H123:H120	map00592 alpha-Linolenic acid metabolism; map01110 Biosynthesis of secondary metabolites
8.10_418.3894m/z	45.0644699	H123:H120	NA
3.42_387.0007m/z	44.03886005	H123:H120	map00240 Pyrimidine metabolism; map00908 Zeatin biosynthesis; map01100 Metabolic pathways; map04080 Neuroactive ligand-receptor interaction
3.42_387.0007m/z	44.03886005	H123:H120	map00450 Selenocompound metabolism
3.87_170.0218n	43.6172357	H123:H120	map00627 Aminobenzoate degradation; map01061 Biosynthesis of phenylpropanoids; map01120 Microbial metabolism in diverse environments
7.88_377.2921n	42.15354772	H123:H120	NULL
7.88_377.2921n	42.15354772	H123:H120	NULL
7.65_434.3845m/z	39.40278461	H123:H120	NA
0.72_328.1178m/z	38.48477279	H123:H120	NULL
0.72_328.1178m/z	38.48477279	H123:H120	NULL
0.72_328.1178m/z	38.48477279	H123:H120	NULL
0.72_328.1178m/z	38.48477279	H123:H120	NULL
4.28_660.1703m/z	35.40786419	H123:H120	NA
4.30_617.1134m/z	32.61754532	H123:H120	NA
2.40_231.0839m/z	30.52422768	H123:H120	NA
0.69_310.1202n	29.14359733	H123:H120	map04080 Neuroactive ligand-receptor interaction
0.69_310.1202n	29.14359733	H123:H120	NULL
0.69_310.1202n	29.14359733	H123:H120	NULL
4.49_495.0199m/z	28.86273457	H123:H120	NULL
3.44_385.9934m/z	26.6568819	H123:H120	NA
9.60_590.0483n	26.60645992	H123:H120	NA
2.37_287.0547m/z	25.71857314	H123:H120	map00941 Flavonoid biosynthesis; map00944 Flavone and flavonol biosynthesis; map01100 Metabolic pathways; map01110 Biosynthesis of secondary metabolites

2.37_287.0547m/z	25.71857314	H123:H120	map00941 Flavonoid biosynthesis; map01061 Biosynthesis of phenylpropanoids; map01100 Metabolic pathways; map01110 Biosynthesis of secondary metabolites
2.37_287.0547m/z	25.71857314	H123:H120	map00941 Flavonoid biosynthesis; map00944 Flavone and flavonol biosynthesis; map01061 Biosynthesis of phenylpropanoids; map01100 Metabolic pathways; map01110 Biosynthesis of secondary metabolites
2.37_287.0547m/z	25.71857314	H123:H120	map00941 Flavonoid biosynthesis; map01110 Biosynthesis of secondary metabolites
2.37_287.0547m/z	25.71857314	H123:H120	NULL
2.37_287.0547m/z	25.71857314	H123:H120	NULL
2.37_287.0547m/z	25.71857314	H123:H120	NULL
2.37_287.0547m/z	25.71857314	H123:H120	NULL
2.37_287.0547m/z	25.71857314	H123:H120	NULL
2.37_287.0547m/z	25.71857314	H123:H120	NULL
2.37_287.0547m/z	25.71857314	H123:H120	NULL
2.37_287.0547m/z	25.71857314	H123:H120	map00943 Isoflavonoid biosynthesis
2.37_287.0547m/z	25.71857314	H123:H120	NULL
2.37_287.0547m/z	25.71857314	H123:H120	NULL
2.37_287.0547m/z	25.71857314	H123:H120	NULL
3.42_107.0128m/z	25.68382019	H123:H120	map00361 Chlorocyclohexane and chlorobenzene degradation; map00627 Aminobenzoate degradation; map01100 Metabolic pathways; map01120 Microbial metabolism in diverse environments
3.42_107.0128m/z	25.68382019	H123:H120	NULL
0.69_325.1480m/z	24.69664081	H123:H120	NA
0.69_196.0645m/z	24.2246421	H123:H120	NULL
8.35_422.3506n	23.61712578	H123:H120	NA
3.96_660.1705m/z	23.17963726	H123:H120	NA

5.03_279.0499m/z	21.73954756	H123:H120	NULL
5.03_279.0499m/z	21.73954756	H123:H120	NULL
5.03_279.0499m/z	21.73954756	H123:H120	NULL
1.12_325.1426m/z	21.36627397	H123:H120	NULL
1.12_325.1426m/z	21.36627397	H123:H120	NULL
1.12_325.1426m/z	21.36627397	H123:H120	NULL
1.12_325.1426m/z	21.36627397	H123:H120	NULL
1.12_325.1426m/z	21.36627397	H123:H120	NULL
1.12_325.1426m/z	21.36627397	H123:H120	NULL
1.12_325.1426m/z	21.36627397	H123:H120	NULL
1.12_325.1426m/z	21.36627397	H123:H120	NULL
1.12_325.1426m/z	21.36627397	H123:H120	NULL
1.12_325.1426m/z	21.36627397	H123:H120	NULL
1.12_325.1426m/z	21.36627397	H123:H120	NULL
1.12_325.1426m/z	21.36627397	H123:H120	NULL
1.12_325.1426m/z	21.36627397	H123:H120	NULL
1.12_325.1426m/z	21.36627397	H123:H120	NULL
1.12_325.1426m/z	21.36627397	H123:H120	NULL
1.12_325.1426m/z	21.36627397	H123:H120	NULL
3.42_170.0217n	21.20643739	H123:H120	map00627 Aminobenzoate degradation; map01061 Biosynthesis of phenylpropanoids; map01120 Microbial metabolism in diverse environments
2.42_309.0266m/z	21.10985991	H123:H120	map00983 Drug metabolism - other enzymes

Table S12. The top 100 upregulated metabolites in flesh of peach fruit treated with fructose for 12 h.

Compound ID	fold-change	sample	Pathway
3.87_719.0752m/z	287.7802765	H124:H120	NULL
3.87_385.9937n	123.3953596	H124:H120	map00450 Selenocompound metabolism
0.69_390.1136n	111.7141572	H124:H120	NULL
3.41_523.1083m/z	74.89391671	H124:H120	NA
1.17_331.1182m/z	66.29064619	H124:H120	NULL
1.17_331.1182m/z	66.29064619	H124:H120	NULL
1.17_331.1182m/z	66.29064619	H124:H120	NULL
1.17_331.1182m/z	66.29064619	H124:H120	NULL
1.17_331.1182m/z	66.29064619	H124:H120	NULL
1.17_331.1182m/z	66.29064619	H124:H120	map00901 Indole alkaloid biosynthesis; map01110 Biosynthesis of secondary metabolites
1.17_331.1182m/z	66.29064619	H124:H120	map00901 Indole alkaloid biosynthesis; map01110 Biosynthesis of secondary metabolites
1.17_331.1182m/z	66.29064619	H124:H120	NULL
1.17_331.1182m/z	66.29064619	H124:H120	NULL
1.17_331.1182m/z	66.29064619	H124:H120	NULL
1.17_331.1182m/z	66.29064619	H124:H120	NULL
1.17_331.1182m/z	66.29064619	H124:H120	map01063 Biosynthesis of alkaloids derived from shikimate pathway
1.17_331.1182m/z	66.29064619	H124:H120	NULL
1.17_331.1182m/z	66.29064619	H124:H120	NULL
1.17_331.1182m/z	66.29064619	H124:H120	NULL
3.65_83.0491m/z	56.99917647	H124:H120	NULL
3.65_83.0491m/z	56.99917647	H124:H120	NULL
3.65_83.0491m/z	56.99917647	H124:H120	NULL

3.65_83.0491m/z	56.99917647	H124:H120	map05204 Chemical carcinogenesis
3.65_83.0491m/z	56.99917647	H124:H120	NULL
3.65_83.0491m/z	56.99917647	H124:H120	NULL
3.65_83.0491m/z	56.99917647	H124:H120	NULL
4.28_660.1703m/z	55.68116494	H124:H120	NA
4.45_841.4009m/z	52.20209092	H124:H120	NA
0.75_296.5526m/z	43.53521262	H124:H120	NA
3.42_387.0007m/z	42.56988911	H124:H120	map00240 Pyrimidine metabolism; map00908 Zeatin biosynthesis; map01100 Metabolic pathways; map04080 Neuroactive ligand-receptor interaction
3.42_387.0007m/z	42.56988911	H124:H120	map00450 Selenocompound metabolism
0.72_328.1178m/z	42.55551897	H124:H120	NULL
0.72_328.1178m/z	42.55551897	H124:H120	NULL
0.72_328.1178m/z	42.55551897	H124:H120	NULL
0.72_328.1178m/z	42.55551897	H124:H120	NULL
3.96_660.1705m/z	41.74266901	H124:H120	NA
3.87_170.0218n	40.73111875	H124:H120	map00627 Aminobenzoate degradation; map01061 Biosynthesis of phenylpropanoids; map01120 Microbial metabolism in diverse environments
4.49_541.0254m/z	39.80708968	H124:H120	map00230 Purine metabolism; map00740 Riboflavin metabolism; map00790 Folate biosynthesis; map01100 Metabolic pathways; map01110 Biosynthesis of secondary metabolites; map02025 Biofilm formation - Pseudomonas aeruginosa; map03013 RNA transport; map04014 Ras signaling pathway; map04015 Rap1 signaling pathway; map04122 Sulfur relay system; map04144 Endocytosis; map05111 Biofilm formation - Vibrio cholerae
4.49_541.0254m/z	39.80708968	H124:H120	NULL
4.49_541.0254m/z	39.80708968	H124:H120	NULL
4.49_541.0254m/z	39.80708968	H124:H120	map00790 Folate biosynthesis; map01100 Metabolic pathways

2.37_287.0547m/z	39.54626036	H124:H120	map00941 Flavonoid biosynthesis; map00944 Flavone and flavonol biosynthesis; map01100 Metabolic pathways; map01110 Biosynthesis of secondary metabolites
2.37_287.0547m/z	39.54626036	H124:H120	map00941 Flavonoid biosynthesis; map01061 Biosynthesis of phenylpropanoids; map01100 Metabolic pathways; map01110 Biosynthesis of secondary metabolites
2.37_287.0547m/z	39.54626036	H124:H120	map00941 Flavonoid biosynthesis; map00944 Flavone and flavonol biosynthesis; map01061 Biosynthesis of phenylpropanoids; map01100 Metabolic pathways; map01110 Biosynthesis of secondary metabolites
2.37_287.0547m/z	39.54626036	H124:H120	map00941 Flavonoid biosynthesis; map01110 Biosynthesis of secondary metabolites
2.37_287.0547m/z	39.54626036	H124:H120	NULL
2.37_287.0547m/z	39.54626036	H124:H120	NULL
2.37_287.0547m/z	39.54626036	H124:H120	NULL
2.37_287.0547m/z	39.54626036	H124:H120	NULL
2.37_287.0547m/z	39.54626036	H124:H120	NULL
2.37_287.0547m/z	39.54626036	H124:H120	NULL
2.37_287.0547m/z	39.54626036	H124:H120	NULL
2.37_287.0547m/z	39.54626036	H124:H120	map00943 Isoflavonoid biosynthesis
2.37_287.0547m/z	39.54626036	H124:H120	NULL
2.37_287.0547m/z	39.54626036	H124:H120	NULL
2.37_287.0547m/z	39.54626036	H124:H120	NULL
3.88_304.0794n	38.09596002	H124:H120	NA
0.69_310.1202n	33.83314536	H124:H120	map04080 Neuroactive ligand-receptor interaction
0.69_310.1202n	33.83314536	H124:H120	NULL
0.69_310.1202n	33.83314536	H124:H120	NULL
4.30_617.1134m/z	31.8746528	H124:H120	NA
3.54_361.0210m/z	31.8347222	H124:H120	map00240 Pyrimidine metabolism; map01100 Metabolic pathways; map01523 Antifolate resistance

0.75_746.1541m/z	28.60024969	H124:H120	NA
0.69_325.1480m/z	28.39546203	H124:H120	NA
3.76_343.0117m/z	28.14913527	H124:H120	NA
4.32_973.4445m/z	27.69418847	H124:H120	NA
0.74_288.5658m/z	27.05932156	H124:H120	NA
0.74_639.1689m/z	26.92119977	H124:H120	map00860 Porphyrin and chlorophyll metabolism; map01060 Biosynthesis of plant secondary metabolites; map01100 Metabolic pathways; map01110 Biosynthesis of secondary metabolites; map02010 ABC transporters; map04978 Mineral absorption
0.74_639.1689m/z	26.92119977	H124:H120	NULL
0.69_196.0645m/z	26.84468079	H124:H120	NULL
3.41_522.1011n	26.84129218	H124:H120	NA
1.12_325.1426m/z	26.59296591	H124:H120	NULL
1.12_325.1426m/z	26.59296591	H124:H120	NULL
1.12_325.1426m/z	26.59296591	H124:H120	NULL
1.12_325.1426m/z	26.59296591	H124:H120	NULL
1.12_325.1426m/z	26.59296591	H124:H120	NULL
1.12_325.1426m/z	26.59296591	H124:H120	NULL
1.12_325.1426m/z	26.59296591	H124:H120	NULL
1.12_325.1426m/z	26.59296591	H124:H120	NULL
1.12_325.1426m/z	26.59296591	H124:H120	NULL
1.12_325.1426m/z	26.59296591	H124:H120	NULL
1.12_325.1426m/z	26.59296591	H124:H120	NULL
1.12_325.1426m/z	26.59296591	H124:H120	NULL
1.12_325.1426m/z	26.59296591	H124:H120	NULL
1.12_325.1426m/z	26.59296591	H124:H120	NULL

1.12_325.1426m/z	26.59296591	H124:H120	NULL
1.12_325.1426m/z	26.59296591	H124:H120	NULL
3.44_385.9934m/z	26.5784678	H124:H120	NA
0.77_105.9542m/z	26.548206	H124:H120	NA
4.49_495.0199m/z	22.67107462	H124:H120	NULL
3.42_107.0128m/z	22.28681621	H124:H120	map00361 Chlorocyclohexane and chlorobenzene degradation; map00627 Aminobenzoate degradation; map01100 Metabolic pathways; map01120 Microbial metabolism in diverse environments
3.42_107.0128m/z	22.28681621	H124:H120	NULL
7.49_611.4240m/z	22.04002308	H124:H120	NULL
7.49_611.4240m/z	22.04002308	H124:H120	NULL
7.49_611.4240m/z	22.04002308	H124:H120	NULL
7.49_611.4240m/z	22.04002308	H124:H120	NULL
4.49_755.1092m/z	20.63878301	H124:H120	NA
3.42_170.0217n	20.4460805	H124:H120	map00627 Aminobenzoate degradation; map01061 Biosynthesis of phenylpropanoids; map01120 Microbial metabolism in diverse environments
4.14_339.0281m/z	19.19865968	H124:H120	map00944 Flavone and flavonol biosynthesis
4.14_339.0281m/z	19.19865968	H124:H120	NULL
4.14_339.0281m/z	19.19865968	H124:H120	NULL

Table S13. The top 100 downregulated metabolites in flesh of peach fruit treated with glucose for 12 h.

Compound ID	fold-change	sample	Pathway
3.39_118.0281n	0.113002586	H121:H120	NA
6.27_705.4132m/z	0.112766711	H121:H120	NA
5.52_451.1578m/z	0.110667107	H121:H120	NULL
5.52_451.1578m/z	0.110667107	H121:H120	NULL
4.22_377.8842m/z	0.107752666	H121:H120	NA
4.66_790.9094m/z	0.102854434	H121:H120	NA
5.03_1140.2873m/z	0.101809538	H121:H120	NA
5.52_445.2208n	0.09861181	H121:H120	NULL
5.43_643.2211m/z	0.098061928	H121:H120	map01051 Biosynthesis of ansamycins; map01130 Biosynthesis of antibiotics
4.52_665.1821m/z	0.096483671	H121:H120	NULL
5.75_522.1854m/z	0.094170451	H121:H120	NULL
8.98_860.5328m/z	0.092312244	H121:H120	NA
5.62_389.0438m/z	0.091838673	H121:H120	map00230 Purine metabolism; map01060 Biosynthesis of plant secondary metabolites; map01065 Biosynthesis of alkaloids derived from histidine and purine; map01100 Metabolic pathways; map01110 Biosynthesis of secondary metabolites; map01130 Biosynthesis of antibiotics
5.62_389.0438m/z	0.091838673	H121:H120	NULL
5.62_389.0438m/z	0.091838673	H121:H120	map01057 Biosynthesis of type II polyketide products; map01130 Biosynthesis of antibiotics
5.62_389.0438m/z	0.091838673	H121:H120	map00633 Nitrotoluene degradation
5.62_389.0438m/z	0.091838673	H121:H120	map00633 Nitrotoluene degradation
5.62_389.0438m/z	0.091838673	H121:H120	map00633 Nitrotoluene degradation
4.22_565.8222m/z	0.088405236	H121:H120	NA

3.80_490.1345m/z	0.085106255	H121:H120	map01057 Biosynthesis of type II polyketide products; map01130 Biosynthesis of antibiotics
5.92_605.2010m/z	0.083343553	H121:H120	NA
5.52_444.1420n	0.082244101	H121:H120	NULL
5.52_513.1251m/z	0.075389738	H121:H120	map00908 Zeatin biosynthesis
8.86_573.2884m/z	0.075056983	H121:H120	NULL
8.86_573.2884m/z	0.075056983	H121:H120	NULL
5.52_529.1010m/z	0.073607368	H121:H120	map00040 Pentose and glucuronate interconversions; map01100 Metabolic pathways; map02010 ABC transporters
5.52_529.1010m/z	0.073607368	H121:H120	NULL
5.52_529.1010m/z	0.073607368	H121:H120	map00522 Biosynthesis of 12-, 14- and 16-membered macrolides; map00523 Polyketide sugar unit biosynthesis; map01130 Biosynthesis of antibiotics
5.52_529.1010m/z	0.073607368	H121:H120	map00522 Biosynthesis of 12-, 14- and 16-membered macrolides; map00523 Polyketide sugar unit biosynthesis; map01130 Biosynthesis of antibiotics
5.52_529.1010m/z	0.073607368	H121:H120	NULL
5.96_292.1812m/z	0.07312791	H121:H120	NA
4.36_887.4209m/z	0.072798781	H121:H120	NA
6.10_307.2661m/z	0.068992048	H121:H120	map00591 Linoleic acid metabolism; map01040 Biosynthesis of unsaturated fatty acids; map01100 Metabolic pathways
6.10_307.2661m/z	0.068992048	H121:H120	NULL
6.10_307.2661m/z	0.068992048	H121:H120	map00904 Diterpenoid biosynthesis; map01110 Biosynthesis of secondary metabolites
6.10_307.2661m/z	0.068992048	H121:H120	NULL
6.10_307.2661m/z	0.068992048	H121:H120	NULL
6.10_307.2661m/z	0.068992048	H121:H120	NULL
6.10_307.2661m/z	0.068992048	H121:H120	NULL

6.10_307.2661m/z	0.068992048	H121:H120	map01040 Biosynthesis of unsaturated fatty acids
5.75_675.2604m/z	0.06619778	H121:H120	NULL
5.75_675.2604m/z	0.06619778	H121:H120	NULL
9.35_828.5380m/z	0.063745523	H121:H120	NA
8.34_760.4398m/z	0.063237247	H121:H120	NA
8.93_205.1591m/z	0.062709568	H121:H120	NULL
8.93_205.1591m/z	0.062709568	H121:H120	NULL
8.93_205.1591m/z	0.062709568	H121:H120	NULL
4.10_296.5217m/z	0.054973337	H121:H120	NA
4.22_566.3234m/z	0.052557695	H121:H120	NULL
5.56_608.3285m/z	0.051125693	H121:H120	NA
8.87_614.3154m/z	0.04845119	H121:H120	NA
9.25_844.5341m/z	0.046346543	H121:H120	NA
8.87_408.3381n	0.045727141	H121:H120	NA
8.87_616.3161m/z	0.044268404	H121:H120	NULL
8.87_616.3161m/z	0.044268404	H121:H120	map00860 Porphyrin and chlorophyll metabolism; map01110 Biosynthesis of secondary metabolites
4.10_444.2797m/z	0.044140922	H121:H120	NA
9.20_846.5476m/z	0.041783628	H121:H120	NA
3.39_268.1040m/z	0.041615506	H121:H120	map00230 Purine metabolism; map01100 Metabolic pathways; map04022 cGMP-PKG signaling pathway; map04024 cAMP signaling pathway; map04071 Sphingolipid signaling pathway; map04080 Neuroactive ligand-receptor interaction; map04270 Vascular smooth muscle contraction; map04923 Regulation of lipolysis in adipocytes; map04924 Renin secretion; map05012 Parkinson's disease; map05032 Morphine addiction; map05034 Alcoholism

3.39_268.1040m/z	0.041615506	H121:H120	map00230 Purine metabolism; map01100 Metabolic pathways
3.39_268.1040m/z	0.041615506	H121:H120	NULL
3.39_268.1040m/z	0.041615506	H121:H120	NULL
3.39_268.1040m/z	0.041615506	H121:H120	map04976 Bile secretion
3.39_268.1040m/z	0.041615506	H121:H120	NULL
8.86_495.3436m/z	0.040380783	H121:H120	NULL
8.86_495.3436m/z	0.040380783	H121:H120	NULL
8.86_495.3436m/z	0.040380783	H121:H120	NULL
8.86_495.3436m/z	0.040380783	H121:H120	NULL
8.86_495.3436m/z	0.040380783	H121:H120	NULL
8.86_495.3436m/z	0.040380783	H121:H120	NULL
8.86_495.3436m/z	0.040380783	H121:H120	NULL
8.86_495.3436m/z	0.040380783	H121:H120	NULL
8.86_495.3436m/z	0.040380783	H121:H120	NULL
5.71_479.1200m/z	0.038572451	H121:H120	NULL
5.71_479.1200m/z	0.038572451	H121:H120	map00253 Tetracycline biosynthesis; map01057 Biosynthesis of type II polyketide products; map01130 Biosynthesis of antibiotics
5.71_479.1200m/z	0.038572451	H121:H120	NULL
5.71_479.1200m/z	0.038572451	H121:H120	map00980 Metabolism of xenobiotics by cytochrome P450
5.71_479.1200m/z	0.038572451	H121:H120	map00980 Metabolism of xenobiotics by cytochrome P450
5.71_479.1200m/z	0.038572451	H121:H120	map00980 Metabolism of xenobiotics by cytochrome P450
5.71_479.1200m/z	0.038572451	H121:H120	map00980 Metabolism of xenobiotics by cytochrome P450
4.54_965.1975m/z	0.032941978	H121:H120	NA
4.67_898.9470m/z	0.03076624	H121:H120	NA
8.86_490.3861m/z	0.02945195	H121:H120	NULL

8.86_490.3861m/z	0.02945195	H121:H120	NULL
8.86_490.3861m/z	0.02945195	H121:H120	NULL
8.86_490.3861m/z	0.02945195	H121:H120	NULL
8.86_490.3861m/z	0.02945195	H121:H120	NULL
8.86_490.3861m/z	0.02945195	H121:H120	NULL
8.86_490.3861m/z	0.02945195	H121:H120	NULL
4.54_965.4481m/z	0.023788885	H121:H120	NULL
4.54_965.4481m/z	0.023788885	H121:H120	NULL
7.39_711.2601m/z	0.021613987	H121:H120	NA
8.19_457.3706m/z	0.021215485	H121:H120	NULL
8.19_457.3706m/z	0.021215485	H121:H120	NULL
8.19_457.3706m/z	0.021215485	H121:H120	NULL
8.19_457.3706m/z	0.021215485	H121:H120	NULL
8.19_457.3706m/z	0.021215485	H121:H120	NULL
8.19_457.3706m/z	0.021215485	H121:H120	NULL
8.19_457.3706m/z	0.021215485	H121:H120	NULL
8.19_457.3706m/z	0.021215485	H121:H120	NULL
8.19_457.3706m/z	0.021215485	H121:H120	NULL
8.87_699.5813n	0.006638315	H121:H120	NA

Table S14. The top 100 downregulated metabolites in flesh of peach fruit treated with sucrose for 12 h.

Compound ID	fold-change	sample	Pathway
5.71_317.0659m/z	0.123877106	H122:H120	NULL
5.71_317.0659m/z	0.123877106	H122:H120	NULL
5.52_451.1578m/z	0.122416736	H122:H120	NULL
5.52_451.1578m/z	0.122416736	H122:H120	NULL
4.42_770.3773m/z	0.120778407	H122:H120	NA
4.22_565.8222m/z	0.120606438	H122:H120	NA
9.39_826.5214m/z	0.119866521	H122:H120	NA
3.66_490.1343m/z	0.118471431	H122:H120	map01057 Biosynthesis of type II polyketide products; map01130 Biosynthesis of antibiotics
4.22_377.5500m/z	0.114932741	H122:H120	NA
1.68_515.0697m/z	0.111605866	H122:H120	NULL
1.68_515.0697m/z	0.111605866	H122:H120	NULL
5.01_998.9740m/z	0.11145724	H122:H120	NA
3.39_118.0281n	0.109163476	H122:H120	NA
0.57_683.9164n	0.10699075	H122:H120	NA
5.46_472.1246m/z	0.105512516	H122:H120	NA
5.03_1140.2873m/z	0.100204184	H122:H120	NA
5.52_444.1420n	0.099228353	H122:H120	NULL
0.65_727.2205m/z	0.09771697	H122:H120	NULL
8.86_573.2884m/z	0.096967934	H122:H120	NULL
8.86_573.2884m/z	0.096967934	H122:H120	NULL
5.46_821.9372m/z	0.093920008	H122:H120	NA
8.98_860.5328m/z	0.091632446	H122:H120	NA
5.52_445.2208n	0.086371042	H122:H120	NULL

5.01_998.2219m/z	0.085032106	H122:H120	NA
5.75_675.2604m/z	0.082633269	H122:H120	NULL
5.75_675.2604m/z	0.082633269	H122:H120	NULL
9.35_828.5380m/z	0.080882385	H122:H120	NA
4.27_294.1897m/z	0.08023578	H122:H120	map00330 Arginine and proline metabolism
4.22_377.8842m/z	0.07902661	H122:H120	NA
8.34_760.4398m/z	0.078386431	H122:H120	NA
4.36_887.4209m/z	0.077408144	H122:H120	NA
4.42_770.8794m/z	0.07503217	H122:H120	NA
5.92_605.2010m/z	0.074882667	H122:H120	NA
9.20_846.5476m/z	0.073099152	H122:H120	NA
4.10_296.5217m/z	0.062145725	H122:H120	NA
5.96_292.1812m/z	0.061424608	H122:H120	NA
4.66_790.9094m/z	0.054465547	H122:H120	NA
4.52_665.1821m/z	0.049887982	H122:H120	NULL
3.80_490.1345m/z	0.049668547	H122:H120	map01057 Biosynthesis of type II polyketide products; map01130 Biosynthesis of antibiotics
9.25_844.5341m/z	0.049185512	H122:H120	NA
8.93_205.1591m/z	0.045893392	H122:H120	NULL
8.93_205.1591m/z	0.045893392	H122:H120	NULL
8.93_205.1591m/z	0.045893392	H122:H120	NULL
8.87_408.3381n	0.04455591	H122:H120	NA
4.10_444.2797m/z	0.038372852	H122:H120	NA
4.54_965.4481m/z	0.037925558	H122:H120	NULL
4.54_965.4481m/z	0.037925558	H122:H120	NULL
4.54_965.1975m/z	0.037697871	H122:H120	NA

8.86_490.3861m/z	0.037405655	H122:H120	NULL
8.86_490.3861m/z	0.037405655	H122:H120	NULL
8.86_490.3861m/z	0.037405655	H122:H120	NULL
8.86_490.3861m/z	0.037405655	H122:H120	NULL
8.86_490.3861m/z	0.037405655	H122:H120	NULL
8.86_490.3861m/z	0.037405655	H122:H120	NULL
8.86_490.3861m/z	0.037405655	H122:H120	NULL
8.87_614.3154m/z	0.036877267	H122:H120	NA
8.86_495.3436m/z	0.036607568	H122:H120	NULL
8.86_495.3436m/z	0.036607568	H122:H120	NULL
8.86_495.3436m/z	0.036607568	H122:H120	NULL
8.86_495.3436m/z	0.036607568	H122:H120	NULL
8.86_495.3436m/z	0.036607568	H122:H120	NULL
8.86_495.3436m/z	0.036607568	H122:H120	NULL
8.86_495.3436m/z	0.036607568	H122:H120	NULL
8.86_495.3436m/z	0.036607568	H122:H120	NULL
8.86_495.3436m/z	0.036607568	H122:H120	NULL
5.75_522.1854m/z	0.033876498	H122:H120	NULL
3.39_268.1040m/z	0.033057313	H122:H120	map00230 Purine metabolism; map01100 Metabolic pathways; map04022 cGMP-PKG signaling pathway; map04024 cAMP signaling pathway; map04071 Sphingolipid signaling pathway; map04080 Neuroactive ligand-receptor interaction; map04270 Vascular smooth muscle contraction; map04923 Regulation of lipolysis in adipocytes; map04924 Renin secretion; map05012 Parkinson's disease; map05032 Morphine addiction; map05034 Alcoholism
3.39_268.1040m/z	0.033057313	H122:H120	map00230 Purine metabolism; map01100 Metabolic pathways
3.39_268.1040m/z	0.033057313	H122:H120	NULL

3.39_268.1040m/z	0.033057313	H122:H120	NULL
3.39_268.1040m/z	0.033057313	H122:H120	map04976 Bile secretion
3.39_268.1040m/z	0.033057313	H122:H120	NULL
4.67_898.9470m/z	0.032493397	H122:H120	NA
8.87_616.3161m/z	0.028109862	H122:H120	NULL
8.87_616.3161m/z	0.028109862	H122:H120	map00860 Porphyrin and chlorophyll metabolism; map01110 Biosynthesis of secondary metabolites
6.10_307.2661m/z	0.026954161	H122:H120	map00591 Linoleic acid metabolism; map01040 Biosynthesis of unsaturated fatty acids; map01100 Metabolic pathways
6.10_307.2661m/z	0.026954161	H122:H120	NULL
6.10_307.2661m/z	0.026954161	H122:H120	map00904 Diterpenoid biosynthesis; map01110 Biosynthesis of secondary metabolites
6.10_307.2661m/z	0.026954161	H122:H120	NULL
6.10_307.2661m/z	0.026954161	H122:H120	NULL
6.10_307.2661m/z	0.026954161	H122:H120	NULL
6.10_307.2661m/z	0.026954161	H122:H120	NULL
6.10_307.2661m/z	0.026954161	H122:H120	map01040 Biosynthesis of unsaturated fatty acids
4.20_272.6750m/z	0.025146523	H122:H120	NA
5.71_479.1200m/z	0.022528102	H122:H120	NULL
5.71_479.1200m/z	0.022528102	H122:H120	map00253 Tetracycline biosynthesis; map01057 Biosynthesis of type II polyketide products; map01130 Biosynthesis of antibiotics
5.71_479.1200m/z	0.022528102	H122:H120	NULL
5.71_479.1200m/z	0.022528102	H122:H120	map00980 Metabolism of xenobiotics by cytochrome P450
5.71_479.1200m/z	0.022528102	H122:H120	map00980 Metabolism of xenobiotics by cytochrome P450
5.71_479.1200m/z	0.022528102	H122:H120	map00980 Metabolism of xenobiotics by cytochrome P450
5.71_479.1200m/z	0.022528102	H122:H120	map00980 Metabolism of xenobiotics by cytochrome P450

8.19_457.3706m/z	0.018729698	H122:H120	NULL
8.19_457.3706m/z	0.018729698	H122:H120	NULL
8.19_457.3706m/z	0.018729698	H122:H120	NULL
8.19_457.3706m/z	0.018729698	H122:H120	NULL
8.19_457.3706m/z	0.018729698	H122:H120	NULL
8.19_457.3706m/z	0.018729698	H122:H120	NULL
8.19_457.3706m/z	0.018729698	H122:H120	NULL
8.19_457.3706m/z	0.018729698	H122:H120	NULL
8.19_457.3706m/z	0.018729698	H122:H120	NULL
5.52_513.1251m/z	0.011044458	H122:H120	map00908 Zeatin biosynthesis

Table S15. The top 100 downregulated metabolites in flesh of peach fruit treated with sorbitol for 12 h.

Compound ID	fold-change	sample	Pathway
5.52_529.1010m/z	0.104557709	H123:H120	NULL
4.22_565.8222m/z	0.104157504	H123:H120	NA
5.01_998.2219m/z	0.102634284	H123:H120	NA
5.77_561.1962m/z	0.099275024	H123:H120	NULL
5.77_561.1962m/z	0.099275024	H123:H120	NULL
7.99_704.4121m/z	0.098093182	H123:H120	NA
5.46_472.1246m/z	0.096080918	H123:H120	NA
3.68_395.6992m/z	0.095637436	H123:H120	NA
3.66_490.1343m/z	0.094741122	H123:H120	map01057 Biosynthesis of type II polyketide products; map01130 Biosynthesis of antibiotics
8.20_457.3994m/z	0.092927628	H123:H120	NA
5.52_451.1578m/z	0.086486644	H123:H120	NULL
5.52_451.1578m/z	0.086486644	H123:H120	NULL
9.48_829.5456n	0.085025295	H123:H120	NA
4.27_294.1897m/z	0.084164558	H123:H120	map00330 Arginine and proline metabolism
7.39_474.3916m/z	0.082708041	H123:H120	NULL
7.39_474.3916m/z	0.082708041	H123:H120	NULL
7.39_474.3916m/z	0.082708041	H123:H120	NULL
7.39_474.3916m/z	0.082708041	H123:H120	NULL
7.39_474.3916m/z	0.082708041	H123:H120	NULL
7.39_474.3916m/z	0.082708041	H123:H120	NULL
5.03_1140.2873m/z	0.081617538	H123:H120	NA
5.52_445.2208n	0.080149502	H123:H120	NULL
4.33_630.3427m/z	0.079108616	H123:H120	NA

8.34_760.4398m/z	0.078138526	H123:H120	NA
5.96_292.1812m/z	0.070051305	H123:H120	NA
5.99_360.0899m/z	0.06989739	H123:H120	NULL
5.99_360.0899m/z	0.06989739	H123:H120	map00402 Benzoxazinoid biosynthesis
5.52_444.1420n	0.069589272	H123:H120	NULL
3.39_268.1040m/z	0.067445954	H123:H120	map00230 Purine metabolism; map01100 Metabolic pathways; map04022 cGMP-PKG signaling pathway; map04024 cAMP signaling pathway; map04071 Sphingolipid signaling pathway; map04080 Neuroactive ligand-receptor interaction; map04270 Vascular smooth muscle contraction; map04923 Regulation of lipolysis in adipocytes; map04924 Renin secretion; map05012 Parkinson's disease; map05032 Morphine addiction; map05034 Alcoholism
3.39_268.1040m/z	0.067445954	H123:H120	map00230 Purine metabolism; map01100 Metabolic pathways
3.39_268.1040m/z	0.067445954	H123:H120	NULL
3.39_268.1040m/z	0.067445954	H123:H120	NULL
3.39_268.1040m/z	0.067445954	H123:H120	map04976 Bile secretion
3.39_268.1040m/z	0.067445954	H123:H120	NULL
4.10_296.5217m/z	0.066428664	H123:H120	NA
4.22_566.3234m/z	0.063532315	H123:H120	NULL
3.80_490.1345m/z	0.062076662	H123:H120	map01057 Biosynthesis of type II polyketide products; map01130 Biosynthesis of antibiotics
5.70_729.7097m/z	0.060345479	H123:H120	NA
5.43_643.2211m/z	0.059653013	H123:H120	map01051 Biosynthesis of ansamycins; map01130 Biosynthesis of antibiotics
8.86_573.2884m/z	0.05825704	H123:H120	NULL
8.86_573.2884m/z	0.05825704	H123:H120	NULL
9.20_846.5476m/z	0.055228741	H123:H120	NA
4.52_665.1821m/z	0.052115269	H123:H120	NULL
4.54_965.4481m/z	0.051738927	H123:H120	NULL

4.54_965.4481m/z	0.051738927	H123:H120	NULL
3.51_275.6427m/z	0.051436208	H123:H120	NA
4.67_898.9470m/z	0.050801828	H123:H120	NA
4.54_965.1975m/z	0.049419584	H123:H120	NA
5.92_605.2010m/z	0.047169037	H123:H120	NA
8.93_205.1591m/z	0.046979747	H123:H120	NULL
8.93_205.1591m/z	0.046979747	H123:H120	NULL
8.93_205.1591m/z	0.046979747	H123:H120	NULL
8.87_408.3381n	0.043129266	H123:H120	NA
4.10_444.2797m/z	0.038898764	H123:H120	NA
9.25_844.5341m/z	0.038869836	H123:H120	NA
4.66_790.9094m/z	0.036267704	H123:H120	NA
5.75_675.2604m/z	0.035312972	H123:H120	NULL
5.75_675.2604m/z	0.035312972	H123:H120	NULL
8.86_495.3436m/z	0.034095601	H123:H120	NULL
8.86_495.3436m/z	0.034095601	H123:H120	NULL
8.86_495.3436m/z	0.034095601	H123:H120	NULL
8.86_495.3436m/z	0.034095601	H123:H120	NULL
8.86_495.3436m/z	0.034095601	H123:H120	NULL
8.86_495.3436m/z	0.034095601	H123:H120	NULL
8.86_495.3436m/z	0.034095601	H123:H120	NULL
8.86_495.3436m/z	0.034095601	H123:H120	NULL
8.86_495.3436m/z	0.034095601	H123:H120	NULL
4.36_887.4209m/z	0.033056511	H123:H120	NA
1.68_515.0697m/z	0.032708796	H123:H120	NULL

1.68_515.0697m/z	0.032708796	H123:H120	NULL
5.71_479.1200m/z	0.031554108	H123:H120	NULL
5.71_479.1200m/z	0.031554108	H123:H120	map00253 Tetracycline biosynthesis; map01057 Biosynthesis of type II polyketide products; map01130 Biosynthesis of antibiotics
5.71_479.1200m/z	0.031554108	H123:H120	NULL
5.71_479.1200m/z	0.031554108	H123:H120	map00980 Metabolism of xenobiotics by cytochrome P450
5.71_479.1200m/z	0.031554108	H123:H120	map00980 Metabolism of xenobiotics by cytochrome P450
5.71_479.1200m/z	0.031554108	H123:H120	map00980 Metabolism of xenobiotics by cytochrome P450
5.71_479.1200m/z	0.031554108	H123:H120	map00980 Metabolism of xenobiotics by cytochrome P450
8.86_490.3861m/z	0.031183049	H123:H120	NULL
8.86_490.3861m/z	0.031183049	H123:H120	NULL
8.86_490.3861m/z	0.031183049	H123:H120	NULL
8.86_490.3861m/z	0.031183049	H123:H120	NULL
8.86_490.3861m/z	0.031183049	H123:H120	NULL
8.86_490.3861m/z	0.031183049	H123:H120	NULL
8.86_490.3861m/z	0.031183049	H123:H120	NULL
8.87_616.3161m/z	0.027829802	H123:H120	NULL
8.87_616.3161m/z	0.027829802	H123:H120	map00860 Porphyrin and chlorophyll metabolism; map01110 Biosynthesis of secondary metabolites
9.35_828.5380m/z	0.02698869	H123:H120	NA
5.52_513.1251m/z	0.023239463	H123:H120	map00908 Zeatin biosynthesis
8.87_614.3154m/z	0.022070717	H123:H120	NA
2.61_185.1651m/z	0.013401906	H123:H120	NA
8.19_457.3706m/z	0.011508299	H123:H120	NULL
8.19_457.3706m/z	0.011508299	H123:H120	NULL

8.19_457.3706m/z	0.011508299	H123:H120	NULL
8.19_457.3706m/z	0.011508299	H123:H120	NULL
8.19_457.3706m/z	0.011508299	H123:H120	NULL
8.19_457.3706m/z	0.011508299	H123:H120	NULL
8.19_457.3706m/z	0.011508299	H123:H120	NULL
8.19_457.3706m/z	0.011508299	H123:H120	NULL
8.19_457.3706m/z	0.011508299	H123:H120	NULL
8.87_699.5813n	0.008484594	H123:H120	NA
4.20_272.6750m/z	4.93E-05	H123:H120	NA

Table S16. The top 100 downregulated metabolites in flesh of peach fruit treated with fructose for 12 h.

Compound ID	fold-change	sample	Pathway
4.06_142.0862m/z	0.08008721	H124:H120	NULL
4.06_142.0862m/z	0.08008721	H124:H120	NULL
4.06_142.0862m/z	0.08008721	H124:H120	map00622 Xylene degradation; map00623 Toluene degradation; map00626 Naphthalene degradation; map01100 Metabolic pathways; map01120 Microbial metabolism in diverse environments; map01220 Degradation of aromatic compounds
4.06_142.0862m/z	0.08008721	H124:H120	map00310 Lysine degradation
4.06_142.0862m/z	0.08008721	H124:H120	map00623 Toluene degradation; map01120 Microbial metabolism in diverse environments; map01220 Degradation of aromatic compounds
4.06_142.0862m/z	0.08008721	H124:H120	map00622 Xylene degradation; map00623 Toluene degradation; map00626 Naphthalene degradation; map01100 Metabolic pathways; map01120 Microbial metabolism in diverse environments; map01220 Degradation of aromatic compounds
4.06_142.0862m/z	0.08008721	H124:H120	NULL
4.06_142.0862m/z	0.08008721	H124:H120	NULL
4.06_142.0862m/z	0.08008721	H124:H120	NULL
4.06_142.0862m/z	0.08008721	H124:H120	NULL
4.06_142.0862m/z	0.08008721	H124:H120	NULL
4.06_142.0862m/z	0.08008721	H124:H120	map00960 Tropane, piperidine and pyridine alkaloid biosynthesis
4.06_142.0862m/z	0.08008721	H124:H120	NULL
4.06_142.0862m/z	0.08008721	H124:H120	NULL
4.06_142.0862m/z	0.08008721	H124:H120	NULL
5.52_444.1420n	0.079710261	H124:H120	NULL
5.03_1140.2873m/z	0.079671952	H124:H120	NA
5.46_821.9372m/z	0.072985529	H124:H120	NA

9.77_425.3763m/z	0.072426926	H124:H120	NULL
9.77_425.3763m/z	0.072426926	H124:H120	NULL
9.77_425.3763m/z	0.072426926	H124:H120	NULL
9.77_425.3763m/z	0.072426926	H124:H120	map00905 Brassinosteroid biosynthesis; map01100 Metabolic pathways; map01110 Biosynthesis of secondary metabolites
9.77_425.3763m/z	0.072426926	H124:H120	NULL
9.77_425.3763m/z	0.072426926	H124:H120	NULL
9.77_425.3763m/z	0.072426926	H124:H120	NULL
9.77_425.3763m/z	0.072426926	H124:H120	map00909 Sesquiterpenoid and triterpenoid biosynthesis; map01110 Biosynthesis of secondary metabolites
9.77_425.3763m/z	0.072426926	H124:H120	NULL
9.77_425.3763m/z	0.072426926	H124:H120	NULL
9.77_425.3763m/z	0.072426926	H124:H120	NULL
8.34_760.4398m/z	0.069594147	H124:H120	NA
3.75_556.2388m/z	0.069102759	H124:H120	NA
4.54_965.1975m/z	0.060488236	H124:H120	NA
8.86_573.2884m/z	0.056427712	H124:H120	NULL
8.86_573.2884m/z	0.056427712	H124:H120	NULL
5.96_292.1812m/z	0.056158098	H124:H120	NA
5.52_513.1251m/z	0.055728765	H124:H120	map00908 Zeatin biosynthesis
8.93_205.1591m/z	0.054251267	H124:H120	NULL
8.93_205.1591m/z	0.054251267	H124:H120	NULL
8.93_205.1591m/z	0.054251267	H124:H120	NULL
4.52_665.1821m/z	0.053872608	H124:H120	NULL
5.75_675.2604m/z	0.053837158	H124:H120	NULL

5.75_675.2604m/z	0.053837158	H124:H120	NULL
3.39_268.1040m/z	0.049268186	H124:H120	map00230 Purine metabolism; map01100 Metabolic pathways; map04022 cGMP-PKG signaling pathway; map04024 cAMP signaling pathway; map04071 Sphingolipid signaling pathway; map04080 Neuroactive ligand-receptor interaction; map04270 Vascular smooth muscle contraction; map04923 Regulation of lipolysis in adipocytes; map04924 Renin secretion; map05012 Parkinson's disease; map05032 Morphine addiction; map05034 Alcoholism
3.39_268.1040m/z	0.049268186	H124:H120	map00230 Purine metabolism; map01100 Metabolic pathways
3.39_268.1040m/z	0.049268186	H124:H120	NULL
3.39_268.1040m/z	0.049268186	H124:H120	NULL
3.39_268.1040m/z	0.049268186	H124:H120	map04976 Bile secretion
3.39_268.1040m/z	0.049268186	H124:H120	NULL
5.52_529.1010m/z	0.045913896	H124:H120	map00040 Pentose and glucuronate interconversions; map01100 Metabolic pathways; map02010 ABC transporters
5.52_529.1010m/z	0.045913896	H124:H120	NULL
5.52_529.1010m/z	0.045913896	H124:H120	map00522 Biosynthesis of 12-, 14- and 16-membered macrolides; map00523 Polyketide sugar unit biosynthesis; map01130 Biosynthesis of antibiotics
5.52_529.1010m/z	0.045913896	H124:H120	map00522 Biosynthesis of 12-, 14- and 16-membered macrolides; map00523 Polyketide sugar unit biosynthesis; map01130 Biosynthesis of antibiotics
5.52_529.1010m/z	0.045913896	H124:H120	NULL
4.54_965.4481m/z	0.045169201	H124:H120	NULL
4.54_965.4481m/z	0.045169201	H124:H120	NULL
5.92_605.2010m/z	0.044224085	H124:H120	NA
9.25_844.5341m/z	0.04392054	H124:H120	NA
4.10_444.2797m/z	0.041087663	H124:H120	NA
8.87_408.3381n	0.037989756	H124:H120	NA
9.35_828.5380m/z	0.036352905	H124:H120	NA

4.20_272.6750m/z	0.034416933	H124:H120	NA
4.10_296.5217m/z	0.029603885	H124:H120	NA
8.19_457.3706m/z	0.029123898	H124:H120	NULL
8.19_457.3706m/z	0.029123898	H124:H120	NULL
8.19_457.3706m/z	0.029123898	H124:H120	NULL
8.19_457.3706m/z	0.029123898	H124:H120	NULL
8.19_457.3706m/z	0.029123898	H124:H120	NULL
8.19_457.3706m/z	0.029123898	H124:H120	NULL
8.19_457.3706m/z	0.029123898	H124:H120	NULL
8.19_457.3706m/z	0.029123898	H124:H120	NULL
8.19_457.3706m/z	0.029123898	H124:H120	NULL
8.86_495.3436m/z	0.029112785	H124:H120	NULL
8.86_495.3436m/z	0.029112785	H124:H120	NULL
8.86_495.3436m/z	0.029112785	H124:H120	NULL
8.86_495.3436m/z	0.029112785	H124:H120	NULL
8.86_495.3436m/z	0.029112785	H124:H120	NULL
8.86_495.3436m/z	0.029112785	H124:H120	NULL
8.86_495.3436m/z	0.029112785	H124:H120	NULL
8.86_495.3436m/z	0.029112785	H124:H120	NULL
8.86_495.3436m/z	0.029112785	H124:H120	NULL
8.87_616.3161m/z	0.028403339	H124:H120	NULL
8.87_616.3161m/z	0.028403339	H124:H120	map00860 Porphyrin and chlorophyll metabolism; map01110 Biosynthesis of secondary metabolites
4.67_898.9470m/z	0.025344091	H124:H120	NA
4.66_790.9094m/z	0.022981432	H124:H120	NA

8.87_614.3154m/z	0.017718023	H124:H120	NA
8.86_490.3861m/z	0.016934642	H124:H120	NULL
8.86_490.3861m/z	0.016934642	H124:H120	NULL
8.86_490.3861m/z	0.016934642	H124:H120	NULL
8.86_490.3861m/z	0.016934642	H124:H120	NULL
8.86_490.3861m/z	0.016934642	H124:H120	NULL
8.86_490.3861m/z	0.016934642	H124:H120	NULL
8.86_490.3861m/z	0.016934642	H124:H120	NULL
4.47_555.3643m/z	0.010904018	H124:H120	NA
2.61_185.1651m/z	0.00701448	H124:H120	NA
5.71_479.1200m/z	0.005886842	H124:H120	NULL
5.71_479.1200m/z	0.005886842	H124:H120	map00253 Tetracycline biosynthesis; map01057 Biosynthesis of type II polyketide products; map01130 Biosynthesis of antibiotics
5.71_479.1200m/z	0.005886842	H124:H120	NULL
5.71_479.1200m/z	0.005886842	H124:H120	map00980 Metabolism of xenobiotics by cytochrome P450
5.71_479.1200m/z	0.005886842	H124:H120	map00980 Metabolism of xenobiotics by cytochrome P450
5.71_479.1200m/z	0.005886842	H124:H120	map00980 Metabolism of xenobiotics by cytochrome P450
5.71_479.1200m/z	0.005886842	H124:H120	map00980 Metabolism of xenobiotics by cytochrome P450

Table S17. The top 100 upregulated metabolites in flesh of peach fruit treated with glucose for 24 h.

Compound ID	fold-change	sample	Pathway
3.72_445.2773m/z	1707.830783	H241:H240	NA
0.69_390.1136n	1657.568824	H241:H240	NULL
3.87_719.0752m/z	1193.378808	H241:H240	NULL
3.41_522.1011n	400.3114262	H241:H240	NA
2.76_307.0010m/z	361.1584086	H241:H240	NULL
2.76_307.0010m/z	361.1584086	H241:H240	map00943 Isoflavonoid biosynthesis; map01061 Biosynthesis of phenylpropanoids; map01110 Biosynthesis of secondary metabolites
2.76_307.0010m/z	361.1584086	H241:H240	NULL
3.44_385.9934m/z	301.569962	H241:H240	NA
4.14_339.0281m/z	296.3604982	H241:H240	map00944 Flavone and flavonol biosynthesis
4.14_339.0281m/z	296.3604982	H241:H240	NULL
4.14_339.0281m/z	296.3604982	H241:H240	NULL
4.14_339.0281m/z	296.3604982	H241:H240	NULL
4.14_339.0281m/z	296.3604982	H241:H240	NULL
4.14_339.0281m/z	296.3604982	H241:H240	NULL
4.14_339.0281m/z	296.3604982	H241:H240	map00944 Flavone and flavonol biosynthesis
4.14_339.0281m/z	296.3604982	H241:H240	NULL
4.14_339.0281m/z	296.3604982	H241:H240	map00943 Isoflavonoid biosynthesis
4.14_339.0281m/z	296.3604982	H241:H240	NULL
4.14_339.0281m/z	296.3604982	H241:H240	NULL
4.14_339.0281m/z	296.3604982	H241:H240	NULL
4.14_339.0281m/z	296.3604982	H241:H240	map00943 Isoflavonoid biosynthesis
4.14_339.0281m/z	296.3604982	H241:H240	map00943 Isoflavonoid biosynthesis; map01110 Biosynthesis of secondary metabolites

4.14_339.0281m/z	296.3604982	H241:H240	map00943 Isoflavonoid biosynthesis
4.14_339.0281m/z	296.3604982	H241:H240	map00943 Isoflavonoid biosynthesis; map01110 Biosynthesis of secondary metabolites
4.14_339.0281m/z	296.3604982	H241:H240	NULL
4.14_339.0281m/z	296.3604982	H241:H240	NULL
3.85_549.0628m/z	239.0105983	H241:H240	NULL
3.85_549.0628m/z	239.0105983	H241:H240	NULL
3.85_549.0628m/z	239.0105983	H241:H240	NULL
1.12_325.1426m/z	222.5892959	H241:H240	NULL
1.12_325.1426m/z	222.5892959	H241:H240	NULL
1.12_325.1426m/z	222.5892959	H241:H240	NULL
1.12_325.1426m/z	222.5892959	H241:H240	NULL
1.12_325.1426m/z	222.5892959	H241:H240	NULL
1.12_325.1426m/z	222.5892959	H241:H240	NULL
1.12_325.1426m/z	222.5892959	H241:H240	NULL
1.12_325.1426m/z	222.5892959	H241:H240	NULL
1.12_325.1426m/z	222.5892959	H241:H240	NULL
1.12_325.1426m/z	222.5892959	H241:H240	NULL
1.12_325.1426m/z	222.5892959	H241:H240	NULL
1.12_325.1426m/z	222.5892959	H241:H240	NULL
1.12_325.1426m/z	222.5892959	H241:H240	NULL
1.12_325.1426m/z	222.5892959	H241:H240	NULL
1.12_325.1426m/z	222.5892959	H241:H240	NULL
3.42_387.0007m/z	218.9009145	H241:H240	map00240 Pyrimidine metabolism; map00908 Zeatin biosynthesis; map01100 Metabolic pathways; map04080 Neuroactive ligand-receptor interaction

3.42_387.0007m/z	218.9009145	H241:H240	map00450 Selenocompound metabolism
4.49_755.1092m/z	203.8811519	H241:H240	NA
3.41_523.1083m/z	166.7477232	H241:H240	NA
0.69_196.0645m/z	164.0288988	H241:H240	NULL
3.90_567.0892m/z	162.3916261	H241:H240	map01059 Biosynthesis of enediyne antibiotics; map01130 Biosynthesis of antibiotics
3.87_385.9937n	144.5077929	H241:H240	map00450 Selenocompound metabolism
0.69_325.1480m/z	141.1624356	H241:H240	NA
0.72_328.1178m/z	137.0937319	H241:H240	NULL
0.72_328.1178m/z	137.0937319	H241:H240	NULL
0.72_328.1178m/z	137.0937319	H241:H240	NULL
0.72_328.1178m/z	137.0937319	H241:H240	NULL
0.55_555.7811n	132.9904513	H241:H240	NA
0.67_585.1700n	120.846285	H241:H240	NULL
0.74_317.1376m/z	110.6765711	H241:H240	NULL
0.74_317.1376m/z	110.6765711	H241:H240	NULL
0.74_317.1376m/z	110.6765711	H241:H240	map00520 Amino sugar and nucleotide sugar metabolism
0.74_317.1376m/z	110.6765711	H241:H240	NULL
0.74_317.1376m/z	110.6765711	H241:H240	map00520 Amino sugar and nucleotide sugar metabolism
0.74_317.1376m/z	110.6765711	H241:H240	NULL
2.42_309.0266m/z	104.8927019	H241:H240	map00983 Drug metabolism - other enzymes
2.42_309.0266m/z	104.8927019	H241:H240	NULL
2.42_309.0266m/z	104.8927019	H241:H240	NULL
2.42_309.0266m/z	104.8927019	H241:H240	NULL
2.42_309.0266m/z	104.8927019	H241:H240	NULL
4.06_566.0834n	99.89797363	H241:H240	NA

0.69_310.1202n	96.78657544	H241:H240	map04080 Neuroactive ligand-receptor interaction
0.69_310.1202n	96.78657544	H241:H240	NULL
0.69_310.1202n	96.78657544	H241:H240	NULL
0.59_121.0741n	95.1794859	H241:H240	NULL
3.60_500.0736n	95.05703137	H241:H240	NA
3.87_501.0804m/z	90.73301451	H241:H240	map00680 Methane metabolism; map01100 Metabolic pathways; map01120 Microbial metabolism in diverse environments; map01200 Carbon metabolism
3.87_501.0804m/z	90.73301451	H241:H240	NULL
3.87_501.0804m/z	90.73301451	H241:H240	NULL
3.87_501.0804m/z	90.73301451	H241:H240	NULL
3.87_501.0804m/z	90.73301451	H241:H240	NULL
3.87_501.0804m/z	90.73301451	H241:H240	NULL
3.42_107.0128m/z	88.38563063	H241:H240	map00361 Chlorocyclohexane and chlorobenzene degradation; map00627 Aminobenzoate degradation; map01100 Metabolic pathways; map01120 Microbial metabolism in diverse environments
3.42_107.0128m/z	88.38563063	H241:H240	NULL
0.74_639.1689m/z	85.08553402	H241:H240	map00860 Porphyrin and chlorophyll metabolism; map01060 Biosynthesis of plant secondary metabolites; map01100 Metabolic pathways; map01110 Biosynthesis of secondary metabolites; map02010 ABC transporters; map04978 Mineral absorption
0.74_639.1689m/z	85.08553402	H241:H240	NULL
2.37_287.0547m/z	78.86124397	H241:H240	map00941 Flavonoid biosynthesis; map00944 Flavone and flavonol biosynthesis; map01100 Metabolic pathways; map01110 Biosynthesis of secondary metabolites
2.37_287.0547m/z	78.86124397	H241:H240	map00941 Flavonoid biosynthesis; map01061 Biosynthesis of phenylpropanoids; map01100 Metabolic pathways; map01110 Biosynthesis of secondary metabolites

2.37_287.0547m/z	78.86124397	H241:H240	map00941 Flavonoid biosynthesis; map00944 Flavone and flavonol biosynthesis; map01061 Biosynthesis of phenylpropanoids; map01100 Metabolic pathways; map01110 Biosynthesis of secondary metabolites
2.37_287.0547m/z	78.86124397	H241:H240	map00941 Flavonoid biosynthesis; map01110 Biosynthesis of secondary metabolites
2.37_287.0547m/z	78.86124397	H241:H240	NULL
2.37_287.0547m/z	78.86124397	H241:H240	NULL
2.37_287.0547m/z	78.86124397	H241:H240	NULL
2.37_287.0547m/z	78.86124397	H241:H240	NULL
2.37_287.0547m/z	78.86124397	H241:H240	NULL
2.37_287.0547m/z	78.86124397	H241:H240	NULL
2.37_287.0547m/z	78.86124397	H241:H240	NULL
2.37_287.0547m/z	78.86124397	H241:H240	map00943 Isoflavonoid biosynthesis
2.37_287.0547m/z	78.86124397	H241:H240	NULL
2.37_287.0547m/z	78.86124397	H241:H240	NULL
2.37_287.0547m/z	78.86124397	H241:H240	NULL

Table S18. The top 100 upregulated metabolites in flesh of peach fruit treated with sucrose for 24 h.

Compound ID	fold-change	sample	Pathway
3.72_445.2773m/z	2022.125421	H242:H240	NA
3.87_719.0752m/z	1606.760393	H242:H240	NULL
0.69_390.1136n	944.6000113	H242:H240	NULL
3.41_522.1011n	819.3487956	H242:H240	NA
3.41_523.1083m/z	391.195785	H242:H240	NA
2.76_307.0010m/z	357.0318069	H242:H240	NULL
2.76_307.0010m/z	357.0318069	H242:H240	map00943 Isoflavonoid biosynthesis; map01061 Biosynthesis of phenylpropanoids; map01110 Biosynthesis of secondary metabolites
2.76_307.0010m/z	357.0318069	H242:H240	NULL
3.44_385.9934m/z	348.1287149	H242:H240	NA
3.42_387.0007m/z	265.3649879	H242:H240	map00240 Pyrimidine metabolism; map00908 Zeatin biosynthesis; map01100 Metabolic pathways; map04080 Neuroactive ligand-receptor interaction
3.42_387.0007m/z	265.3649879	H242:H240	map00450 Selenocompound metabolism
1.12_325.1426m/z	247.0504427	H242:H240	NULL
1.12_325.1426m/z	247.0504427	H242:H240	NULL
1.12_325.1426m/z	247.0504427	H242:H240	NULL
1.12_325.1426m/z	247.0504427	H242:H240	NULL
1.12_325.1426m/z	247.0504427	H242:H240	NULL
1.12_325.1426m/z	247.0504427	H242:H240	NULL
1.12_325.1426m/z	247.0504427	H242:H240	NULL
1.12_325.1426m/z	247.0504427	H242:H240	NULL
1.12_325.1426m/z	247.0504427	H242:H240	NULL
1.12_325.1426m/z	247.0504427	H242:H240	NULL

1.12_325.1426m/z	247.0504427	H242:H240	NULL
1.12_325.1426m/z	247.0504427	H242:H240	NULL
1.12_325.1426m/z	247.0504427	H242:H240	NULL
1.12_325.1426m/z	247.0504427	H242:H240	NULL
1.12_325.1426m/z	247.0504427	H242:H240	NULL
1.12_325.1426m/z	247.0504427	H242:H240	NULL
3.85_549.0628m/z	235.3032233	H242:H240	NULL
3.85_549.0628m/z	235.3032233	H242:H240	NULL
3.85_549.0628m/z	235.3032233	H242:H240	NULL
3.87_385.9937n	173.2669066	H242:H240	map00450 Selenocompound metabolism
0.69_196.0645m/z	172.2737572	H242:H240	NULL
2.37_287.0547m/z	166.3306341	H242:H240	map00941 Flavonoid biosynthesis; map00944 Flavone and flavonol biosynthesis; map01100 Metabolic pathways; map01110 Biosynthesis of secondary metabolites
2.37_287.0547m/z	166.3306341	H242:H240	map00941 Flavonoid biosynthesis; map01061 Biosynthesis of phenylpropanoids; map01100 Metabolic pathways; map01110 Biosynthesis of secondary metabolites
2.37_287.0547m/z	166.3306341	H242:H240	map00941 Flavonoid biosynthesis; map00944 Flavone and flavonol biosynthesis; map01061 Biosynthesis of phenylpropanoids; map01100 Metabolic pathways; map01110 Biosynthesis of secondary metabolites
2.37_287.0547m/z	166.3306341	H242:H240	map00941 Flavonoid biosynthesis; map01110 Biosynthesis of secondary metabolites
2.37_287.0547m/z	166.3306341	H242:H240	NULL
2.37_287.0547m/z	166.3306341	H242:H240	NULL
2.37_287.0547m/z	166.3306341	H242:H240	NULL
2.37_287.0547m/z	166.3306341	H242:H240	NULL
2.37_287.0547m/z	166.3306341	H242:H240	NULL
2.37_287.0547m/z	166.3306341	H242:H240	NULL

2.37_287.0547m/z	166.3306341	H242:H240	NULL
2.37_287.0547m/z	166.3306341	H242:H240	map00943 Isoflavonoid biosynthesis
2.37_287.0547m/z	166.3306341	H242:H240	NULL
2.37_287.0547m/z	166.3306341	H242:H240	NULL
2.37_287.0547m/z	166.3306341	H242:H240	NULL
4.49_755.1092m/z	144.7748211	H242:H240	NA
0.72_328.1178m/z	132.9758784	H242:H240	NULL
0.72_328.1178m/z	132.9758784	H242:H240	NULL
0.72_328.1178m/z	132.9758784	H242:H240	NULL
0.72_328.1178m/z	132.9758784	H242:H240	NULL
3.42_107.0128m/z	101.9469296	H242:H240	map00361 Chlorocyclohexane and chlorobenzene degradation; map00627 Aminobenzoate degradation; map01100 Metabolic pathways; map01120 Microbial metabolism in diverse environments
3.42_107.0128m/z	101.9469296	H242:H240	NULL
2.42_309.0266m/z	97.30065806	H242:H240	map00983 Drug metabolism - other enzymes
2.42_309.0266m/z	97.30065806	H242:H240	NULL
2.42_309.0266m/z	97.30065806	H242:H240	NULL
2.42_309.0266m/z	97.30065806	H242:H240	NULL
2.42_309.0266m/z	97.30065806	H242:H240	NULL
0.69_310.1202n	97.08174213	H242:H240	map04080 Neuroactive ligand-receptor interaction
0.69_310.1202n	97.08174213	H242:H240	NULL
0.69_310.1202n	97.08174213	H242:H240	NULL
3.41_330.0376n	89.97750737	H242:H240	NULL
3.41_330.0376n	89.97750737	H242:H240	NULL
3.41_330.0376n	89.97750737	H242:H240	NULL

3.90_567.0892m/z	88.97412256	H242:H240	map01059 Biosynthesis of enediyne antibiotics; map01130 Biosynthesis of antibiotics
3.87_170.0218n	88.41669323	H242:H240	map00627 Aminobenzoate degradation; map01061 Biosynthesis of phenylpropanoids; map01120 Microbial metabolism in diverse environments
0.80_251.0087m/z	87.58289837	H242:H240	NA
3.42_170.0217n	84.24352023	H242:H240	map00627 Aminobenzoate degradation; map01061 Biosynthesis of phenylpropanoids; map01120 Microbial metabolism in diverse environments
0.74_639.1689m/z	79.04520402	H242:H240	map00860 Porphyrin and chlorophyll metabolism; map01060 Biosynthesis of plant secondary metabolites; map01100 Metabolic pathways; map01110 Biosynthesis of secondary metabolites; map02010 ABC transporters; map04978 Mineral absorption
0.74_639.1689m/z	79.04520402	H242:H240	NULL
4.54_588.1325n	77.77397166	H242:H240	NULL
0.69_325.1480m/z	75.39421785	H242:H240	NA
4.14_339.0281m/z	72.38813327	H242:H240	map00944 Flavone and flavonol biosynthesis
4.14_339.0281m/z	72.38813327	H242:H240	NULL
4.14_339.0281m/z	72.38813327	H242:H240	NULL
4.14_339.0281m/z	72.38813327	H242:H240	NULL
4.14_339.0281m/z	72.38813327	H242:H240	NULL
4.14_339.0281m/z	72.38813327	H242:H240	NULL
4.14_339.0281m/z	72.38813327	H242:H240	map00944 Flavone and flavonol biosynthesis
4.14_339.0281m/z	72.38813327	H242:H240	NULL
4.14_339.0281m/z	72.38813327	H242:H240	map00943 Isoflavonoid biosynthesis
4.14_339.0281m/z	72.38813327	H242:H240	NULL
4.14_339.0281m/z	72.38813327	H242:H240	NULL
4.14_339.0281m/z	72.38813327	H242:H240	NULL
4.14_339.0281m/z	72.38813327	H242:H240	map00943 Isoflavonoid biosynthesis

4.14_339.0281m/z	72.38813327	H242:H240	map00943 Isoflavonoid biosynthesis; map01110 Biosynthesis of secondary metabolites
4.14_339.0281m/z	72.38813327	H242:H240	map00943 Isoflavonoid biosynthesis
4.14_339.0281m/z	72.38813327	H242:H240	map00943 Isoflavonoid biosynthesis; map01110 Biosynthesis of secondary metabolites
4.14_339.0281m/z	72.38813327	H242:H240	NULL
4.14_339.0281m/z	72.38813327	H242:H240	NULL
2.90_171.0289m/z	70.2709426	H242:H240	map00627 Aminobenzoate degradation; map01061 Biosynthesis of phenylpropanoids; map01120 Microbial metabolism in diverse environments
2.90_171.0289m/z	70.2709426	H242:H240	map00300 Lysine biosynthesis; map01100 Metabolic pathways; map01120 Microbial metabolism in diverse environments; map01130 Biosynthesis of antibiotics; map01210 2-Oxocarboxylic acid metabolism; map01230 Biosynthesis of amino acids
2.90_171.0289m/z	70.2709426	H242:H240	map00640 Propanoate metabolism
2.90_171.0289m/z	70.2709426	H242:H240	NULL
2.90_171.0289m/z	70.2709426	H242:H240	NULL
2.90_171.0289m/z	70.2709426	H242:H240	NULL
2.90_171.0289m/z	70.2709426	H242:H240	NULL
2.90_171.0289m/z	70.2709426	H242:H240	map00640 Propanoate metabolism
3.58_595.0894m/z	70.1024643	H242:H240	NA
0.59_121.0741n	69.01210839	H242:H240	NULL

Table S19. The top 100 upregulated metabolites in flesh of peach fruit treated with sorbitol for 24 h.

Compound ID	fold-change	sample	Pathway
3.72_445.2773m/z	1876.653792	H243:H240	NA
0.69_390.1136n	1433.507744	H243:H240	NULL
3.87_719.0752m/z	1138.047294	H243:H240	NULL
3.41_522.1011n	418.7350176	H243:H240	NA
3.44_385.9934m/z	292.0234018	H243:H240	NA
2.76_307.0010m/z	281.7405599	H243:H240	NULL
2.76_307.0010m/z	281.7405599	H243:H240	map00943 Isoflavonoid biosynthesis; map01061 Biosynthesis of phenylpropanoids; map01110 Biosynthesis of secondary metabolites
2.76_307.0010m/z	281.7405599	H243:H240	NULL
1.12_325.1426m/z	279.9412039	H243:H240	NULL
1.12_325.1426m/z	279.9412039	H243:H240	NULL
1.12_325.1426m/z	279.9412039	H243:H240	NULL
1.12_325.1426m/z	279.9412039	H243:H240	NULL
1.12_325.1426m/z	279.9412039	H243:H240	NULL
1.12_325.1426m/z	279.9412039	H243:H240	NULL
1.12_325.1426m/z	279.9412039	H243:H240	NULL
1.12_325.1426m/z	279.9412039	H243:H240	NULL
1.12_325.1426m/z	279.9412039	H243:H240	NULL
1.12_325.1426m/z	279.9412039	H243:H240	NULL
1.12_325.1426m/z	279.9412039	H243:H240	NULL
1.12_325.1426m/z	279.9412039	H243:H240	NULL
1.12_325.1426m/z	279.9412039	H243:H240	NULL
1.12_325.1426m/z	279.9412039	H243:H240	NULL

1.12_325.1426m/z	279.9412039	H243:H240	NULL
1.12_325.1426m/z	279.9412039	H243:H240	NULL
4.49_755.1092m/z	226.7112809	H243:H240	NA
3.42_387.0007m/z	223.5242067	H243:H240	map00240 Pyrimidine metabolism; map00908 Zeatin biosynthesis; map01100 Metabolic pathways; map04080 Neuroactive ligand-receptor interaction
3.42_387.0007m/z	223.5242067	H243:H240	map00450 Selenocompound metabolism
3.41_523.1083m/z	210.1818178	H243:H240	NA
3.85_549.0628m/z	187.7290031	H243:H240	NULL
3.85_549.0628m/z	187.7290031	H243:H240	NULL
3.85_549.0628m/z	187.7290031	H243:H240	NULL
0.69_196.0645m/z	182.294974	H243:H240	NULL
0.72_328.1178m/z	178.6920075	H243:H240	NULL
0.72_328.1178m/z	178.6920075	H243:H240	NULL
0.72_328.1178m/z	178.6920075	H243:H240	NULL
0.72_328.1178m/z	178.6920075	H243:H240	NULL
3.87_385.9937n	145.8419238	H243:H240	map00450 Selenocompound metabolism
0.69_325.1480m/z	139.8661151	H243:H240	NA
4.14_339.0281m/z	123.6737805	H243:H240	map00944 Flavone and flavonol biosynthesis
4.14_339.0281m/z	123.6737805	H243:H240	NULL
4.14_339.0281m/z	123.6737805	H243:H240	NULL
4.14_339.0281m/z	123.6737805	H243:H240	NULL
4.14_339.0281m/z	123.6737805	H243:H240	NULL
4.14_339.0281m/z	123.6737805	H243:H240	NULL
4.14_339.0281m/z	123.6737805	H243:H240	map00944 Flavone and flavonol biosynthesis
4.14_339.0281m/z	123.6737805	H243:H240	NULL

4.14_339.0281m/z	123.6737805	H243:H240	map00943 Isoflavonoid biosynthesis
4.14_339.0281m/z	123.6737805	H243:H240	NULL
4.14_339.0281m/z	123.6737805	H243:H240	NULL
4.14_339.0281m/z	123.6737805	H243:H240	NULL
4.14_339.0281m/z	123.6737805	H243:H240	map00943 Isoflavonoid biosynthesis
4.14_339.0281m/z	123.6737805	H243:H240	map00943 Isoflavonoid biosynthesis; map01110 Biosynthesis of secondary metabolites
4.14_339.0281m/z	123.6737805	H243:H240	map00943 Isoflavonoid biosynthesis
4.14_339.0281m/z	123.6737805	H243:H240	map00943 Isoflavonoid biosynthesis; map01110 Biosynthesis of secondary metabolites
4.14_339.0281m/z	123.6737805	H243:H240	NULL
4.14_339.0281m/z	123.6737805	H243:H240	NULL
0.69_310.1202n	113.7686334	H243:H240	map04080 Neuroactive ligand-receptor interaction
0.69_310.1202n	113.7686334	H243:H240	NULL
0.69_310.1202n	113.7686334	H243:H240	NULL
0.74_639.1689m/z	109.2165489	H243:H240	map00860 Porphyrin and chlorophyll metabolism; map01060 Biosynthesis of plant secondary metabolites; map01100 Metabolic pathways; map01110 Biosynthesis of secondary metabolites; map02010 ABC transporters; map04978 Mineral absorption
0.74_639.1689m/z	109.2165489	H243:H240	NULL
0.55_555.7811n	95.07187423	H243:H240	NA
4.32_527.0457m/z	92.50068319	H243:H240	NA
0.74_317.1376m/z	88.46501326	H243:H240	NULL
0.74_317.1376m/z	88.46501326	H243:H240	NULL
0.74_317.1376m/z	88.46501326	H243:H240	map00520 Amino sugar and nucleotide sugar metabolism
0.74_317.1376m/z	88.46501326	H243:H240	NULL
0.74_317.1376m/z	88.46501326	H243:H240	map00520 Amino sugar and nucleotide sugar metabolism
0.74_317.1376m/z	88.46501326	H243:H240	NULL

2.37_287.0547m/z	86.14052385	H243:H240	map00941 Flavonoid biosynthesis; map00944 Flavone and flavonol biosynthesis; map01100 Metabolic pathways; map01110 Biosynthesis of secondary metabolites
2.37_287.0547m/z	86.14052385	H243:H240	map00941 Flavonoid biosynthesis; map01061 Biosynthesis of phenylpropanoids; map01100 Metabolic pathways; map01110 Biosynthesis of secondary metabolites
2.37_287.0547m/z	86.14052385	H243:H240	map00941 Flavonoid biosynthesis; map00944 Flavone and flavonol biosynthesis; map01061 Biosynthesis of phenylpropanoids; map01100 Metabolic pathways; map01110 Biosynthesis of secondary metabolites
2.37_287.0547m/z	86.14052385	H243:H240	map00941 Flavonoid biosynthesis; map01110 Biosynthesis of secondary metabolites
2.37_287.0547m/z	86.14052385	H243:H240	NULL
2.37_287.0547m/z	86.14052385	H243:H240	NULL
2.37_287.0547m/z	86.14052385	H243:H240	NULL
2.37_287.0547m/z	86.14052385	H243:H240	NULL
2.37_287.0547m/z	86.14052385	H243:H240	NULL
2.37_287.0547m/z	86.14052385	H243:H240	NULL
2.37_287.0547m/z	86.14052385	H243:H240	NULL
2.37_287.0547m/z	86.14052385	H243:H240	map00943 Isoflavonoid biosynthesis
2.37_287.0547m/z	86.14052385	H243:H240	NULL
2.37_287.0547m/z	86.14052385	H243:H240	NULL
2.37_287.0547m/z	86.14052385	H243:H240	NULL
0.59_121.0741n	85.20175525	H243:H240	NULL
3.42_107.0128m/z	85.17088007	H243:H240	map00361 Chlorocyclohexane and chlorobenzene degradation; map00627 Aminobenzoate degradation; map01100 Metabolic pathways; map01120 Microbial metabolism in diverse environments
3.42_107.0128m/z	85.17088007	H243:H240	NULL
9.05_725.5959n	84.06641684	H243:H240	NA

3.90_567.0892m/z	78.42168855	H243:H240	map01059 Biosynthesis of enediyne antibiotics; map01130 Biosynthesis of antibiotics
8.44_382.3680m/z	78.28176375	H243:H240	NA
0.80_251.0087m/z	73.01426628	H243:H240	NA
0.75_468.1117m/z	72.96398642	H243:H240	NA
2.42_309.0266m/z	72.86326156	H243:H240	map00983 Drug metabolism - other enzymes
2.42_309.0266m/z	72.86326156	H243:H240	NULL
2.42_309.0266m/z	72.86326156	H243:H240	NULL
2.42_309.0266m/z	72.86326156	H243:H240	NULL
2.42_309.0266m/z	72.86326156	H243:H240	NULL
3.58_595.0894m/z	72.38601356	H243:H240	NA
3.87_170.0218n	70.39601711	H243:H240	map00627 Aminobenzoate degradation; map01061 Biosynthesis of phenylpropanoids; map01120 Microbial metabolism in diverse environments
3.42_170.0217n	70.35434647	H243:H240	map00627 Aminobenzoate degradation; map01061 Biosynthesis of phenylpropanoids; map01120 Microbial metabolism in diverse environments
0.67_234.0200m/z	69.27961211	H243:H240	NULL

Table S20. The top 100 upregulated metabolites in flesh of peach fruit treated with fructose for 24 h.

Compound ID	fold-change	sample	Pathway
3.87_719.0752m/z	1443.562785	H244:H240	NULL
0.69_390.1136n	717.9150444	H244:H240	NULL
3.41_522.1011n	504.0140677	H244:H240	NA
3.44_385.9934m/z	311.5636467	H244:H240	NA
3.41_523.1083m/z	286.9753428	H244:H240	NA
3.72_445.2773m/z	251.6527183	H244:H240	NA
3.85_549.0628m/z	240.5568061	H244:H240	NULL
3.85_549.0628m/z	240.5568061	H244:H240	NULL
3.85_549.0628m/z	240.5568061	H244:H240	NULL
3.42_387.0007m/z	235.6798628	H244:H240	map00240 Pyrimidine metabolism; map00908 Zeatin biosynthesis; map01100 Metabolic pathways; map04080 Neuroactive ligand-receptor interaction
3.42_387.0007m/z	235.6798628	H244:H240	map00450 Selenocompound metabolism
2.76_307.0010m/z	195.7110982	H244:H240	NULL
2.76_307.0010m/z	195.7110982	H244:H240	map00943 Isoflavonoid biosynthesis; map01061 Biosynthesis of phenylpropanoids; map01110 Biosynthesis of secondary metabolites
2.76_307.0010m/z	195.7110982	H244:H240	NULL
1.12_325.1426m/z	178.8482686	H244:H240	NULL
1.12_325.1426m/z	178.8482686	H244:H240	NULL
1.12_325.1426m/z	178.8482686	H244:H240	NULL
1.12_325.1426m/z	178.8482686	H244:H240	NULL
1.12_325.1426m/z	178.8482686	H244:H240	NULL
1.12_325.1426m/z	178.8482686	H244:H240	NULL
1.12_325.1426m/z	178.8482686	H244:H240	NULL

1.12_325.1426m/z	178.8482686	H244:H240	NULL
1.12_325.1426m/z	178.8482686	H244:H240	NULL
1.12_325.1426m/z	178.8482686	H244:H240	NULL
1.12_325.1426m/z	178.8482686	H244:H240	NULL
1.12_325.1426m/z	178.8482686	H244:H240	NULL
1.12_325.1426m/z	178.8482686	H244:H240	NULL
1.12_325.1426m/z	178.8482686	H244:H240	NULL
1.12_325.1426m/z	178.8482686	H244:H240	NULL
1.12_325.1426m/z	178.8482686	H244:H240	NULL
8.44_382.3680m/z	170.2953535	H244:H240	NA
3.87_385.9937n	161.5053593	H244:H240	map00450 Selenocompound metabolism
4.14_339.0281m/z	153.5086063	H244:H240	map00944 Flavone and flavonol biosynthesis
4.14_339.0281m/z	153.5086063	H244:H240	NULL
4.14_339.0281m/z	153.5086063	H244:H240	NULL
4.14_339.0281m/z	153.5086063	H244:H240	NULL
4.14_339.0281m/z	153.5086063	H244:H240	NULL
4.14_339.0281m/z	153.5086063	H244:H240	NULL
4.14_339.0281m/z	153.5086063	H244:H240	map00944 Flavone and flavonol biosynthesis
4.14_339.0281m/z	153.5086063	H244:H240	NULL
4.14_339.0281m/z	153.5086063	H244:H240	map00943 Isoflavonoid biosynthesis
4.14_339.0281m/z	153.5086063	H244:H240	NULL
4.14_339.0281m/z	153.5086063	H244:H240	NULL
4.14_339.0281m/z	153.5086063	H244:H240	NULL
4.14_339.0281m/z	153.5086063	H244:H240	map00943 Isoflavonoid biosynthesis
4.14_339.0281m/z	153.5086063	H244:H240	map00943 Isoflavonoid biosynthesis; map01110 Biosynthesis of secondary metabolites

4.14_339.0281m/z	153.5086063	H244:H240	map00943 Isoflavonoid biosynthesis
4.14_339.0281m/z	153.5086063	H244:H240	map00943 Isoflavonoid biosynthesis; map01110 Biosynthesis of secondary metabolites
4.14_339.0281m/z	153.5086063	H244:H240	NULL
4.14_339.0281m/z	153.5086063	H244:H240	NULL
4.49_755.1092m/z	144.4004979	H244:H240	NA
0.69_196.0645m/z	142.6165656	H244:H240	NULL
0.55_555.7811n	130.5497129	H244:H240	NA
0.72_328.1178m/z	126.3589584	H244:H240	NULL
0.72_328.1178m/z	126.3589584	H244:H240	NULL
0.72_328.1178m/z	126.3589584	H244:H240	NULL
0.72_328.1178m/z	126.3589584	H244:H240	NULL
3.90_567.0892m/z	124.653653	H244:H240	map01059 Biosynthesis of enediyne antibiotics; map01130 Biosynthesis of antibiotics
3.60_500.0736n	115.2022285	H244:H240	NA
0.69_310.1202n	103.3847974	H244:H240	map04080 Neuroactive ligand-receptor interaction
0.69_310.1202n	103.3847974	H244:H240	NULL
0.69_310.1202n	103.3847974	H244:H240	NULL
2.37_287.0547m/z	102.0027191	H244:H240	map00941 Flavonoid biosynthesis; map00944 Flavone and flavonol biosynthesis; map01100 Metabolic pathways; map01110 Biosynthesis of secondary metabolites
2.37_287.0547m/z	102.0027191	H244:H240	map00941 Flavonoid biosynthesis; map01061 Biosynthesis of phenylpropanoids; map01100 Metabolic pathways; map01110 Biosynthesis of secondary metabolites
2.37_287.0547m/z	102.0027191	H244:H240	map00941 Flavonoid biosynthesis; map00944 Flavone and flavonol biosynthesis; map01061 Biosynthesis of phenylpropanoids; map01100 Metabolic pathways; map01110 Biosynthesis of secondary metabolites
2.37_287.0547m/z	102.0027191	H244:H240	map00941 Flavonoid biosynthesis; map01110 Biosynthesis of secondary metabolites
2.37_287.0547m/z	102.0027191	H244:H240	NULL

2.37_287.0547m/z	102.0027191	H244:H240	NULL
2.37_287.0547m/z	102.0027191	H244:H240	NULL
2.37_287.0547m/z	102.0027191	H244:H240	NULL
2.37_287.0547m/z	102.0027191	H244:H240	NULL
2.37_287.0547m/z	102.0027191	H244:H240	NULL
2.37_287.0547m/z	102.0027191	H244:H240	NULL
2.37_287.0547m/z	102.0027191	H244:H240	map00943 Isoflavonoid biosynthesis
2.37_287.0547m/z	102.0027191	H244:H240	NULL
2.37_287.0547m/z	102.0027191	H244:H240	NULL
2.37_287.0547m/z	102.0027191	H244:H240	NULL
3.42_107.0128m/z	93.98467086	H244:H240	map00361 Chlorocyclohexane and chlorobenzene degradation; map00627 Aminobenzoate degradation; map01100 Metabolic pathways; map01120 Microbial metabolism in diverse environments
3.42_107.0128m/z	93.98467086	H244:H240	NULL
4.54_588.1325n	89.39570097	H244:H240	NULL
3.87_170.0218n	82.10534657	H244:H240	map00627 Aminobenzoate degradation; map01061 Biosynthesis of phenylpropanoids; map01120 Microbial metabolism in diverse environments
0.74_639.1689m/z	81.52182096	H244:H240	map00860 Porphyrin and chlorophyll metabolism; map01060 Biosynthesis of plant secondary metabolites; map01100 Metabolic pathways; map01110 Biosynthesis of secondary metabolites; map02010 ABC transporters; map04978 Mineral absorption
0.74_639.1689m/z	81.52182096	H244:H240	NULL
0.69_325.1480m/z	80.50795377	H244:H240	NA
3.87_501.0804m/z	77.65410888	H244:H240	map00680 Methane metabolism; map01100 Metabolic pathways; map01120 Microbial metabolism in diverse environments; map01200 Carbon metabolism
3.87_501.0804m/z	77.65410888	H244:H240	NULL

3.87_501.0804m/z	77.65410888	H244:H240	NULL
3.87_501.0804m/z	77.65410888	H244:H240	NULL
3.87_501.0804m/z	77.65410888	H244:H240	NULL
3.87_501.0804m/z	77.65410888	H244:H240	NULL
4.06_566.0834n	75.81174349	H244:H240	NA
3.42_170.0217n	73.51410937	H244:H240	map00627 Aminobenzoate degradation; map01061 Biosynthesis of phenylpropanoids; map01120 Microbial metabolism in diverse environments
0.78_376.0066m/z	68.40795073	H244:H240	NULL
0.78_376.0066m/z	68.40795073	H244:H240	map00404 Staurosporine biosynthesis; map01130 Biosynthesis of antibiotics
9.05_725.5959n	66.00871946	H244:H240	NA
3.41_330.0376n	63.77751273	H244:H240	NULL
3.41_330.0376n	63.77751273	H244:H240	NULL
3.41_330.0376n	63.77751273	H244:H240	NULL
3.58_595.0894m/z	60.01497073	H244:H240	NA
3.75_501.0808m/z	58.89273667	H244:H240	map00680 Methane metabolism; map01100 Metabolic pathways; map01120 Microbial metabolism in diverse environments; map01200 Carbon metabolism
3.75_501.0808m/z	58.89273667	H244:H240	NULL

Table S21. The top 100 downregulated metabolites in flesh of peach fruit treated with glucose for 24 h.

Compound ID	fold-change	sample	Pathway
5.23_541.1684m/z	0.154411778	H241:H240	NULL
5.23_541.1684m/z	0.154411778	H241:H240	NULL
5.23_541.1684m/z	0.154411778	H241:H240	NULL
4.08_481.1936m/z	0.153946642	H241:H240	map01060 Biosynthesis of plant secondary metabolites; map01063 Biosynthesis of alkaloids derived from shikimate pathway
4.91_550.3048m/z	0.153927665	H241:H240	NULL
4.91_550.3048m/z	0.153927665	H241:H240	NULL
4.91_550.3048m/z	0.153927665	H241:H240	NULL
4.91_550.3048m/z	0.153927665	H241:H240	NULL
5.22_679.2785m/z	0.15145942	H241:H240	map01057 Biosynthesis of type II polyketide products; map01130 Biosynthesis of antibiotics
5.52_338.2804m/z	0.151336538	H241:H240	NA
0.55_796.6159m/z	0.151098574	H241:H240	NA
6.04_855.4473m/z	0.14881315	H241:H240	NA
8.69_701.2027m/z	0.145850591	H241:H240	NA
1.11_622.1646m/z	0.145352522	H241:H240	NA
5.46_747.3056m/z	0.144881796	H241:H240	NA
7.09_812.4229m/z	0.140338351	H241:H240	NULL
4.08_353.2082m/z	0.139719533	H241:H240	map00140 Steroid hormone biosynthesis; map01100 Metabolic pathways; map04913 Ovarian steroidogenesis
4.08_353.2082m/z	0.139719533	H241:H240	NULL
4.08_353.2082m/z	0.139719533	H241:H240	map00140 Steroid hormone biosynthesis; map01100 Metabolic pathways; map04925 Aldosterone synthesis and secretion
4.08_353.2082m/z	0.139719533	H241:H240	map00140 Steroid hormone biosynthesis

4.08_353.2082m/z	0.139719533	H241:H240	NULL
4.08_353.2082m/z	0.139719533	H241:H240	NULL
4.08_353.2082m/z	0.139719533	H241:H240	map00140 Steroid hormone biosynthesis
4.08_353.2082m/z	0.139719533	H241:H240	map00960 Tropane, piperidine and pyridine alkaloid biosynthesis; map01064 Biosynthesis of alkaloids derived from ornithine, lysine and nicotinic acid; map01110 Biosynthesis of secondary metabolites
4.08_353.2082m/z	0.139719533	H241:H240	NULL
4.08_353.2082m/z	0.139719533	H241:H240	NULL
4.08_353.2082m/z	0.139719533	H241:H240	NULL
4.08_353.2082m/z	0.139719533	H241:H240	NULL
4.08_353.2082m/z	0.139719533	H241:H240	NULL
4.08_353.2082m/z	0.139719533	H241:H240	NULL
4.08_353.2082m/z	0.139719533	H241:H240	NULL
4.08_353.2082m/z	0.139719533	H241:H240	map00591 Linoleic acid metabolism
4.08_353.2082m/z	0.139719533	H241:H240	map00591 Linoleic acid metabolism
4.08_353.2082m/z	0.139719533	H241:H240	NULL
4.08_353.2082m/z	0.139719533	H241:H240	NULL
4.08_353.2082m/z	0.139719533	H241:H240	NULL
4.08_353.2082m/z	0.139719533	H241:H240	NULL
4.08_353.2082m/z	0.139719533	H241:H240	NULL
4.08_353.2082m/z	0.139719533	H241:H240	NULL
4.08_353.2082m/z	0.139719533	H241:H240	NULL
4.08_353.2082m/z	0.139719533	H241:H240	NULL
4.08_353.2082m/z	0.139719533	H241:H240	NULL
4.08_353.2082m/z	0.139719533	H241:H240	map00073 Cutin, suberine and wax biosynthesis

4.08_353.2082m/z	0.139719533	H241:H240	NULL
4.08_353.2082m/z	0.139719533	H241:H240	NULL
4.08_353.2082m/z	0.139719533	H241:H240	NULL
5.31_728.3841m/z	0.135546045	H241:H240	NULL
5.31_728.3841m/z	0.135546045	H241:H240	NULL
5.31_728.3841m/z	0.135546045	H241:H240	NULL
3.66_490.1343m/z	0.135525016	H241:H240	map01057 Biosynthesis of type II polyketide products; map01130 Biosynthesis of antibiotics
4.22_377.8842m/z	0.134188757	H241:H240	NA
4.67_898.9470m/z	0.133531871	H241:H240	NA
5.01_998.9740m/z	0.133185681	H241:H240	NA
5.62_579.3169m/z	0.133082366	H241:H240	NULL
6.26_588.3588m/z	0.132339168	H241:H240	map00522 Biosynthesis of 12-, 14- and 16-membered macrolides; map01130 Biosynthesis of antibiotics
4.54_965.4481m/z	0.131590569	H241:H240	NULL
4.54_965.4481m/z	0.131590569	H241:H240	NULL
5.01_998.4726m/z	0.127769331	H241:H240	NA
4.54_965.1975m/z	0.125795365	H241:H240	NA
4.22_566.3234m/z	0.124651288	H241:H240	NULL
4.33_630.3427m/z	0.122736858	H241:H240	NA
3.39_118.0281n	0.120612613	H241:H240	NA
4.35_800.0387m/z	0.120402811	H241:H240	NA
7.13_344.3159m/z	0.116211585	H241:H240	NULL
4.10_296.5217m/z	0.109968408	H241:H240	NA
4.89_688.3863m/z	0.108245651	H241:H240	NULL
4.36_887.4209m/z	0.107963908	H241:H240	NA

5.75_835.4418n	0.107204898	H241:H240	NA
0.57_683.9164n	0.105832595	H241:H240	NA
5.46_472.1246m/z	0.105577086	H241:H240	NA
6.72_637.3216m/z	0.104516529	H241:H240	NA
6.72_704.3612n	0.103277119	H241:H240	NA
5.43_643.2211m/z	0.102701174	H241:H240	map01051 Biosynthesis of ansamycins; map01130 Biosynthesis of antibiotics
5.52_953.4509m/z	0.100622288	H241:H240	NA
5.75_835.7761n	0.097727612	H241:H240	NA
4.42_770.3773m/z	0.087903277	H241:H240	NA
4.42_326.6683n	0.085330927	H241:H240	NA
4.12_178.1337m/z	0.08366209	H241:H240	map00380 Tryptophan metabolism; map00901 Indole alkaloid biosynthesis; map01060 Biosynthesis of plant secondary metabolites; map01063 Biosynthesis of alkaloids derived from shikimate pathway; map01100 Metabolic pathways; map01110 Biosynthesis of secondary metabolites; map04080 Neuroactive ligand-receptor interaction
4.12_178.1337m/z	0.08366209	H241:H240	NULL
4.12_178.1337m/z	0.08366209	H241:H240	NULL
4.12_178.1337m/z	0.08366209	H241:H240	map00960 Tropane, piperidine and pyridine alkaloid biosynthesis; map01064 Biosynthesis of alkaloids derived from ornithine, lysine and nicotinic acid; map01110 Biosynthesis of secondary metabolites
4.12_178.1337m/z	0.08366209	H241:H240	map01065 Biosynthesis of alkaloids derived from histidine and purine; map01110 Biosynthesis of secondary metabolites
4.12_178.1337m/z	0.08366209	H241:H240	NULL
4.12_178.1337m/z	0.08366209	H241:H240	map00760 Nicotinate and nicotinamide metabolism; map01120 Microbial metabolism in diverse environments
5.75_836.1103n	0.0807157	H241:H240	NA

3.80_490.1345m/z	0.07741574	H241:H240	map01057 Biosynthesis of type II polyketide products; map01130 Biosynthesis of antibiotics
5.52_485.2370m/z	0.075922335	H241:H240	NULL
4.52_665.1821m/z	0.073842279	H241:H240	NULL
1.68_515.0697m/z	0.071073245	H241:H240	NULL
1.68_515.0697m/z	0.071073245	H241:H240	NULL
4.10_444.2797m/z	0.065283229	H241:H240	NA
4.27_294.1897m/z	0.064975097	H241:H240	map00330 Arginine and proline metabolism
5.52_445.2208n	0.061536539	H241:H240	NULL
9.35_828.5380m/z	0.059659648	H241:H240	NA
3.39_268.1040m/z	0.057514572	H241:H240	map00230 Purine metabolism; map01100 Metabolic pathways; map04022 cGMP-PKG signaling pathway; map04024 cAMP signaling pathway; map04071 Sphingolipid signaling pathway; map04080 Neuroactive ligand-receptor interaction; map04270 Vascular smooth muscle contraction; map04923 Regulation of lipolysis in adipocytes; map04924 Renin secretion; map05012 Parkinson's disease; map05032 Morphine addiction; map05034 Alcoholism
3.39_268.1040m/z	0.057514572	H241:H240	map00230 Purine metabolism; map01100 Metabolic pathways
3.39_268.1040m/z	0.057514572	H241:H240	NULL
3.39_268.1040m/z	0.057514572	H241:H240	NULL
3.39_268.1040m/z	0.057514572	H241:H240	map04976 Bile secretion
3.39_268.1040m/z	0.057514572	H241:H240	NULL
4.42_770.8794m/z	0.038137324	H241:H240	NA

Table S22. The top 100 downregulated metabolites in flesh of peach fruit treated with sucrose for 24 h.

Compound ID	fold-change	sample	Pathway
5.62_295.2380m/z	0.155866057	H242:H240	map01130 Biosynthesis of antibiotics
5.62_295.2380m/z	0.155866057	H242:H240	NULL
5.62_295.2380m/z	0.155866057	H242:H240	map00904 Diterpenoid biosynthesis; map01110 Biosynthesis of secondary metabolites
5.62_295.2380m/z	0.155866057	H242:H240	map00904 Diterpenoid biosynthesis; map01110 Biosynthesis of secondary metabolites
5.62_295.2380m/z	0.155866057	H242:H240	map00904 Diterpenoid biosynthesis; map01110 Biosynthesis of secondary metabolites
5.62_295.2380m/z	0.155866057	H242:H240	NULL
9.25_727.6075m/z	0.155637002	H242:H240	NA
0.57_550.9907m/z	0.153630352	H242:H240	NA
5.68_487.3125m/z	0.149019052	H242:H240	NA
3.69_429.0962m/z	0.148720196	H242:H240	NULL
3.69_429.0962m/z	0.148720196	H242:H240	NULL
3.69_429.0962m/z	0.148720196	H242:H240	NULL
3.69_429.0962m/z	0.148720196	H242:H240	NULL
3.69_429.0962m/z	0.148720196	H242:H240	NULL
1.68_515.0697m/z	0.148522811	H242:H240	NULL
1.68_515.0697m/z	0.148522811	H242:H240	NULL
9.30_263.5277m/z	0.147153509	H242:H240	NA
5.46_472.1246m/z	0.145997259	H242:H240	NA
0.56_838.1049m/z	0.145291677	H242:H240	NA
9.35_828.5380m/z	0.144850052	H242:H240	NA
5.58_470.1707m/z	0.143990756	H242:H240	NA

5.01_998.9740m/z	0.142699894	H242:H240	NA
4.33_630.3427m/z	0.142558234	H242:H240	NA
5.31_728.3841m/z	0.139471508	H242:H240	NULL
5.31_728.3841m/z	0.139471508	H242:H240	NULL
5.31_728.3841m/z	0.139471508	H242:H240	NULL
5.46_747.3056m/z	0.138404601	H242:H240	NA
5.49_606.8039m/z	0.136559108	H242:H240	NA
6.04_855.4473m/z	0.136164841	H242:H240	NA
4.43_797.3792m/z	0.131663999	H242:H240	NA
3.69_357.2130m/z	0.13094806	H242:H240	NULL
3.69_357.2130m/z	0.13094806	H242:H240	NULL
3.69_357.2130m/z	0.13094806	H242:H240	map01130 Biosynthesis of antibiotics
4.94_655.9981m/z	0.13087093	H242:H240	NA
4.12_178.1337m/z	0.128347921	H242:H240	map00380 Tryptophan metabolism; map00901 Indole alkaloid biosynthesis; map01060 Biosynthesis of plant secondary metabolites; map01063 Biosynthesis of alkaloids derived from shikimate pathway; map01100 Metabolic pathways; map01110 Biosynthesis of secondary metabolites; map04080 Neuroactive ligand-receptor interaction
4.12_178.1337m/z	0.128347921	H242:H240	NULL
4.12_178.1337m/z	0.128347921	H242:H240	NULL
4.12_178.1337m/z	0.128347921	H242:H240	map00960 Tropane, piperidine and pyridine alkaloid biosynthesis; map01064 Biosynthesis of alkaloids derived from ornithine, lysine and nicotinic acid; map01110 Biosynthesis of secondary metabolites
4.12_178.1337m/z	0.128347921	H242:H240	map01065 Biosynthesis of alkaloids derived from histidine and purine; map01110 Biosynthesis of secondary metabolites
4.12_178.1337m/z	0.128347921	H242:H240	NULL

4.12_178.1337m/z	0.128347921	H242:H240	map00760 Nicotinate and nicotinamide metabolism; map01120 Microbial metabolism in diverse environments
9.30_571.0044m/z	0.127502429	H242:H240	NA
4.42_326.6683n	0.123926651	H242:H240	NA
5.75_835.7761n	0.123841977	H242:H240	NA
4.54_965.4481m/z	0.121463836	H242:H240	NULL
4.54_965.4481m/z	0.121463836	H242:H240	NULL
4.89_688.3863m/z	0.116319387	H242:H240	NULL
5.01_998.4726m/z	0.112662093	H242:H240	NA
4.72_324.2281m/z	0.111870185	H242:H240	NULL
4.72_324.2281m/z	0.111870185	H242:H240	NULL
4.72_324.2281m/z	0.111870185	H242:H240	NULL
4.30_180.1359m/z	0.108896554	H242:H240	NA
4.10_296.5217m/z	0.106097359	H242:H240	NA
3.39_118.0281n	0.1060846	H242:H240	NA
9.36_491.3708m/z	0.105471375	H242:H240	NULL
9.36_491.3708m/z	0.105471375	H242:H240	NULL
9.36_491.3708m/z	0.105471375	H242:H240	NULL
9.30_587.9797m/z	0.104249323	H242:H240	NA
4.36_887.4209m/z	0.103880757	H242:H240	NA
6.72_637.3216m/z	0.103268889	H242:H240	NA
4.33_420.8983m/z	0.10151792	H242:H240	NA
9.05_399.8404m/z	0.100484829	H242:H240	NA
4.52_665.1821m/z	0.096540924	H242:H240	NULL
6.26_588.8606m/z	0.092988203	H242:H240	NA

6.27_705.4132m/z	0.092350609	H242:H240	NA
6.33_694.3554m/z	0.089863205	H242:H240	NA
4.42_770.8794m/z	0.089613761	H242:H240	NA
6.72_704.3612n	0.088618385	H242:H240	NA
5.75_836.1103n	0.083415013	H242:H240	NA
5.75_835.4418n	0.079735847	H242:H240	NA
3.80_490.1345m/z	0.078756539	H242:H240	map01057 Biosynthesis of type II polyketide products; map01130 Biosynthesis of antibiotics
3.88_414.2707m/z	0.077839754	H242:H240	NULL
4.88_269.2224m/z	0.075361139	H242:H240	NULL
4.88_269.2224m/z	0.075361139	H242:H240	NULL
5.52_485.2370m/z	0.075336249	H242:H240	NULL
5.04_652.3582m/z	0.071219766	H242:H240	NA
3.98_499.2875m/z	0.069984461	H242:H240	NULL
3.98_499.2875m/z	0.069984461	H242:H240	map00522 Biosynthesis of 12-, 14- and 16-membered macrolides; map01130 Biosynthesis of antibiotics
4.27_294.1897m/z	0.064256543	H242:H240	map00330 Arginine and proline metabolism
3.83_476.2390m/z	0.062254539	H242:H240	map00403 Indole diterpene alkaloid biosynthesis; map01110 Biosynthesis of secondary metabolites
9.87_609.2712m/z	0.061042566	H242:H240	map00860 Porphyrin and chlorophyll metabolism; map01110 Biosynthesis of secondary metabolites
9.87_609.2712m/z	0.061042566	H242:H240	map00860 Porphyrin and chlorophyll metabolism; map01110 Biosynthesis of secondary metabolites
9.87_609.2712m/z	0.061042566	H242:H240	map00983 Drug metabolism - other enzymes
9.87_609.2712m/z	0.061042566	H242:H240	map00860 Porphyrin and chlorophyll metabolism; map01110 Biosynthesis of secondary metabolites

9.87_609.2712m/z	0.061042566	H242:H240	NULL
6.33_605.1908m/z	0.060687469	H242:H240	NULL
6.33_605.1908m/z	0.060687469	H242:H240	NULL
6.33_605.1908m/z	0.060687469	H242:H240	map02010 ABC transporters
3.66_490.1343m/z	0.060612642	H242:H240	map01057 Biosynthesis of type II polyketide products; map01130 Biosynthesis of antibiotics
9.66_636.2583n	0.058327544	H242:H240	NA
5.52_445.2208n	0.057169989	H242:H240	NULL
10.89_325.3345n	0.053744254	H242:H240	NA
4.42_770.3773m/z	0.051046715	H242:H240	NA
10.07_378.7900m/z	0.049558906	H242:H240	NULL
3.39_268.1040m/z	0.041970468	H242:H240	map00230 Purine metabolism; map01100 Metabolic pathways; map04022 cGMP-PKG signaling pathway; map04024 cAMP signaling pathway; map04071 Sphingolipid signaling pathway; map04080 Neuroactive ligand-receptor interaction; map04270 Vascular smooth muscle contraction; map04923 Regulation of lipolysis in adipocytes; map04924 Renin secretion; map05012 Parkinson's disease; map05032 Morphine addiction; map05034 Alcoholism
3.39_268.1040m/z	0.041970468	H242:H240	map00230 Purine metabolism; map01100 Metabolic pathways
3.39_268.1040m/z	0.041970468	H242:H240	NULL
3.39_268.1040m/z	0.041970468	H242:H240	NULL
3.39_268.1040m/z	0.041970468	H242:H240	map04976 Bile secretion
3.39_268.1040m/z	0.041970468	H242:H240	NULL
7.39_673.2882m/z	0.029469233	H242:H240	NA

Table S23. The top 100 downregulated metabolites in flesh of peach fruit treated with sorbitol for 24 h.

Compound ID	fold-change	sample	Pathway
6.45_638.3289n	0.140567734	H243:H240	NA
7.23_532.3031m/z	0.139822208	H243:H240	NA
5.52_953.4509m/z	0.139070394	H243:H240	NA
3.69_429.0962m/z	0.138655871	H243:H240	NULL
3.69_429.0962m/z	0.138655871	H243:H240	NULL
3.69_429.0962m/z	0.138655871	H243:H240	NULL
3.69_429.0962m/z	0.138655871	H243:H240	NULL
3.69_429.0962m/z	0.138655871	H243:H240	NULL
0.56_838.1049m/z	0.138204706	H243:H240	NA
4.72_324.2281m/z	0.138161138	H243:H240	NULL
4.72_324.2281m/z	0.138161138	H243:H240	NULL
4.72_324.2281m/z	0.138161138	H243:H240	NULL
5.10_695.3782m/z	0.137054564	H243:H240	NA
3.29_231.1341m/z	0.136911985	H243:H240	NULL
3.29_231.1341m/z	0.136911985	H243:H240	NULL
3.29_231.1341m/z	0.136911985	H243:H240	NULL
3.29_231.1341m/z	0.136911985	H243:H240	NULL
3.29_231.1341m/z	0.136911985	H243:H240	NULL
3.29_231.1341m/z	0.136911985	H243:H240	map05204 Chemical carcinogenesis
3.29_231.1341m/z	0.136911985	H243:H240	map02024 Quorum sensing
5.29_493.2644m/z	0.136697296	H243:H240	NULL
5.29_493.2644m/z	0.136697296	H243:H240	map00524 Neomycin, kanamycin and gentamicin biosynthesis; map01130 Biosynthesis of antibiotics

4.91_550.3048m/z	0.13584479	H243:H240	NULL
4.91_550.3048m/z	0.13584479	H243:H240	NULL
4.91_550.3048m/z	0.13584479	H243:H240	NULL
4.91_550.3048m/z	0.13584479	H243:H240	NULL
5.96_292.1812m/z	0.132797997	H243:H240	NA
5.55_517.2258m/z	0.132299904	H243:H240	NULL
4.86_746.3581m/z	0.128420805	H243:H240	NA
3.99_133.5332m/z	0.12769851	H243:H240	NA
4.92_483.2437m/z	0.127092401	H243:H240	NULL
5.81_732.3935m/z	0.12474432	H243:H240	NA
4.18_532.2617m/z	0.122183693	H243:H240	NULL
4.18_532.2617m/z	0.122183693	H243:H240	NULL
5.01_998.4726m/z	0.120803848	H243:H240	NA
5.25_649.2664m/z	0.117761841	H243:H240	map00860 Porphyrin and chlorophyll metabolism; map01110 Biosynthesis of secondary metabolites
4.35_800.0387m/z	0.117683684	H243:H240	NA
9.96_284.3793m/z	0.116195535	H243:H240	NA
6.72_704.3612n	0.10983559	H243:H240	NA
5.26_145.1015m/z	0.109589753	H243:H240	NA
6.57_676.3454m/z	0.108371724	H243:H240	NA
3.39_118.0281n	0.105526757	H243:H240	NA
4.36_887.4209m/z	0.105410749	H243:H240	NA
1.11_622.1646m/z	0.104136726	H243:H240	NA
4.43_797.3792m/z	0.101210555	H243:H240	NA
5.75_836.1103n	0.100919993	H243:H240	NA

3.69_357.2130m/z	0.100616834	H243:H240	NULL
3.69_357.2130m/z	0.100616834	H243:H240	NULL
3.69_357.2130m/z	0.100616834	H243:H240	map01130 Biosynthesis of antibiotics
6.33_694.3554m/z	0.094179912	H243:H240	NA
4.54_965.1975m/z	0.092869146	H243:H240	NA
5.04_652.3582m/z	0.090045225	H243:H240	NA
5.43_643.2211m/z	0.08981852	H243:H240	map01051 Biosynthesis of ansamycins; map01130 Biosynthesis of antibiotics
4.96_587.2808m/z	0.089268286	H243:H240	map00860 Porphyrin and chlorophyll metabolism; map01110 Biosynthesis of secondary metabolites
4.96_587.2808m/z	0.089268286	H243:H240	map00860 Porphyrin and chlorophyll metabolism; map01110 Biosynthesis of secondary metabolites
4.96_587.2808m/z	0.089268286	H243:H240	NULL
4.96_587.2808m/z	0.089268286	H243:H240	NULL
4.96_587.2808m/z	0.089268286	H243:H240	NULL
4.96_587.2808m/z	0.089268286	H243:H240	NULL
4.96_587.2808m/z	0.089268286	H243:H240	map00983 Drug metabolism - other enzymes
4.54_965.4481m/z	0.088289317	H243:H240	NULL
4.54_965.4481m/z	0.088289317	H243:H240	NULL
4.42_770.8794m/z	0.088152514	H243:H240	NA
6.74_493.2796m/z	0.087768008	H243:H240	NULL
0.53_208.0868m/z	0.087736156	H243:H240	NULL
6.04_855.4473m/z	0.08448874	H243:H240	NA
3.66_490.1343m/z	0.084185606	H243:H240	map01057 Biosynthesis of type II polyketide products; map01130 Biosynthesis of antibiotics
5.52_485.2370m/z	0.083220215	H243:H240	NULL
4.42_770.3773m/z	0.080611259	H243:H240	NA

4.33_630.3427m/z	0.078791918	H243:H240	NA
3.98_499.2875m/z	0.077466098	H243:H240	NULL
3.98_499.2875m/z	0.077466098	H243:H240	map00522 Biosynthesis of 12-, 14- and 16-membered macrolides; map01130 Biosynthesis of antibiotics
3.76_239.1754m/z	0.07741401	H243:H240	NULL
3.76_239.1754m/z	0.07741401	H243:H240	NULL
3.76_239.1754m/z	0.07741401	H243:H240	NULL
6.41_692.3580n	0.075535149	H243:H240	NA
6.27_705.4132m/z	0.074150303	H243:H240	NA
4.27_294.1897m/z	0.073202572	H243:H240	map00330 Arginine and proline metabolism
5.75_835.7761n	0.071970378	H243:H240	NA
5.68_487.3125m/z	0.070659346	H243:H240	NA
4.22_566.3234m/z	0.069006709	H243:H240	NULL
5.75_835.4418n	0.067460489	H243:H240	NA
6.60_634.2989m/z	0.065508073	H243:H240	NULL
6.60_634.2989m/z	0.065508073	H243:H240	NULL
4.10_296.5217m/z	0.064250135	H243:H240	NA
6.26_588.8606m/z	0.064222851	H243:H240	NA
4.76_688.3871m/z	0.063623752	H243:H240	NULL
6.72_637.3216m/z	0.061968709	H243:H240	NA
5.52_445.2208n	0.060164782	H243:H240	NULL
1.68_515.0697m/z	0.060040785	H243:H240	NULL
1.68_515.0697m/z	0.060040785	H243:H240	NULL
4.10_444.2797m/z	0.058009213	H243:H240	NA
3.80_490.1345m/z	0.055111807	H243:H240	map01057 Biosynthesis of type II polyketide products; map01130 Biosynthesis of antibiotics

3.88_414.2707m/z	0.048286828	H243:H240	NULL
3.39_268.1040m/z	0.04092959	H243:H240	map00230 Purine metabolism; map01100 Metabolic pathways; map04022 cGMP-PKG signaling pathway; map04024 cAMP signaling pathway; map04071 Sphingolipid signaling pathway; map04080 Neuroactive ligand-receptor interaction; map04270 Vascular smooth muscle contraction; map04923 Regulation of lipolysis in adipocytes; map04924 Renin secretion; map05012 Parkinson's disease; map05032 Morphine addiction; map05034 Alcoholism
3.39_268.1040m/z	0.04092959	H243:H240	map00230 Purine metabolism; map01100 Metabolic pathways
3.39_268.1040m/z	0.04092959	H243:H240	NULL
3.39_268.1040m/z	0.04092959	H243:H240	NULL
3.39_268.1040m/z	0.04092959	H243:H240	map04976 Bile secretion
3.39_268.1040m/z	0.04092959	H243:H240	NULL
4.52_665.1821m/z	0.021468796	H243:H240	NULL

Table S24. The top 100 downregulated metabolites in flesh of peach fruit treated with fructose for 24 h.

Compound ID	fold-change	sample	Pathway
5.01_998.2219m/z	0.164803222	H244:H240	NA
5.58_470.1707m/z	0.162645362	H244:H240	NA
9.29_718.1192m/z	0.161116666	H244:H240	NA
5.55_517.2258m/z	0.158801117	H244:H240	NULL
0.57_550.9907m/z	0.154812448	H244:H240	NA
4.89_688.3863m/z	0.154038942	H244:H240	NULL
5.46_472.1246m/z	0.15199022	H244:H240	NA
5.75_835.4418n	0.150286895	H244:H240	NA
3.99_133.5332m/z	0.149985046	H244:H240	NA
3.54_219.6056m/z	0.149261798	H244:H240	NA
4.12_178.1337m/z	0.147630144	H244:H240	map00380 Tryptophan metabolism; map00901 Indole alkaloid biosynthesis; map01060 Biosynthesis of plant secondary metabolites; map01063 Biosynthesis of alkaloids derived from shikimate pathway; map01100 Metabolic pathways; map01110 Biosynthesis of secondary metabolites; map04080 Neuroactive ligand-receptor interaction
4.12_178.1337m/z	0.147630144	H244:H240	NULL
4.12_178.1337m/z	0.147630144	H244:H240	NULL
4.12_178.1337m/z	0.147630144	H244:H240	map00960 Tropane, piperidine and pyridine alkaloid biosynthesis; map01064 Biosynthesis of alkaloids derived from ornithine, lysine and nicotinic acid; map01110 Biosynthesis of secondary metabolites
4.12_178.1337m/z	0.147630144	H244:H240	map01065 Biosynthesis of alkaloids derived from histidine and purine; map01110 Biosynthesis of secondary metabolites
4.12_178.1337m/z	0.147630144	H244:H240	NULL
4.12_178.1337m/z	0.147630144	H244:H240	map00760 Nicotinate and nicotinamide metabolism; map01120 Microbial metabolism in diverse environments

5.75_522.1854m/z	0.147488999	H244:H240	NULL
4.08_481.1936m/z	0.146069886	H244:H240	map01060 Biosynthesis of plant secondary metabolites; map01063 Biosynthesis of alkaloids derived from shikimate pathway
3.69_429.0962m/z	0.14471289	H244:H240	NULL
3.69_429.0962m/z	0.14471289	H244:H240	NULL
3.69_429.0962m/z	0.14471289	H244:H240	NULL
3.69_429.0962m/z	0.14471289	H244:H240	NULL
3.69_429.0962m/z	0.14471289	H244:H240	NULL
6.64_691.3341n	0.144300851	H244:H240	NULL
4.96_587.2808m/z	0.143585311	H244:H240	map00860 Porphyrin and chlorophyll metabolism; map01110 Biosynthesis of secondary metabolites
4.96_587.2808m/z	0.143585311	H244:H240	map00860 Porphyrin and chlorophyll metabolism; map01110 Biosynthesis of secondary metabolites
4.96_587.2808m/z	0.143585311	H244:H240	NULL
4.96_587.2808m/z	0.143585311	H244:H240	NULL
4.96_587.2808m/z	0.143585311	H244:H240	NULL
4.96_587.2808m/z	0.143585311	H244:H240	NULL
4.96_587.2808m/z	0.143585311	H244:H240	map00983 Drug metabolism - other enzymes
6.74_493.2796m/z	0.143159015	H244:H240	NULL
5.52_953.4509m/z	0.142634816	H244:H240	NA
9.30_263.5277m/z	0.140271851	H244:H240	NA
5.81_732.3935m/z	0.139928729	H244:H240	NA
5.96_292.1812m/z	0.138849272	H244:H240	NA
4.35_800.3727m/z	0.137908752	H244:H240	NA
5.26_145.1015m/z	0.136670081	H244:H240	NA

3.98_499.2875m/z	0.136214973	H244:H240	NULL
3.98_499.2875m/z	0.136214973	H244:H240	map00522 Biosynthesis of 12-, 14- and 16-membered macrolides; map01130 Biosynthesis of antibiotics
5.75_836.1103n	0.13451072	H244:H240	NA
4.35_800.0387m/z	0.128951087	H244:H240	NA
4.30_180.1359m/z	0.128138907	H244:H240	NA
6.60_634.2989m/z	0.126779352	H244:H240	NULL
6.60_634.2989m/z	0.126779352	H244:H240	NULL
5.25_649.2664m/z	0.125992932	H244:H240	map00860 Porphyrin and chlorophyll metabolism; map01110 Biosynthesis of secondary metabolites
4.33_420.8983m/z	0.1241307	H244:H240	NA
4.42_770.3773m/z	0.122621849	H244:H240	NA
5.46_747.3056m/z	0.122283616	H244:H240	NA
4.22_566.3234m/z	0.120709316	H244:H240	NULL
6.04_855.4473m/z	0.119199493	H244:H240	NA
4.10_296.5217m/z	0.115523159	H244:H240	NA
3.39_118.0281n	0.115241433	H244:H240	NA
5.04_652.3582m/z	0.109552946	H244:H240	NA
6.33_694.3554m/z	0.108113025	H244:H240	NA
3.80_490.1345m/z	0.106419715	H244:H240	map01057 Biosynthesis of type II polyketide products; map01130 Biosynthesis of antibiotics
4.54_965.1975m/z	0.102443755	H244:H240	NA
4.20_272.6750m/z	0.096785057	H244:H240	NA
4.10_444.2797m/z	0.095210155	H244:H240	NA
4.86_746.3581m/z	0.094598633	H244:H240	NA
9.30_587.9797m/z	0.093524951	H244:H240	NA

3.69_357.2130m/z	0.093149589	H244:H240	NULL
3.69_357.2130m/z	0.093149589	H244:H240	NULL
3.69_357.2130m/z	0.093149589	H244:H240	map01130 Biosynthesis of antibiotics
8.87_699.5813n	0.090830177	H244:H240	NA
6.57_676.3454m/z	0.085261287	H244:H240	NA
0.56_838.1049m/z	0.08445816	H244:H240	NA
4.36_887.4209m/z	0.082286475	H244:H240	NA
4.54_965.4481m/z	0.081741106	H244:H240	NULL
4.54_965.4481m/z	0.081741106	H244:H240	NULL
6.74_695.3263m/z	0.078180024	H244:H240	NULL
4.42_770.8794m/z	0.073444075	H244:H240	NA
4.27_294.1897m/z	0.072305594	H244:H240	map00330 Arginine and proline metabolism
6.27_705.4132m/z	0.07077923	H244:H240	NA
5.52_485.2370m/z	0.069717165	H244:H240	NULL
9.44_868.7074m/z	0.068547165	H244:H240	NA
1.11_622.1646m/z	0.067877167	H244:H240	NA
6.26_588.8606m/z	0.067118014	H244:H240	NA
9.96_284.3793m/z	0.06606487	H244:H240	NA
6.72_637.3216m/z	0.061847644	H244:H240	NA
6.72_704.3612n	0.059879615	H244:H240	NA
5.52_445.2208n	0.054078706	H244:H240	NULL
1.68_515.0697m/z	0.052463627	H244:H240	NULL
1.68_515.0697m/z	0.052463627	H244:H240	NULL
9.05_399.8404m/z	0.052259533	H244:H240	NA
4.43_797.3792m/z	0.048589837	H244:H240	NA

12.23_651.8616m/z	0.046507444	H244:H240	NA
3.39_268.1040m/z	0.045798284	H244:H240	map00230 Purine metabolism; map01100 Metabolic pathways; map04022 cGMP-PKG signaling pathway; map04024 cAMP signaling pathway; map04071 Sphingolipid signaling pathway; map04080 Neuroactive ligand-receptor interaction; map04270 Vascular smooth muscle contraction; map04923 Regulation of lipolysis in adipocytes; map04924 Renin secretion; map05012 Parkinson's disease; map05032 Morphine addiction; map05034 Alcoholism
3.39_268.1040m/z	0.045798284	H244:H240	map00230 Purine metabolism; map01100 Metabolic pathways
3.39_268.1040m/z	0.045798284	H244:H240	NULL
3.39_268.1040m/z	0.045798284	H244:H240	NULL
3.39_268.1040m/z	0.045798284	H244:H240	map04976 Bile secretion
3.39_268.1040m/z	0.045798284	H244:H240	NULL
9.35_828.5380m/z	0.039865694	H244:H240	NA
4.52_665.1821m/z	0.035558284	H244:H240	NULL
2.86_156.1385m/z	0.009865834	H244:H240	map00960 Tropane, piperidine and pyridine alkaloid biosynthesis; map01064 Biosynthesis of alkaloids derived from ornithine, lysine and nicotinic acid; map01110 Biosynthesis of secondary metabolites
2.86_156.1385m/z	0.009865834	H244:H240	NULL
2.86_156.1385m/z	0.009865834	H244:H240	NULL
2.86_156.1385m/z	0.009865834	H244:H240	map00592 alpha-Linolenic acid metabolism; map01110 Biosynthesis of secondary metabolites
9.60_205.6319m/z	0.002055977	H244:H240	NA
